# Molecular-docking-guided design, palladium-catalyzed synthesis and anticancer activity of paclitaxel-benzoxazoles hybrids

**DOI:** 10.1038/s41598-022-14172-3

**Published:** 2022-06-15

**Authors:** Ting Jiang, Ya-Nan Cao, Jin-Bu Xu, Feng Gao, Ling-Li Zheng

**Affiliations:** 1 Department of Pharmacy, The First Afflicted Hospital of Chengdu Medical College, No. 278, Baoguang Rd, Xindu Region, Chengdu, 610500 People’s Republic of China; 2 School of Life Science and Engineering, Southwest Jiaotong University, No. 111, Erhuan Rd, Chengdu, 610031 People’s Republic of China

**Keywords:** Medicinal chemistry, Natural product synthesis

## Abstract

A series of new paclitaxel-benzoxazoles hybrids were designed based on both the molecular docking mode of beta-tubulin with paclitaxel derivatives (**7a** and **7g**), and the activity-structure relationship of C-13 side chain in paclitaxel. Palladium-catalyzed direct Csp^2^–H arylation of benzoxazoles with different aryl-bromides was used as the key synthetic strategy for the aryl-benzoxazoles moieties in the hybrids. Twenty-six newly synthesized hybrids were screened for their antiproliferative activity against human cancer cell lines such as human breast cancer cells (MDA-MB-231) and liver hepatocellular cells (HepG2) by the MTT assay and results were compared with paclitaxel. Interestingly, most hybrids (**7a**–**7e**, **7i**, **7k**, **7l**, **7A**, **7B**, **7D** and **7E**) showed significantly active against both cell lines at concentration of 50 µM, which indicated that the hybrid strategy is effective to get structural simplified paclitaxel analogues with high anti-tumor activity.

## Introduction

**Figure 1:**
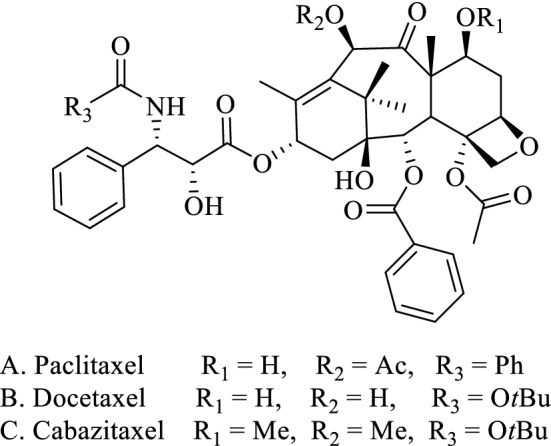
Structures of paclitaxel, docetaxel, and cabazitaxel.

Cancer, one of the most formidable common diseases, remains threat to human health and is responsible for increase in the mortality rate all over the world. It has been estimated that close to 550,000 deaths caused by this disease according to the epidemiological and clinical investigations^1–3^. Although many effective approaches including radiation, surgery and targeted therapy have been exploited to cure for cancer, natural products or its derivatives have been of particular interest as cancer chemotherapeutic agents in the past few decades. Up to date, more than 100 anticancer agents with varied mechanisms of action have been developed from diverse natural origin^4–6^. Paclitaxel (Taxol^®^), the naturally diterpenoid extract from the bark of *Taxus brevifolia* Nutt, and the semisynthetic analogues docetaxel (Taxotere®) and cabazitaxel (Jevana®) (Fig. 1), act as the momentous chemotherapeutics in current clinical treatment of breast cancer, non-small cell lung cancer, ovarian cancer and prostate cancer^7^. They accelerate the irreversible assembly of tubulin into microtubules and thus stimulate the apoptosis of tumor cells through disrupting mitosis to exert their therapeutic effect^8,9^. Since the poor solubility, undesirable side effects and drug resistance have immensely limited the clinical application of paclitaxel, and the highly complex structure have prevented large-scale producing paclitaxel as well^10,11^. Therefore, researchers have maintained a considerable interest in the discovery and development of novel anticancer drugs because of the aforementioned disadvantages of the paclitaxel.

Hybrids are based on the principle of combining partial or whole structures in order to create new and possibly more safe and active molecular entities^12^. Some studies revealed that paclitaxel-natural-products based hybrids were potential agents which could be possible to extend and strengthen the medical utility of paclitaxel^13,14^. Numerous structure–activity- relationship studies (SARs) indicated that the C-13 side chain is an indispensable part for its antitumor activity^15–19^. In our previous studies, paclitaxel mimics possessing C-13 side chains showed moderate antitumor activities^19–21^. Literature data has been established that nitrogen containing heterocyclic moiety plays an important role in designing new class of structural entities for medicinal applications^22^. Therefore, we were encouraged to design and synthesize paclitaxel-nitrogen containing heterocyclic moiety hybrids in which the intricate baccatin-core is structurally replaced by simplified structures, to find new mimics with low side effects and high efficacy. Initially, docking studies of a series of paclitaxel-nitrogen containing heterocyclic moiety hybrids with beta-tubulin were carried out. Paclitaxel-benzoxazoles hybrids **7a** and **7g** were revealed to have good biding affinity with beta-tubulin, indicating this type of hybrids may be potential anticancer lead compounds. Moreover, some benzoxazole derivatives also are reported as anticancer agents^23,24^. Thus, as an ongoing part of our research on paclitaxel analogues^19–21^, in current research, twenty-six paclitaxel-benzoxazoles hybrids were designed guided by molecular docking study. The aryl-benzoxazoles moieties of hybrids were synthesized via palladium-catalyzed direct Csp^2^–H arylation of benzoxazoles with different aryl-bromides. The biological activities of twenty-six hybrids were evaluated.

## Results and discussion

### Molecular docking study

It was believed that the simplified paclitaxel analogues with better antitumor activities could accelerate the polymerization of tubulin and stabilize the resultant microtubules to apoptosis through cell-signaling cascade either. In our continued work on finding paclitaxel analogues with better antitumor activities, the docking studies of a series of paclitaxel-nitrogen containing heterocyclic moiety hybrids with beta-tubulin were carried out. To our delight, the paclitaxel-benzoxazoles hybrids **7a** and **7g** showed good binding affinity with beta-tubulin. -Cdocker Interaction Energy values of compounds **7a** and **7g** with beta-tubulin were 52.9245 and 54.6571 kcal/mol, respectively, along with -Cdocker Energy values of them were 38.4268 and 38.2471 kcal/mol, respectively.

**Figure 2:**
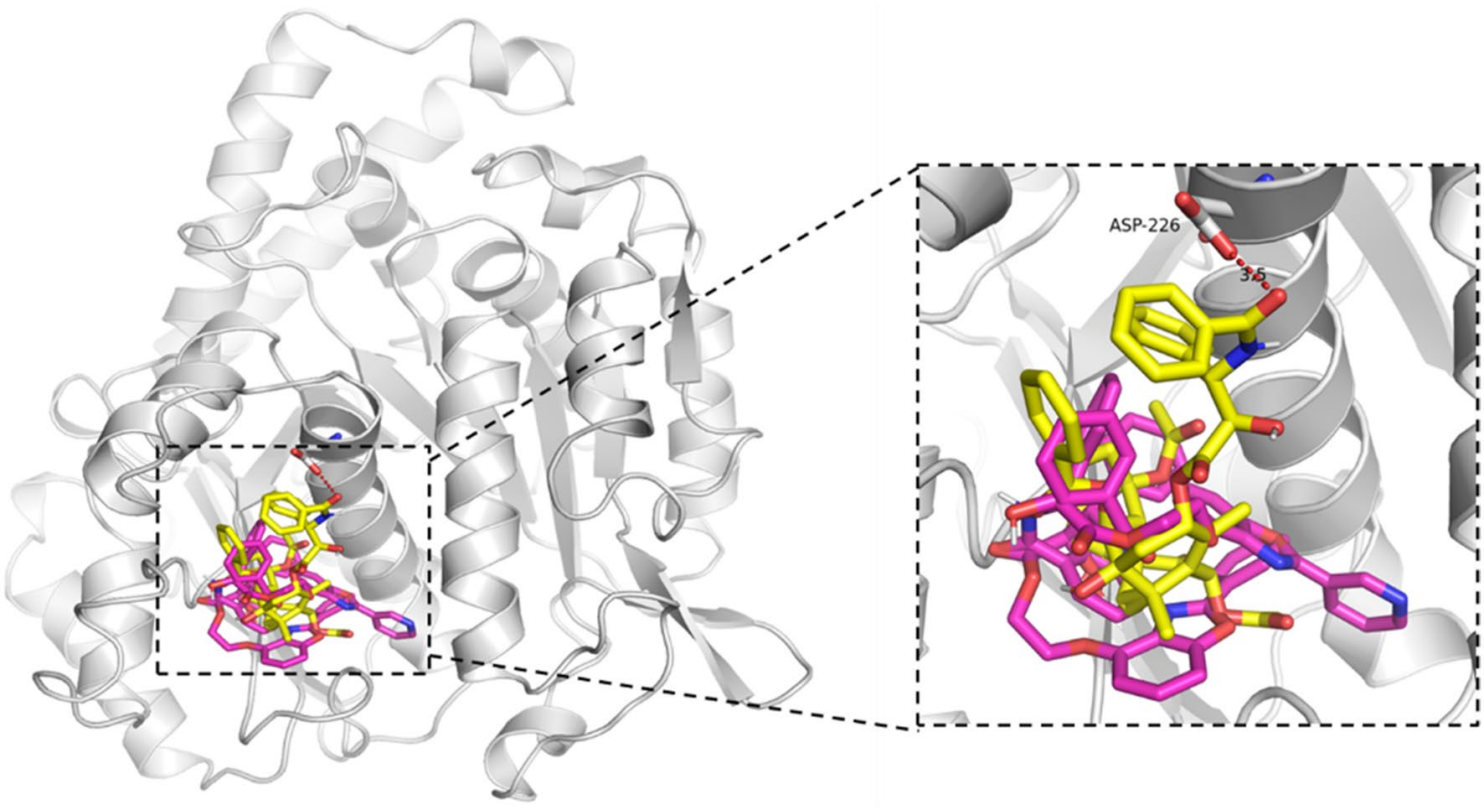
An overlay of the structures for compounds **7a**, **7g** and paclitaxel. *Compounds **7a** and **7g** in purple carbon atoms; paclitaxel in yellow carbon atoms.

An overlay of the structures for compounds **7a, 7g** and positive control paclitaxel was shown in Fig. 2. These three molecules embedded in 6I2I cavity and the binding region of compounds **7a** and **7g** was consistent with that of paclitaxel. The oxygen atom of the ester carbonyl in paclitaxel established the hydrogen bond interaction with residue Asp 226. However, Asp 226 didn’t interact with the other compounds, which could be caused by the greatly variation of structures.

**Figure 3:**
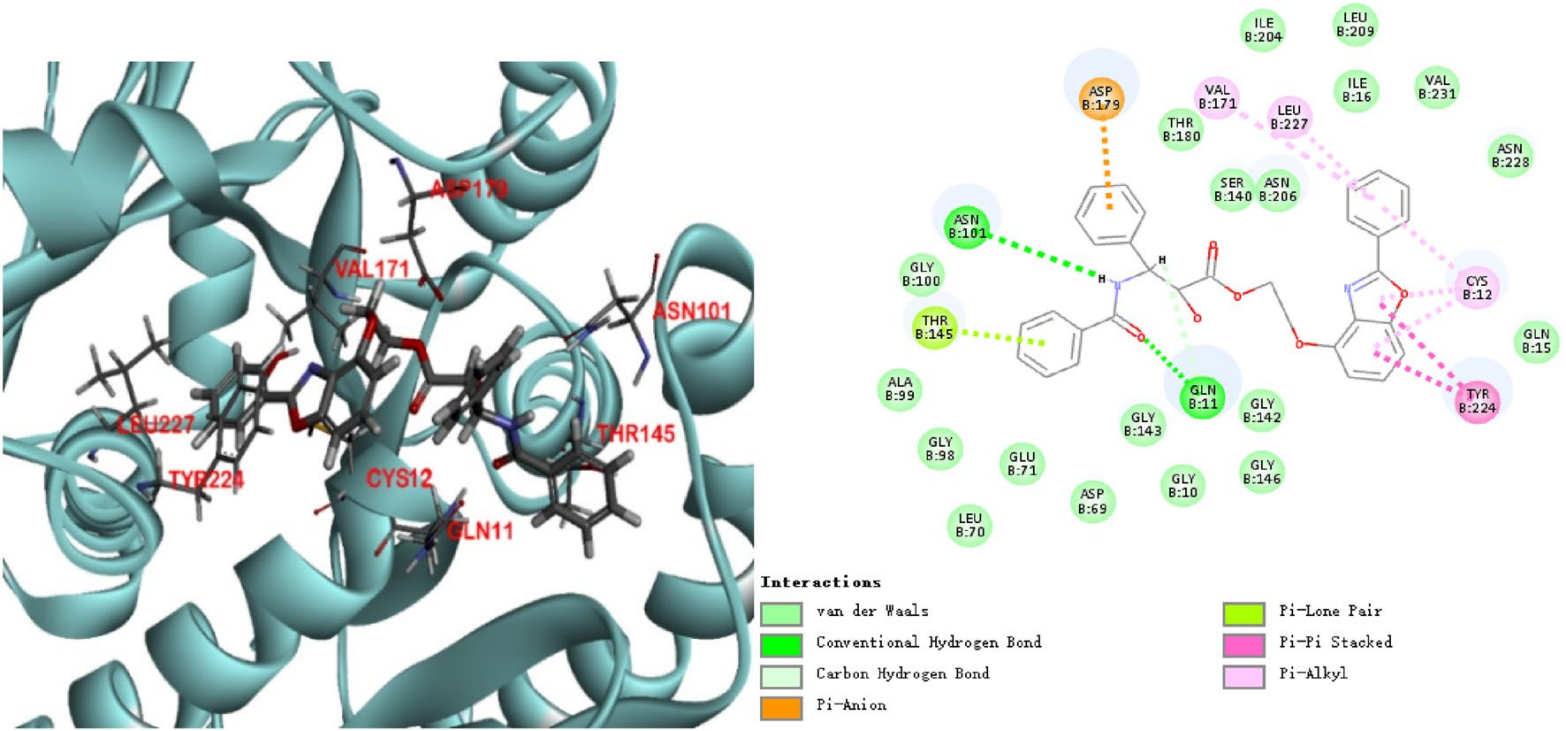
The molecular docking result and pattern of compound **7a** and beta-tubulin.

The binding model of **7a** and 6I2I protein was depicted in Fig. 3. The docking result showed that eight amino acids Asn 101, Thr 145, Gln 11, Asp 179, Tyr 224, Cys 12, Leu 227 and Val 171 located in the binding pocket of protein played vital roles in the combination with compound **7a**. The Pi-Pi stacking and Pi-alkyl bonds were formed between benzoxazoles of docking molecule and Tyr 224, Cys 12, respectively. The other three Pi-alkyl bonds were formed between the benzene ring locating at benzoxazoles and Cys 12, Leu 227 and Val 171. The portion of side chains deriving from paclitaxel made the hydrogen bonds with Asn 101 and Gln 11. On the other hand, a Pi-anion bond and van der waals as well formed between unique side chains and Asp 179, Thr 145, respectively.

**Figure 4:**
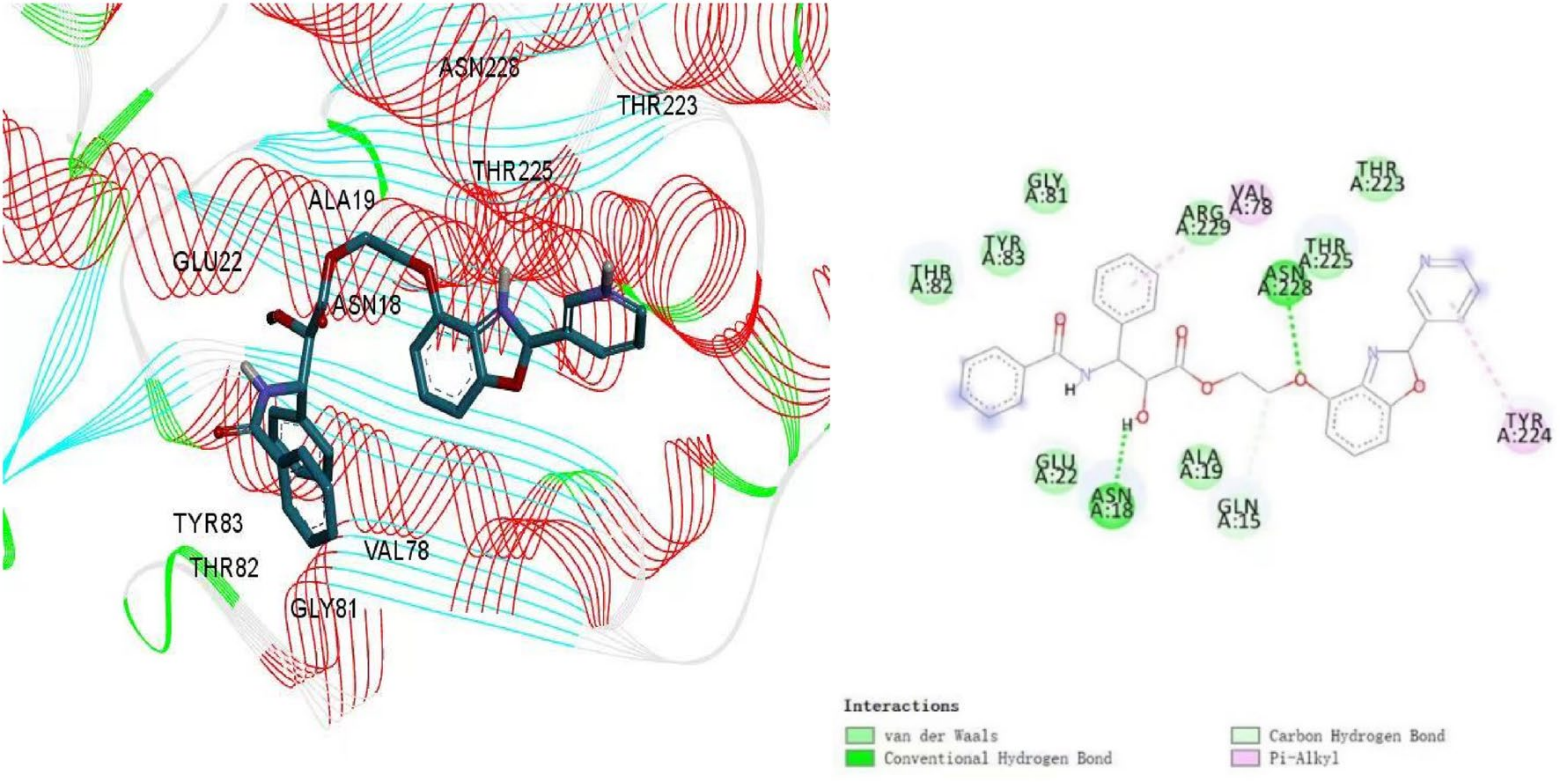
The molecular docking result and pattern of compound **7g** and beta-tubulin.

The molecular interactions for compound **7g** within the 6I2I active site was showed in Fig. 4. The docking results suggested that **7g** made hydrogen bonds with two amino acids Asn 18 and Asn 228. Besides, the molecule established the carbon hydrogen bonds interactions with amino acids Gln 15. Pyridine ring and one of benzene ring established Pi-alkyl bonds with Val 78 and Tyr 224. The above molecular docking result in molecular level foundation revealed that the paclitaxel-benzoxazoles hybrids could inhibit the cancer cells proliferation. Therefore, a series of paclitaxel-benzoxazoles hybrids were designed and synthesized using palladium-catalyzed direct coupling reaction to explore the potential inhibitors as therapy for cancer.

### Synthetic chemistry

**Scheme 1:**
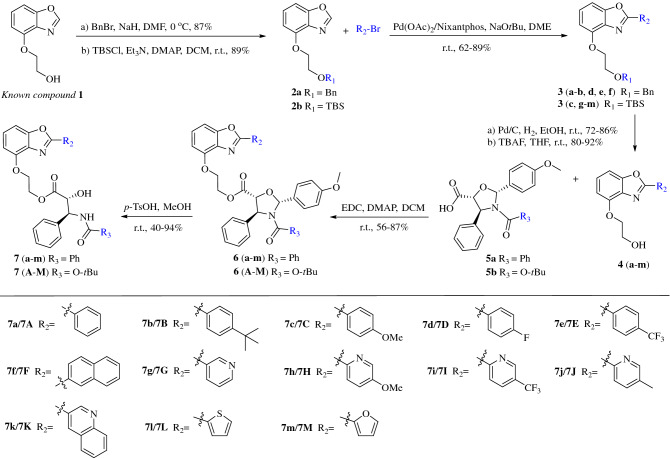
Synthesis of the paclitaxel-benzoxazoles hybrids **7a**–**7m** and **7A**–**7M.**

The synthetic route to target paclitaxel-benzoxazoles hybrids **7a**–**7m** and **7A**–**7M** were illustrated in Scheme 1. The first task was to prepare the key benzoxazoles intermediates **3a**–**3m**. Starting from the known compound **1**, masking of the free hydroxy group as a benzyl ether or silyl ether provided precursors **2a** or **2b** in 87% or 89% yield, respectively. The key intermediates **3a**–**3m** were obtained by palladium-catalyzed direct Csp^2^–H arylation of benzoxazoles **2a** or **2b** with different aryl-bromides. The coupling reaction was carried out under the catalytic system of Pd(OAc)_2_/Nixantphos/NaO*t*Bu in DME at room temperature and furnished the desired compounds **3a**–**3m** in 62–89% yields. Removement of benzyl ether or silyl ether of the benzoxazoles intermediates **3a**–**3m** resulted in the formation of benzoxazoles **4a**–**4m** with free hydroxy group in 72–92% yields. Next, we focused on synthesizing the paclitaxel-benzoxazoles hybrids. Esterification of **4a**–**4m** with purchased oxazolidinecarboxylic acids **5a** or **5b** resulted in the formation of oxazolidinecarboxylate **6a**–**6m** and **6A**–**6M** (Scheme 1). Treatment of the intermediates **6a**–**6m** and **6A**–**6M** with *p*-TsOH yielded twenty-six paclitaxel-benzoxazoles hybrids **7a**–**7m** and **7A**–**7M** in 40–94% yields.

### Inhibitory effects on the proliferation of MDA-MB-231 and HepG2 for paclitaxel-benzoxazoles hybrids

At the beginning of the assessment, the inhibition of the synthesized paclitaxel-benzoxazoles hybrids (**7a**–**7m** and **7A**–**7M**) on human breast cancer cells (MDA-MB-231) and liver hepatocellular cells (HepG2) was evaluated by MTT methods using paclitaxel as a control. The results revealed that most derivatives exhibited favorable antiproliferative activities against MDA-MB-231 and HepG2 cells at a concentration of 50 µM (Table 1). As shown in Table 1, Compounds **7a**–**7e**, **7i**, **7k**, **7l**, **7A**, **7B**, **7D** and **7E** showed better activities than positive control paclitaxel against both MDA-MB-231 and HepG2 cell lines at the high concentration. Among of them, compounds **7a**, **7h**, **7k**, **7B**, **7H** and **7I** showed antiproliferative effect of cell growth of MDA-MB-231 over 80%. Compounds **7a**, **7d**, **7h**, **7k**, **7B**, **7E** and **7I** as well displayed superior inhibitory activity on HepG2 cells. Particularly, compared with paclitaxel (inhibition ratio of 27.4% and 69.8%, respectively), compound **7k** showed a better inhibition ratio with 93.2% and 94.5% against MDA-MB-231 and HepG2, respectively. Furthermore, hybrids **7h** and **7B** exhibited better activities against HepG2 cells (97.1% and 92.3%, respectively) than MDA-MB-231 cells (82.1% and 83.7%, respectively).

**Table 1:** Antiproliferative ratio of paclitaxel-benzoxazoles hybrids **7a**–**7m** and **7A**–**7M** against two cancer cell lines at 50 µM.

Compd.	Antiproliferative ratio on MDA-MB-231 (%)	Antiproliferative ratio on HepG2 (%)	Compd.	Antiproliferative ratio on MDA-MB-231 (%)	Antiproliferative ratio on HepG2 (%)
**7a**	81.6	80.2	**7A**	73.7	71.6
**7b**	70.9	71.5	**7B**	83.7	92.3
**7c**	59.7	79.1	**7C**	38.4	50.2
**7d**	71.3	80.1	**7D**	77.1	75.8
**7e**	78.5	79.9	**7E**	65.9	84.0
**7f.**	66.7	58.8	**7F**	70.9	57.1
**7g**	13.0	8.0	**7G**	76.9	14.0
**7 h**	82.1	97.1	**7H**	85.4	5.8
**7i**	54.3	77.3	**7I**	82.1	5.7
**7j**	19.4	0.6	**7J**	70.7	27.2
**7k**	93.2	94.5	**7k**	41.0	68.1
**7l**	63.2	81.0	**7L**	42.1	56.9
**7m**	23.5	40.6	**7M**	29.5	70.6
Paclitaxel	27.4	69.8			

**Figure 5:**
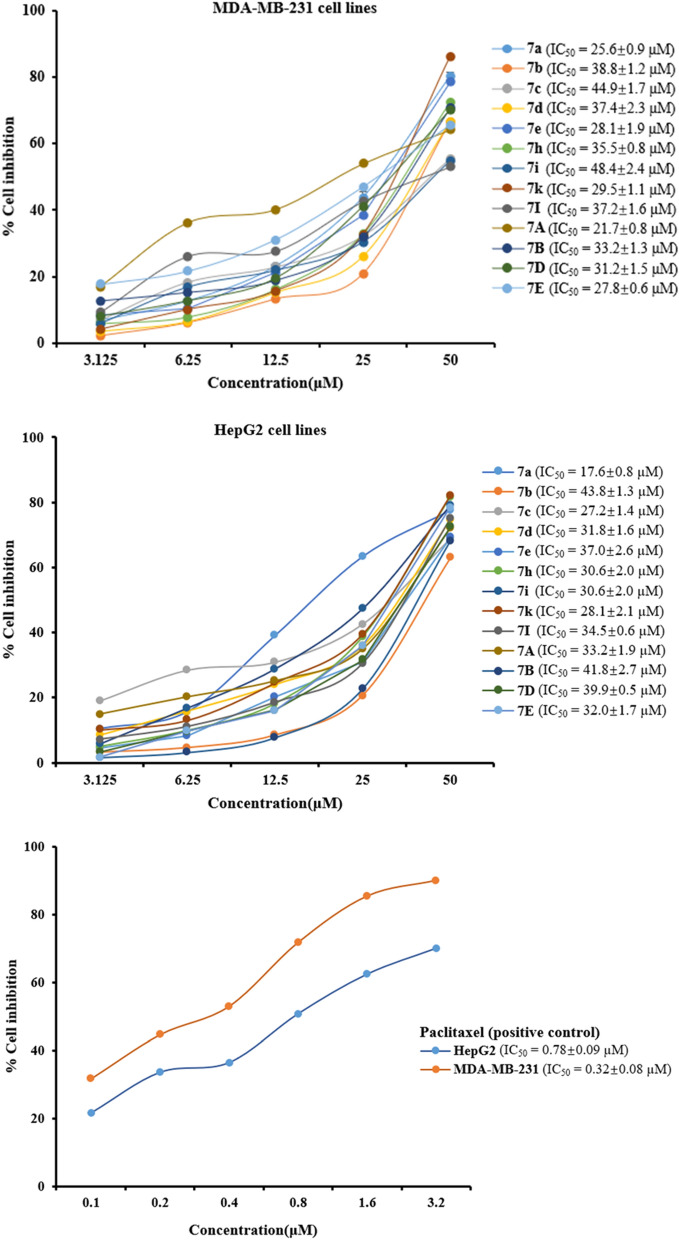
Dose–response analysis of cell growth inhibition activity against MDA-MB-231 and HepG2.

According to the desirable effect of above synthetics on the 2 cancer cell lines, the compounds with antiproliferative activity over 70% at 50 µM were tested for their half maximal inhibitory concentration (IC_50_) (Fig. 5). The potential inhibitory activities expressed as IC_50_ values for all compounds were shown in Tables 2 and 3. It is regrettable that the IC_50_ value of all hybrids both against MDA-MB-231 and HepG2 were equally much higher than those of paclitaxel (IC_50_ values of 0.32 ± 0.08 and 0.78 ± 0.09 μM, respectively). According to the results, it is supposed that the antiproliferative activities of synthesized hybrids had dose-dependent effect with concentration. Nonetheless, IC_50_ values of compound **7A** against MDA-MB-231 cells was 21.7 ± 0.8 µM, better than that of the others. And compounds **7a**, **7e**, **7k** and **7E** (IC_50_ values of 25.6 ± 0.9, 28.1 ± 1.9, 29.5 ± 1.1 and 27.8 ± 0.6 μM, respectively) exhibited moderate anticancer activity on MDA-MB-231. On the other hand, compounds **7c**, **7i** and **7k** possessed the IC_50_ values of 27.2 ± 1.4, 23.6 ± 1.4 and 28.1 ± 2.1 μM, respectively to exhibit anti-proliferative activities on HepG2. Hybrid **7a** (IC_50_ = 17.6 ± 0.8 μM) had good cytotoxic activities compared to other derivates.

**Table 2:** Antiproliferative activities of selected paclitaxel-benzoxazoles hybrids against MDA-MB-231.

Compd.	Percentage of antiproliferation against MDA-MB-231					IC_50_ (µM)
	3.125 µM	6.25 µM	12.5 µM	25 µM	50 µM	
**7a**	6.8 ± 0.6	12.7 ± 0.3	23.1 ± 0.8	43.7 ± 0.5	80.2 ± 0.8	25.6 ± 0.9
**7b**	2.2 ± 0.4	6.1 ± 0.8	13.2 ± 0.4	20.7 ± 0.5	66.4 ± 0.6	38.8 ± 1.2
**7c**	6.9 ± 1.4	18.3 ± 1.0	22.9 ± 0.9	32.1 ± 0.5	55.4 ± 0.6	44.9 ± 1.7
**7d**	3.4 ± 0.4	6.3 ± 1.1	15.0 ± 0.6	25.8 ± 0.7	66.5 ± 0.5	37.4 ± 2.3
**7e**	8.4 ± 0.7	10.3 ± 0.9	21.2 ± 1.4	38.3 ± 2.3	78.4 ± 1.8	28.1 ± 1.9
**7h**	5.9 ± 0.8	7.8 ± 0.5	16.0 ± 0.6	32.6 ± 0.4	72.7 ± 0.5	35.5 ± 0.8
**7i**	5.8 ± 1.5	16.8 ± 1.0	21.8 ± 1.8	30.3 ± 1.9	54.8 ± 0.8	48.4 ± 2.4
**7k**	4.1 ± 0.6	10.0 ± 0.9	15.5 ± 0.6	32.5 ± 1.3	86.0 ± 1.0	29.5 ± 1.1
**7l**	15.7 ± 1.1	19.1 ± 0.4	30.5 ± 2.1	41.5 ± 1.2	56.3 ± 1.9	37.2 ± 1.6
**7A**	16.8 ± 0.9	18.1 ± 2.3	20.0 ± 1.8	62.5 ± 0.3	75.0 ± 2.1	21.7 ± 0.8
**7B**	12.5 ± 0.7	15.1 ± 1.2	18.6 ± 1.8	31.7 ± 1.5	70.5 ± 1.7	33.2 ± 1.3
**7D**	8.0 ± 1.1	12.6 ± 0.7	19.3 ± 1.4	41.0 ± 2.0	70.2 ± 0.9	31.2 ± 1.5
**7E**	17.7 ± 1.2	21.6 ± 0.5	31.0 ± 0.4	46.8 ± 0.5	65.4 ± 0.8	27.8 ± 0.6
Paclitaxel	–	–	–	–	–	0.32 ± 0.08

**Table 3:** Antiproliferative activities of selected paclitaxel-benzoxazoles hybrids against HepG2.

Compd.	Percentage of antiproliferation against HepG2					IC_50_ (µM)
	3.125 µM	6.25 µM	12.5 µM	25 µM	50 µM	
**7a**	10.5 ± 0.7	15.7 ± 0.9	39.1 ± 0.3	63.3 ± 1.1	77.5 ± 0.6	17.6 ± 0.8
**7b**	3.2 ± 1.5	4.6 ± 1.1	8.5 ± 1.7	20.7 ± 1.0	62.9 ± 0.8	43.8 ± 1.3
**7c**	18.9 ± 0.6	28.3 ± 0.9	30.7 ± 1.8	42.4 ± 0.9	68.8 ± 1.1	27.2 ± 1.4
**7d**	8.4 ± 1.2	15.8 ± 1.5	23.9 ± 0.9	35.9 ± 0.8	74.6 ± 0.5	31.8 ± 1.6
**7e**	4.4 ± 1.3	8.3 ± 0.4	20.2 ± 0.5	30.9 ± 1.8	69.1 ± 1.2	37.0 ± 2.6
**7h**	4.9 ± 0.7	9.8 ± 0.8	18.1 ± 1.5	38.7 ± 1.6	81.7 ± 0.9	30.6 ± 2.0
**7i**	5.8 ± 2.1	16.9 ± 1.9	28.9 ± 1.7	47.3 ± 0.3	79.0 ± 0.6	23.6 ± 1.4
**7k**	10.1 ± 0.4	13.0 ± 0.7	24.5 ± 0.9	39.5 ± 1.5	82.0 ± 1.8	28.1 ± 2.1
**7l**	7.1 ± 2.6	11.1 ± 1.6	18.5 ± 0.4	30.5 ± 0.6	75.0 ± 0.9	34.5 ± 0.6
**7A**	14.9 ± 1.3	20.2 ± 1.5	25.1 ± 0.6	34.9 ± 1.4	71.9 ± 1.6	33.2 ± 1.9
**7B**	1.6 ± 1.9	3.9 ± 2.4	7.7 ± 2.5	22.7 ± 2.3	68.2 ± 1.7	41.8 ± 2.7
**7D**	3.3 ± 1.2	9.6 ± 0.9	16.2 ± 1.0	31.5 ± 0.5	72.4 ± 0.7	39.9 ± 0.5
**7E**	1.7 ± 1.4	9.65 ± 1.2	16.1 ± 2.1	42.8 ± 0.7	78.3 ± 1.1	32.0 ± 1.7
Paclitaxel	–	–	–	–	–	0.78 ± 0.09

## Conclusion

In summary, twenty-six new paclitaxel-benzoxazoles hybrids bearing only a C-13 side chain of paclitaxel were designed on both the SAR of paclitaxel and the molecular docking study. The newly hybrids were rapidly prepared through five simple reactions. The key synthetic strategy for the aryl-benzoxazoles moieties in the hybrids was palladium-catalyzed direct Csp^2^–H arylation of benzoxazoles with different aryl-bromides. At the concentration of 50 µM, most compounds showed moderate to good antiproliferative activity against human breast cancer cells (MDA-MB-231) and liver hepatocellular cells (HepG2). Some of the compounds (**7a**–**7e**, **7i**, **7k**, **7l**, **7A**, **7B**, **7D** and **7E**) at this concentration exhibited stronger activity against the two cell lines than paclitaxel. Unfortunately, the IC_50_ value of potential inhibitor were higher than those of paclitaxel. It is supposed that synthesized hybrids exhibited antiproliferative activities in a dose-dependent manner. Among of them, the optimal compound **7a** showed the best therapeutic potential to inhibit HepG2 cell growth with the IC_50_ values of 17.6 ± 0.8 µM. Owing to the remarkable cytotoxicity, **7a** needs an in-depth investigation to improve in terms of reducing dose-dependent effect, which might assist in the development of anticancer agents in the future. The results suggests that the use of baccatin-free hybrid component might be an effective strategy to establish paclitaxel-based hybrid library to find lead compounds against cancer.

## Experimental

### General experimental procedures

^1^H NMR spectra were recorded on a Bruker AV 400 or 600 nuclear magnetic resonance instrument (400 or 600 MHz). Chemical shifts were recorded in ppm relative to tetramethylsilane as the internal standard. ^13^C NMR data were collected on a Bruker AV 400 or 600 nuclear magnetic resonance instrument (100 or 150 MHz) with complete proton decoupling. Chemical shifts are reported in ppm with tetramethylsilane as the internal standard. HRESIMS were determined using a Waters Acquity UPLC/Xevo G2-S QTof mass spectrometer. Thin-layer chromatography silica gel GF254 plates and silica gel G (200–400 mesh) for column chromatography were purchased from Qingdao Ocean Chemical Plant (Qingdao, People's Republic of China). Unless otherwise specified, the reagents and solvents used in this work were all commercially available analytical or chemical grades, and used directly without any purification.

### Molecular docking study

The crystallographic structures of tubulin (PDB ID: 6I2I) was chosen as the template for the modeling study of compounds. The pdb file about the crystal structure of refined 13pf Hela Cell tubulin microtubule (6I2I.pdb) was obtained from the Protein Date Bank at http://www.rcsb.org/. The structures of compound were drawn by ChemBioDraw software and converted to mol file. The ligands and bound water were removed from the protein and the polar hydrogen was added. The energy minimized structures of tubulin (6I2I.pdb) and the ligand were generated by using prepare protein module and prepare ligand module, respectively. The whole tubulin complex was defined as a receptor. The molecular docking procedure was performed by using CDOCKER protocol for receptor-ligand interactions section of Discovery Studio Client software. Then, the lowest energy configuration of docking molecule was docked into 20 different binding sites of the prepared protein molecule using the standard parameters of Discovery Studio throughout the simulation. The preferred site was determined calculating binding energy values, and types of interactions of the docked protein with ligand were analyzed after the end of molecular docking.

### Preparation of compound 2a

To a solution of the known compound **1** (1.00 g, 5.58 mmol, 1.0 eq.) in dry DMF (20 mL) was added NaH (201 mg, 8.37Mmol, 1.5 eq.) at 0 °C. After 10 min, a solution of BnBr (0.80 mL, 6.70 mmol, 1.2 eq.) in DMF (5 mL) was added to the mixture dropwise. The whole mixture was stirred at 0 °C for 5 h, then quenched with saturated aqueous NaHCO_3_. After extraction with EtOAc (20 mL × 3), the combined organic layers were washed with H_2_O (20 mL × 3) and brine (30 mL), dried over Na_2_SO_4_, filtered and concentrated to dryness. Purification of the crude product via chromatography on silica gel (petroleum ether: EtOAc = 10:1) afforded compound **2a** (1.31 g, 87% yield) as yellow oil.

### Preparation of compound 2b

The known compound **1** (1.00 g, 5.58 mmol, 1.0 eq.) was dissolved in DCM (20 mL). Et_3_N (2.33mL, 16.74mmol, 3.0 eq.), TBSCl (1.56ML, 8.37Mmol, 1.5 eq.) and DMAP (341 mg, 2.79 mmol, 0.5 eq.) were added to the solution at ambient temperature. The reaction mixture was stirred at ambient temperature for 1.5 h, then quenched by H_2_O (10 mL). The layers were separated and the aqueous phase was extracted with DCM (5 mL × 2). The combined organic layers were washed with H_2_O (15 mL) and brine (10 mL), dried over Na_2_SO_4_, filtered, concentrated under reduced pressure led to a crude product. Flash column chromatography (petroleum ether: EtOAc = 50:1) gave the desired product **2b** (1.46 g, 89% yield) as colorless oil.

### General procedure for the synthesis of compounds 3a–**3**m

The mixture of Pd(OAc)_2_ (5 mol %) and NiXantPhos (7.5 mol %) in anhydrous DME (2.0 mL) was stirred at 25 °C under an argon atmosphere for 4 h to be a dark brown solution. Then, the dark brown solution was added to the compound **2a** or **2b** (0.50 mmol), arylbromides (0.60 mmol) and NaO*t*Bu (1.20 mmol) in in anhydrous DME (1.0 mL) dropwise via syringe. The reaction mixture was stirred for 12 h at 25 °C under an argon atmosphere. Then, the reaction mixture was quenched by some drops of H_2_O, diluted with EtOAc (3.0 mL), dried over MgSO_4_, and filtered over a pad celite. The filtrate was concentrated in vacuo. The crude residue was purified by silica gel column chromatography (petroleum ether/EtOAc) to obtain the desired compounds **3a**−**3m** as colorless oil, yield 62 − 89%.

Compound **3a**, colorless oil; yield 72%; ^1^H NMR (400 MHz, CDCl_3_) *δ* 8.30 (2H, d, *J* = 8.3 Hz), 7.54–7.48 (3H, m), 7.42–7.32 (4H, m), 7.31 (1H, t, *J* = 8.0 Hz), 7.26–7.19 (2H, m), 6.88 (1H, d, *J* = 8.0 Hz), 4.68 (2H, s), 4.57–4.55 (2H, m), 3.99–3.96 (2H, m); HRESIMS (*m/z*): calcd. for C_22_H_20_NO_3_, 346.1443; found 346.1421 [M + H]^+^.

Compound **3b**, colorless oil; yield 79%; ^1^H NMR (400 MHz, CDCl_3_) *δ* 8.25 (2H, d, *J* = 8.3 Hz), 7.55–7.49 (2H, m), 7.42–7.28 (5H, m), 7.25–7.16 (2H, m), 6.87 (1H, d, *J* = 8.0 Hz), 4.67 (2H, s), 4.56–4.54 (2H, m), 4.01–3.94 (2H, m), 1.37 (9H, s); HRESIMS (*m/z*): calcd. for C_26_H_28_NO_3_, 402.2069; found 406.2058 [M + H]^+^.

Compound **3c**, colorless oil; yield 84%; ^1^H NMR (400 MHz, CDCl_3_) *δ* 8.21 (2H, d, *J* = 9.0 Hz), 7.24–7.14 (2H, m), 7.04–6.97 (2H, m), 6.86 (1H, dd, *J* = 7.9, 0.9 Hz), 4.40 (2H, t, *J* = 5.8 Hz), 4.10 (2H, t, *J* = 5.8 Hz), 3.88 (3H, s), 0.91 (9H, s), 0.12 (6H, s); HRESIMS (*m/z*): calcd. for C_22_H_30_NO_4_Si, 400.1944; found 400.1965 [M + H]^+^.

Compound **3d**, colorless oil; yield 62%; ^1^H NMR (400 MHz, CDCl_3_) *δ* 8.23 (2H, d, *J* = 8.2 Hz), 7.41–7.38 (2H, m), 7.37–7.33 (2H, m), 7.31 (1H, d, *J* = 8.0 Hz), 7.26–7.17 (4H, m), 6.87 (1H, d, *J* = 8.0 Hz), 4.67 (2H, s), 4.56–4.52 (2H, m), 4.00–3.95 (2H, m); HRESIMS (*m/z*): calcd. for C_22_H_18_NO_3_F, 364.1349; found 364.1326 [M + H]^+^.

Compound **3e**, colorless oil; yield 68%; ^1^H NMR (400 MHz, CDCl_3_) *δ* 8.39 (2H, d, *J* = 8.3 Hz), 7.77 (2H, d, *J* = 8.4 Hz), 7.41–7.28 (6H, m), 7.23 (1H, d, *J* = 8.0 Hz), 6.89 (1H, d, *J* = 8.0 Hz), 4.68 (2H, s), 4.57–4.53 (2H, m), 3.99–3.97 (2H, m); HRESIMS (*m/z*): calcd. for C_23_H_19_NO_3_F_3_, 414.1317; found 414.1329 [M + H]^+^.

Compound **3f**, colorless oil; yield 74%; ^1^H NMR (400 MHz, CDCl_3_) *δ* 8.79 (1H, s), 8.35 (1H, dd, *J* = 8.6, 1.7 Hz), 7.97 (2H, t, *J* = 9.1 Hz), 7.92–7.87 (1H, m), 7.60–7.54 (2H, m), 7.42–7.33 (4H, m), 7.32–7.27 (2H, m), 7.26–7.23 (1H, m), 6.89 (1H, dd, *J* = 7.5, 1.5 Hz), 4.69 (2H, s), 4.60–4.56 (2H, m), 4.02–3.98 (2H, m); HRESIMS (*m/z*): calcd. for C_26_H_22_NO_3_, 396.1600; found 396.1614 [M + H]^+^.

Compound **3g**, colorless oil; yield 81%; ^1^H NMR (400 MHz, CDCl_3_) *δ* 9.47 (1H, d, *J* = 1.6 Hz), 8.74 (1H, dd, *J* = 4.8, 1.6 Hz), 8.52 (1H, dt, *J* = 8.0, 1.9 Hz), 7.45 (1H, ddd, *J* = 8.0, 4.9, 0.6 Hz), 7.29 (1H, t, *J* = 8.1 Hz), 7.23–7.20 (1H, m), 6.90 (1H, d, *J* = 7.8 Hz), 4.42 (2H, t, *J* = 5.6 Hz), 4.11 (2H, t, *J* = 5.6 Hz,), 0.91 (9H, s), 0.12 (6H, s); HRESIMS (*m/z*): calcd. for C_20_H_27_N_2_O_3_Si, 371.1791; found 371.1804 [M + H]^+^.

Compound **3h**, colorless oil; yield 89%; ^1^H NMR (400 MHz, CDCl_3_) *δ* 8.46 (1H, d, *J* = 2.7 Hz), 8.38 (1H, d, *J* = 8.7 Hz), 7.34 (1H, dd, *J* = 8.8, 2.9 Hz), 7.28 (1H, d, *J* = 8.2 Hz), 7.25 (1H, d, *J* = 6.1 Hz), 6.88 (1H, dd, *J* = 6.7, 2.3 Hz), 4.41 (2H, t, *J* = 5.7 Hz), 4.10 (2H, t, *J* = 5.7 Hz), 3.95 (3H, s), 0.91 (9H, s), 0.11 (6H, s); HRESIMS (*m/z*): calcd. for C_21_H_29_N_2_O_4_Si, 401.1897; found 401.1882 [M + H]^+^.

Compound **3i**, colorless oil; yield 69%; ^1^H NMR (400 MHz, CDCl_3_) *δ* 9.04 (1H, s), 8.56 (1H, d, *J* = 8.3 Hz), 8.12 (1H, dd, *J* = 8.3, 2.0 Hz), 7.36 (1H, t, *J* = 8.1 Hz), 7.30 (1H, d, *J* = 7.7 Hz), 6.93 (1H, d, *J* = 7.9 Hz), 4.43 (2H, t, *J* = 5.6 Hz), 4.11 (2H, t, *J* = 5.6 Hz), 0.91 (9H, s), 0.11 (6H, s); HRESIMS (*m/z*): calcd. for C_21_H_26_N_2_O_3_F_3_Si, 439.1665; found 439.1682 [M + H]^+^.

Compound **3j**, colorless oil; yield 73%; ^1^H NMR (400 MHz, CDCl_3_) *δ* 8.64 (1H, d, *J* = 5.0 Hz), 8.29 (1H, s), 7.33–7.28 (2H, m), 7.24 (1H, s), 6.90 (1H, dd, *J* = 7.4, 1.5 Hz), 4.43 (2H, t, *J* = 5.8 Hz), 4.10 (2H, t, *J* = 5.8 Hz), 2.47 (3H, s), 0.91 (9H, s), 0.11 (6H, s); HRESIMS (*m/z*): calcd. for C_21_H_29_N_2_O_3_Si, 385.1947; found 385.1958 [M + H]^+^.

Compound **3k**, colorless oil; yield 81%; ^1^H NMR (400 MHz, CDCl_3_) *δ* 9.76 (1H, d, *J* = 2.1 Hz), 9.01 (1H, d, *J* = 1.9 Hz), 8.18 (1H, d, *J* = 8.4 Hz), 7.96 (1H, d, *J* = 7.9 Hz), 7.84–7.79 (1H, m), 7.66–7.62 (1H, m), 7.31 (1H, t, *J* = 8.1 Hz), 7.25–7.18 (1H, m), 6.93 (1H, d, *J* = 8.0 Hz), 4.44 (2H, t, *J* = 5.7 Hz), 4.13 (2H, t, *J* = 5.7 Hz), 0.92 (9H, s), 0.13 (6H, s); HRESIMS (*m/z*): calcd. for C_24_H_28_N_2_O_3_Si, 420.1869; found 420.1875 [M + H]^+^.

Compound **3l**, colorless oil; yield 84%; ^1^H NMR (400 MHz, CDCl_3_) *δ* 7.91 (1H, dd, *J* = 3.7, 1.2 Hz), 7.52 (1H, dd, *J* = 5.0, 1.2 Hz), 7.23 (1H, t, *J* = 8.1 Hz), 7.18–7.13 (2H, m), 6.87 (1H, dd, *J* = 8.1, 0.6 Hz), 4.40 (2H, t, *J* = 5.8 Hz), 4.09 (2H, t, *J* = 5.8 Hz), 0.91 (9H, s), 0.11 (6H, s); HRESIMS (*m/z*): calcd. for C_19_H_26_NO_3_SSi, 376.1403; found 376.1423 [M + H]^+^.

Compound **3m**, colorless oil; yield 79%; ^1^H NMR (400 MHz, CDCl_3_) *δ* 7.64 (1H, dd, *J* = 1.7, 0.7 Hz), 7.28 (1H, dd, *J* = 3.6, 0.7 Hz), 7.24 (1H, d, *J* = 8.1 Hz), 7.17 (1H, dd, *J* = 8.2, 0.8 Hz), 6.88 (1H, dd, *J* = 8.1, 0.7 Hz), 6.60 (1H, dd, *J* = 3.5, 1.8 Hz), 4.40 (2H, t, *J* = 5.8 Hz), 4.08 (2H, t, *J* = 5.8 Hz), 0.90 (9H, s), 0.10 (6H, s); HRESIMS (*m/z*): calcd. for C_19_H_26_NO_4_Si, 360.1631; found 360.1646 [M + H]^+^.

### General procedure for the synthesis of compounds 4a, 4b and 4d-4f

A suspension of compound **4** (0.40 mmol) and Pd/C (10 mmol %) in EtOH (5.0 mL) was heated at ambient temperature under a hydrogen atmosphere for 5 h. The mixture was filtered through a pad of Celite®, which was rinsed with EtOH repeatedly. Concentration of the filtrate followed by flash column chromatography (petroleum ether/EtOAc) of the residue led to compounds **4a**, **4b**, and **4d**–**4f** as colorless oil, yield 72–86%.

Compound **4a**, colorless oil; yield 83%; ^1^H NMR (400 MHz, CDCl_3_) *δ* 8.27 (2H, d, *J* = 8.2 Hz), 7.54–7.48 (3H, m), 7.29–7.20 (2H, m), 6.87 (1H, d, *J* = 8.0 Hz), 4.43–4.41 (2H, m), 4.09–4.07 (2H, m); HRESIMS (*m/z*): calcd. for C_15_H_14_NO_3_, 256.0974; found 256.0983 [M + H]^+^.

Compound **4b**, colorless oil; yield 86%; ^1^H NMR (400 MHz, CDCl_3_) *δ* 8.17 (2H, d, *J* = 8.0 Hz), 7.54–7.50 (2H, m), 7.28–7.19 (2H, m), 6.85 (1H, d, *J* = 8.0 Hz), 4.43–4.34 (2H, m), 4.08–4.06 (2H, m), 3.66 (1H, s), 1.36 (9H, s); HRESIMS (*m/z*): calcd. for C_19_H_22_NO_3_, 312.1600; found 312.1609 [M + H]^+^.

Compound **4d**, colorless oil; yield 72%; ^1^H NMR (400 MHz, CDCl_3_) *δ* 8.32 (2H, d, *J* = 8.0 Hz), 7.27 (1H, t, *J* = 8.0 Hz), 7.25–7.14 (3H, m), 6.86 (1H, d, *J* = 7.9 Hz), 4.43–4.35 (2H, m), 4.09–4.04 (2H, m); HRESIMS (*m/z*): calcd. for C_15_H_13_NO_3_F, 274.0879; found 274.0862 [M + H]^+^.

Compound **4e**, colorless oil; yield 79%; ^1^H NMR (400 MHz, CDCl_3_) *δ* 8.35 (2H, d, *J* = 8.2 Hz), 7.76 (2H, d, *J* = 8.3 Hz), 7.32 (1H, t, *J* = 8.1 Hz), 7.24 (1H, d, *J* = 8.2 Hz), 6.88 (1H, d, *J* = 8.0 Hz), 4.47–4.39 (2H, m), 4.10 (2H, d, *J* = 6.9 Hz), 3.14 (1H, s); HRESIMS (*m/z*): calcd. for C_16_H_13_NO_3_F_3_, 324.0848; found 324.0857 [M + H]^+^.

Compound **4f**, colorless oil; yield 82%; ^1^H NMR (400 MHz, CDCl_3_) *δ* 8.75 (1H, s), 8.30 (1H, dd, *J* = 8.6, 1.7 Hz), 8.00–7.93 (2H, m), 7.89 (1H, dd, *J* = 6.1, 2.9 Hz), 7.62–7.53 (2H, m), 7.32–7.24 (2H, m), 6.87 (1H, dd, *J* = 7.4, 1.5 Hz), 4.47–4.41 (2H, m), 4.10 (2H, d, *J* = 3.6 Hz), 3.45 (1H, s); HRESIMS (*m/z*): calcd. for C_19_H_16_NO_3_, 306.1130; found 306.1149 [M + H]^+^.

### General procedure for the synthesis of compounds 4c and 4g-4m

To a solution of compound **4** (0.40 mmol) in THF (3.0 mL) was added TBAF/THF (0.5 mL, 1 mol/L). After stirring for 5 min to 1 h, the reaction was quenched with saturated aqueous NH_4_Cl and extracted with EtOAc. The combined organic extracts were washed with brine, dried over anhydrous Na_2_SO_4_, and concentrated to give a residue. Purification by flash chromatography on silica gel (petroleum ether/EtOAc) provided compounds **4c** and **4 g**–**4m** as colorless oil, yield 80–92%.

Compound **4c**, colorless oil; yield 92%; ^1^H NMR (400 MHz, CDCl_3_) *δ* 8.22–8.13 (2H, m), 7.25–7.17 (2H, m), 7.04–6.98 (2H, m), 6.84 (1H, dd, *J* = 7.5, 1.3 Hz), 4.42–4.36 (2H, m), 4.10–4.02 (2H, m), 3.89 (3H, s), 3.54 (1H, s); HRESIMS (*m/z*): calcd. for C_16_H_16_NO_4_, 286.1079; found 286.1083 [M + H]^+^.

Compound **4g**, colorless oil; yield 84%; ^1^H NMR (400 MHz, CDCl_3_) *δ* 9.45 (1H, d, *J* = 1.5 Hz), 8.75 (1H, dd, *J* = 4.8, 1.6 Hz), 8.51 (1H, dt, *J* = 8.0, 1.9 Hz), 7.45 (1H, ddd, *J* = 8.0, 4.9, 0.7 Hz), 7.32 (1H, t, *J* = 8.1 Hz), 7.24 (1H, d, *J* = 0.8 Hz), 6.88 (1H, d, *J* = 8.0 Hz), 4.42 (2H, d, *J* = 4.6 Hz), 4.09 (2H, d, *J* = 4.0 Hz), 3.06 (1H, s); HRESIMS (*m/z*): calcd. for C_14_H_13_N_2_O_3_, 257.0926; found 257.0941[M + H]^+^.

Compound **4h**, colorless oil; yield 85%; ^1^H NMR (400 MHz, CDCl_3_) *δ* 8.46 (1H, d, *J* = 2.8 Hz,), 8.30 (1H, d, *J* = 8.8 Hz), 7.34–7.27 (3H, m), 6.85 (1H, dd, *J* = 6.2, 2.8 Hz), 4.42–4.37 (2H, m), 4.07 (2H, d, *J* = 2.3 Hz), 3.94 (3H, s), 3.40 (1H, s); HRESIMS (*m/z*): calcd. for C_15_H_15_N_2_O_4_, 287.1032; found 287.1028 [M + H]^+^.

Compound **4i**, colorless oil; yield 69%; ^1^H NMR (400 MHz, CDCl_3_) *δ* 9.05 (1H, s), 8.50 (1H, d, *J* = 8.3 Hz), 8.12 (1H, dd, *J* = 8.3, 2.0 Hz), 7.38 (1H, t, *J* = 8.1 Hz), 7.32 (1H, d, *J* = 8.2 Hz), 6.90 (1H, d, *J* = 7.9 Hz), 4.44–4.40 (2H, m), 4.10 (2H, d, *J* = 3.9 Hz), 2.90 (1H, s); HRESIMS (*m/z*): calcd. for C_15_H_12_N_2_O_3_F_3_, 325.0800; found 325.0820 [M + H]^+^.

Compound **4j**, colorless oil; yield 80%; ^1^H NMR (400 MHz, CDCl_3_) *δ* 8.65 (1H, d, *J* = 5.0 Hz), 8.22 (1H, s), 7.31 (2H, dd, *J* = 10.7, 4.5 Hz), 7.29–7.23 (1H, m), 6.87 (1H, dd, *J* = 7.0, 2.0 Hz), 4.45–4.40 (2H, m), 4.11–4.06 (2H, m), 3.07 (1H, s), 2.47 (3H, s); HRESIMS (*m/z*): calcd. for C_15_H_15_N_2_O_3_, 271.1083; found 271.1075 [M + H]^+^.

Compound **4k**, colorless oil; yield 87%; ^1^H NMR (400 MHz, CDCl_3_) *δ* 9.70 (1H, d, *J* = 2.1 Hz), 8.97 (1H, d, *J* = 1.9 Hz), 8.18 (1H, d, *J* = 8.5 Hz), 7.95 (1H, d, *J* = 8.1 Hz), 7.84–7.79 (1H, m), 7.64 (1H, t, *J* = 7.5 Hz), 7.31 (1H, t, *J* = 8.1 Hz), 7.25 (1H, s), 6.89 (1H, d, *J* = 7.9 Hz), 4.48–4.43 (2H, m), 4.13 (2H, d, *J* = 3.0 Hz), 3.09 (1H, s); HRESIMS (*m/z*): calcd. for C_18_H_15_N_2_O_3_, 307.1083; found 307.1096 [M + H]^+^.

Compound **4l**, colorless oil; yield 84%; ^1^H NMR (400 MHz, CDCl_3_) *δ* 7.90 (1H, dd, *J* = 3.7, 1.2 Hz), 7.54 (1H, dd, *J* = 5.0, 1.2 Hz), 7.25 (2H, t, *J* = 8.1 Hz), 7.21–7.14 (2H, m), 6.84 (1H, dd, *J* = 8.0, 0.8 Hz), 4.41–4.36 (2H, m), 4.09–4.04 (2H, m), 3.46 (1H, s); HRESIMS (*m/z*): calcd. for C_13_H_12_NO_3_S, 262.0538; found 262.0547 [M + H]^+^.

Compound **4m**, colorless oil; yield 89%; ^1^H NMR (400 MHz, CDCl_3_) *δ* 7.66 (1H, d, *J* = 1.6 Hz), 7.27 (2H, dd, *J* = 10.0, 6.3 Hz), 7.20 (1H, d, *J* = 8.1 Hz), 6.85 (1H, d, *J* = 8.0 Hz), 6.61 (1H, dd, *J* = 3.5, 1.7 Hz), 4.39–4.35 (2H, m), 4.08–4.03 (2H, m), 3.27 (1H, s); HRESIMS (*m/z*): calcd. for C_13_H_12_NO_4_, 246.0766; found 246.0749 [M + H]^+^.

### General procedure for the synthesis of compounds 6a–6**m** and 6A–6M

A suspension of compound **4** (0.20 mmol), **5a** or **5b** (0.30 mmol), EDC (0.40 mmol) and DMAP (0.40 mmol) in DCM (5 mL) was stirred at ambient temperature for 5–14 h and then quenched with HCl (1 M). A saturated aqueous solution of NaHCO_3_ was added to the mixture to adjust the mixture to pH 7, then the mixture was extracted with EtOAc for three times. The organic layer was combined and treated with H_2_O and brine, then dried over Na_2_SO_4_. The residue was purified by silica gel column chromatography (petroleum ether/EtOAc) to afford the corresponding compound **6a**–**6m** and **6A**–**6M** as colorless oil, yield 56–87%.

Compound **6a**, colorless oil; yield 79%; ^1^H NMR (400 MHz, CDCl_3_) *δ* 8.23 (2H, d, *J* = 8.3 Hz), 7.53–7.49 (3H, m), 7.27 (4H, d, *J* = 1.5 Hz), 7.26–7.18 (7H, m), 7.10 (2H, t, *J* = 7.7 Hz), 6.96–6.76 (4H, m), 5.44 (1H, s), 4.92 (1H, s), 4.74–4.65 (4H, m), 3.79 (3H, s); HRESIMS (*m/z*): calcd. for C_39_H_33_N_2_O_7_, 641.2288; found 641.2263 [M + H]^+^.

Compound **6b**, colorless oil; yield 85%; ^1^H NMR (400 MHz, CDCl_3_) *δ* 8.17 (2H, d, *J* = 8.5 Hz), 7.51 (2H, d, *J* = 8.5 Hz), 7.50–7.33 (5H, m), 7.25–7.16 (7H, m), 7.11 (2H, t, *J* = 7.7 Hz), 6.85–6.78 (3H, m), 5.43 (1H, s), 4.92 (1H, s), 4.74–4.61 (4H, m), 3.80 (3H, s), 1.37 (9H, s); HRESIMS (*m/z*): calcd. for C_43_H_41_N_2_O_7_, 697.2914; found 697.2890 [M + H]^+^.

Compound **6c**, colorless oil; yield 77%; ^1^H NMR (400 MHz, CDCl_3_) *δ* 8.22 (2H, d, *J* = 8.2 Hz), 7.36–7.27 (5H, m), 7.23–7.20 (6H, m), 7.11 (2H, t, *J* = 7.7 Hz), 7.03–6.98 (2H, m), 6.97–6.68 (4H, m), 5.44 (1H, s), 4.91 (1H, s), 4.73–4.62 (4H, m), 3.89 (3H, s), 3.80 (3H, s); HRESIMS (*m/z*): calcd. for C_40_H_35_N_2_O_8_, 671.2393; found 671.2405 [M + H]^+^.

Compound **6d**, colorless oil; yield 87%; ^1^H NMR (400 MHz, CDCl_3_) *δ* 8.26 (2H, d, *J* = 8.2 Hz), 7.28–7.27 (5H, m), 7.25–7.20 (7H, m), 7.17 (2H, d, *J* = 8.7 Hz), 7.11 (2H, t, *J* = 7.7 Hz), 6.83–6.81 (3H, m), 5.43 (1H, s), 4.92 (1H, s), 4.72–4.69 (2H, m), 4.68–4.63 (2H, m), 3.80 (3H, s); HRESIMS (*m/z*): calcd. for C_39_H_32_FN_2_O_7_, 659.2194; found 659.2206 [M + H]^+^.

Compound **6e**, colorless oil; yield 78%; ^1^H NMR (400 MHz, CDCl_3_) *δ* 8.35 (2H, d, *J* = 8.1 Hz), 7.74 (2H, d, *J* = 8.2 Hz), 7.52–7.27 (7H, m), 7.24–7.23 (4H, m), 7.11 (2H, t, *J* = 7.6 Hz), 6.85 (2H, d, *J* = 7.8 Hz), 6.80 (2H, d, *J* = 7.0 Hz), 5.44 (1H, s), 4.92 (1H, s), 4.71 (2H, d, *J* = 4.0 Hz), 4.66 (2H, d, *J* = 4.4 Hz), 3.79 (3H, s); HRESIMS (*m/z*): calcd. for C_40_H_32_F_3_N_2_O_7_, 709.2162; found 709.2196 [M + H]^+^.

Compound **6f**, colorless oil; yield 66%; ^1^H NMR (400 MHz, CDCl_3_) *δ* 8.77 (1H, s), 8.32 (1H, dd, *J* = 8.6, 1.7 Hz), 7.99–7.88 (3H, m), 7.60–7.54 (2H, m), 7.44–7.27 (6H, m), 7.25–7.22 (5H, m), 7.11 (2H, t, *J* = 7.7 Hz), 6.95–6.75 (4H, m), 5.44 (1H, s), 4.93 (1H, s), 4.76–4.67 (4H, m), 3.77 (3H, s); HRESIMS (*m/z*): calcd. for C_43_H_35_N_2_O_7_, 691.2444; found 691.2417 [M + H]^+^.

Compound **6g**, colorless oil; yield 74%; ^1^H NMR (400 MHz, CDCl_3_) *δ* 9.45 (1H, d, *J* = 2.0 Hz), 8.75 (1H, dd, *J* = 4.9, 1.6 Hz), 8.50 (1H, dt, *J* = 8.0, 1.8 Hz), 7.43 (1H, dd, *J* = 8.0, 4.9 Hz), 7.31–7.27 (7H, m), 7.25–7.21 (4H, m), 7.12 (2H, t, *J* = 7.7 Hz), 6.94–6.77 (4H, m), 5.44 (1H, s), 4.91 (1H, s), 4.71–4.65 (4H, m), 3.80 (3H, s); HRESIMS (*m/z*): calcd. for C_38_H_32_N_3_O_7_, 642.2240; found 642.2213 [M + H]^+^.

Compound **6h**, colorless oil; yield 69%; ^1^H NMR (400 MHz, CDCl_3_) *δ* 8.46 (1H, d, *J* = 2.8 Hz), 8.32 (1H, d, *J* = 8.7 Hz), 7.53–7.26 (8H, m), 7.24–7.22 (4H, m), 7.11 (2H, t, *J* = 7.7 Hz), 7.01–6.71 (4H, m), 5.45 (1H, s), 4.91 (1H, s), 4.73–4.60 (4H, m), 3.94 (3H, s), 3.80 (3H, s); HRESIMS (*m/z*): calcd. for C_39_H_34_N_3_O_8_, 672.2346; found 672.2320 [M + H]^+^.

Compound **6i**, colorless oil; yield 66%; ^1^H NMR (400 MHz, CDCl_3_) *δ* 9.02 (1H, s), 8.49 (1H, d, *J* = 8.3 Hz), 8.04 (1H, dd, *J* = 8.3, 2.1 Hz), 7.39–7.32 (3H, m), 7.30–7.19 (10H, m), 7.12 (2H, t, *J* = 7.7 Hz), 6.88 (2H, dd, *J* = 6.9, 2.0 Hz), 6.79 (2H, d, *J* = 8.1 Hz), 5.46 (1H, s), 4.92 (1H, s), 4.75–4.70 (2H, m), 4.66 (2H, dd, *J* = 9.5, 4.5 Hz), 3.78 (3H, s); HRESIMS (*m/z*): calcd. for C_39_H_31_F_3_N_3_O_7_, 710.2114; found 710.2116 [M + H]^+^.

Compound **6j**, colorless oil; yield 83%; ^1^H NMR (400 MHz, CDCl_3_) *δ* 8.65 (1H, d, *J* = 5.0 Hz), 8.23 (1H, s), 7.30 (7H, m), 7.23 (5H, m), 7.11 (2H, t, *J* = 7.7 Hz), 6.99–6.72 (m, 4H), 5.44 (1H, s), 4.92 (1H, d, *J* = 4.3 Hz), 4.72–4.64 (4H, m), 3.79 (3H, s), 2.44 (3H, s); HRESIMS (*m/z*): calcd. for C_39_H_34_N_3_O_7_, 656.2397; found 656.2419 [M + H]^+^.

Compound **6k**, colorless oil; yield 70%; ^1^H NMR (400 MHz, CDCl_3_) *δ* 9.72 (1H, d, *J* = 2.1 Hz), 9.00 (1H, d, *J* = 1.9 Hz), 8.19 (1H, d, *J* = 8.4 Hz), 7.95 (1H, d, *J* = 7.9 Hz), 7.84–7.79 (1H, m), 7.63 (1H, t, *J* = 7.5 Hz), 7.42–7.27 (7H, m), 7.25–7.23 (4H, m), 7.12 (2H, t, *J* = 7.7 Hz), 6.87 (2H, dd, *J* = 6.4, 2.6 Hz), 6.79 (2H, d, *J* = 8.1 Hz), 5.45 (1H, s), 4.92 (1H, s), 4.74–4.68 (4H, m), 3.77 (3H, s); HRESIMS (*m/z*): calcd. for C_42_H_34_N_3_O_7_, 692.2397; found 692.2390 [M + H]^+^.

Compound **6l**, colorless oil; yield 72%; ^1^H NMR (400 MHz, CDCl_3_) *δ* 7.90 (1H, dd, *J* = 3.7, 1.1 Hz), 7.53 (1H, dd, *J* = 5.0, 1.1 Hz), 7.49–7.26 (6H, m), 7.25–7.19 (5H, m), 7.18–7.15 (1H, m), 7.12 (2H, t, *J* = 7.7 Hz), 7.00–6.74 (4H, m), 5.43 (1H, s), 4.91 (1H, s), 4.71–4.60 (4H, m), 3.80 (3H, s); HRESIMS (*m/z*): calcd. for C_37_H_31_N_2_O_7_S, 647.1852; found 647.1868 [M + H]^+^.

Compound **6m**, colorless oil; yield 56%; ^1^H NMR (400 MHz, CDCl_3_) *δ* 7.64 (1H, d,* J* = 1.0 Hz), 7.50–7.26 (7H, m), 7.25–7.19 (5H, m), 7.12 (2H, t, *J* = 7.7 Hz), 6.83–6.81 (4H, m), 6.60 (1H, dd, *J* = 3.5, 1.8 Hz), 5.42 (1H, s), 4.91 (1H, s), 4.72–4.57 (4H, m), 3.80 (3H, s); HRESIMS (*m/z*): calcd. for C_37_H_31_N_2_O_8_, 631.2080; found 631.2050 [M + H]^+^.

Compound **6A**, colorless oil; yield 80%; ^1^H NMR (400 MHz, CDCl_3_) *δ* 8.26 (2H, dd, *J* = 7.5, 2.0 Hz), 7.50 (3H, d, *J* = 7.1 Hz), 7.43–7.36 (4H, m), 7.31 (3H, dd, *J* = 14.5, 7.0 Hz), 7.22 (2H, d, *J* = 6.2 Hz), 6.87 (2H, d, *J* = 8.6 Hz), 6.77 (1H, dd, *J* = 6.7, 2.1 Hz), 6.40 (1H, s), 5.40 (1H, s), 4.62 (1H, d, *J* = 4.2 Hz), 4.59–4.42 (4H, m), 3.75 (3H, s), 1.04 (9H, s); HRESIMS (*m/z*): calcd. for C_37_H_37_N_2_O_8_, 637.2550; found 637.2561 [M + H]^+^.

Compound **6B**, colorless oil; yield 83%; ^1^H NMR (400 MHz, CDCl_3_) *δ* 8.21 (2H, d, *J* = 8.0 Hz), 7.53–7.49 (2H, m), 7.42–7.36 (4H, m), 7.34–7.26 (3H, m), 7.25–7.21 (2H, m), 6.90–6.85 (2H, m), 6.79–6.74 (1H, m), 6.39 (1H, s), 5.41 (1H, s), 4.62 (1H, d, *J* = 4.3 Hz), 4.60–4.40 (4H, m), 3.75 (3H, s), 1.36 (9H, s), 1.03 (6H, s); HRESIMS (*m/z*): calcd. for C_41_H_45_N_2_O_8_, 693.3176; found 693.3164 [M + H]^+^.

Compound **6C**, colorless oil; yield 76%; ^1^H NMR (400 MHz, CDCl_3_) *δ* 8.23 (2H, d, *J* = 8.3 Hz), 7.42–7.36 (4H, m), 7.32–7.29 (3H, m), 7.23–7.18 (2H, m), 7.00 (2H, dd, *J* = 9.3, 2.2 Hz), 6.87 (2H, dd, *J* = 9.1, 2.2 Hz), 6.79–6.72 (1H, m), 6.39 (1H, s), 5.41 (1H, s), 4.61 (1H, t, *J* = 4.2 Hz), 4.59–4.42 (4H, m), 3.88 (3H, s), 3.75 (3H, s), 1.04 (9H, s); HRESIMS (*m/z*): calcd. for C_38_H_39_N_2_O_9_, 667.2656; found 667.2584 [M + H]^+^.

Compound **6D**, colorless oil; yield 63%; ^1^H NMR (400 MHz, CDCl_3_) *δ* 8.28–8.22 (2H, m), 7.41–7.36 (4H, m), 7.35–7.28 (3H, m), 7.25–7.21 (2H, m), 7.20–7.15 (2H, m), 6.89–6.85 (2H, m), 6.77 (1H, dd, *J* = 7.5, 1.4 Hz), 6.40 (1H, s), 5.41 (1H, s), 4.62 (1H, d, *J* = 4.3 Hz), 4.59–4.41 (4H, m), 3.76 (3H, s), 1.04 (9H, s); HRESIMS (*m/z*): calcd. for C_37_H_36_FN_2_O_8_, 655.2456; found 655.2468 [M + H]^+^.

Compound **6E**, colorless oil; yield 71%; ^1^H NMR (400 MHz, CDCl_3_) *δ* 8.37 (1H, d, *J* = 8.1 Hz), 7.75 (1H, d, *J* = 8.3 Hz), 7.43–7.35 (2H, m), 7.35–7.27 (2H, m), 7.24 (1H, dd, *J* = 8.2, 1.1 Hz), 6.87 (1H, d, *J* = 8.7 Hz), 6.80 (1H, d, *J* = 7.8 Hz), 6.38 (1H, s), 5.41 (1H, s), 4.62 (1H, d, *J* = 4.3 Hz), 4.60–4.41 (4H, m), 3.76 (2H, s), 1.04 (9H, s); HRESIMS (*m/z*): calcd. for C_38_H_36_F_3_N_2_O_8_, 705.2424; found 705.2429 [M + H]^+^.

Compound **6F**, colorless oil; yield 87%; ^1^H NMR (400 MHz, CDCl_3_) *δ* 8.78 (1H, s), 8.33 (1H, dd, *J* = 8.6, 1.7 Hz), 8.00–7.87 (3H, m), 7.60–7.53 (2H, m), 7.43–7.36 (4H, m), 7.36–7.26 (5H, m), 6.87 (2H, d, *J* = 8.7 Hz), 6.79 (1H, t, *J* = 4.5 Hz), 6.39 (1H, s), 5.42 (1H, s), 4.63 (1H, d, *J* = 4.2 Hz), 4.60–4.44 (4H, m), 3.75 (3H, s), 1.57 (9H, s); HRESIMS (*m/z*): calcd. for C_41_H_39_N_2_O_8_, 687.2706; found 687.2719 [M + H]^+^.

Compound **6G**, colorless oil; yield 72%; ^1^H NMR (400 MHz, CDCl_3_) *δ* 9.51–9.44 (1H, m), 8.74 (1H, dd, *J* = 4.8, 1.7 Hz), 8.52 (1H, dt, *J* = 8.0, 1.9 Hz), 7.43 (1H, ddd, *J* = 8.0, 4.9, 0.7 Hz), 7.41–7.36 (4H, m), 7.34–7.27 (4H, m), 7.26–7.23 (1H, m), 6.89–6.84 (2H, m), 6.79 (1H, dd, *J* = 7.7, 1.1 Hz), 6.38 (1H, s), 5.41 (1H, s), 4.61 (1H, d, *J* = 4.2 Hz), 4.58–4.41 (4H, m), 3.76 (3H, s), 1.04 (9H, s); HRESIMS (*m/z*): calcd. for C_36_H_36_N_3_O_8_, 638.2502; found 638.2487 [M + H]^+^.

Compound **6H**, colorless oil; yield 78%; ^1^H NMR (400 MHz, CDCl_3_) *δ* 8.46 (1H, d, *J* = 2.9 Hz), 8.34 (1H d, *J* = 8.8 Hz), 7.41–7.36 (4H, m), 7.34–7.27 (6H, m), 6.87 (2H, d, *J* = 8.6 Hz), 6.78 (1H, dd, *J* = 7.2, 1.6 Hz), 6.39 (1H, s), 5.42 (1H, s), 4.62 (1H, d, *J* = 4.2 Hz), 4.59–4.40 (4H, m), 3.94 (3H, s), 3.75 (3H, s), 1.03 (9H, s); HRESIMS (*m/z*): calcd. for C_37_H_38_N_3_O_9_, 668.2608; found 668.2581 [M + H]^+^.

Compound **6I**, colorless oil; yield 67%; ^1^H NMR (400 MHz, CDCl_3_) *δ* 9.03 (1H, s), 8.52 (1H, d, *J* = 8.3 Hz), 8.08–8.05 (1H, m), 7.40–7.35 (5H, m), 7.34–7.29 (4H, m), 6.87 (2H, d, *J* = 8.7 Hz), 6.82 (1H, dd, *J* = 7.0, 1.8 Hz), 6.40 (1H, s), 5.42 (1H, s), 4.62 (1H, d, *J* = 4.3 Hz), 4.60–4.40 (4H, m), 3.76 (3H, s), 1.03 (9H, s); HRESIMS (*m/z*): calcd. for C_37_H_35_F_3_N_3_O_8_, 706.2376; found 706.2344 [M + H]^+^.

Compound **6J**, colorless oil; yield 80%; ^1^H NMR (400 MHz, CDCl_3_) *δ* 8.64 (1H, d, *J* = 5.0 Hz), 8.25 (1H, s), 7.41–7.36 (4H, m), 7.34–7.28 (5H, m), 7.24 (1H, d, *J* = 0.9 Hz), 6.86 (2H, d, *J* = 8.7 Hz), 6.78 (1H, dd, *J* = 6.4, 2.6 Hz), 6.39 (1H, s), 5.41 (1H, s), 4.61 (1H, d, *J* = 4.2 Hz), 4.57–4.44 (4H, m), 3.74 (3H, s), 2.44 (3H, s), 1.03 (9H, s); HRESIMS (*m/z*): calcd. for C_37_H_38_N_3_O_8_, 652.2659; found 652.2653 [M + H]^+^.

Compound **6K**, colorless oil; yield 77%; ^1^H NMR (400 MHz, CDCl_3_) *δ* 9.73 (1H, d, *J* = 2.1 Hz,), 9.01 (1H, d, *J* = 1.9 Hz), 8.18 (1H, d, *J* = 8.4 Hz), 7.95 (1H, d, *J* = 7.9 Hz), 7.82 (1H, m), 7.66–7.61 (1H, m), 7.42–7.36 (4H, m), 7.35–7.27 (5H, m), 6.91–6.85 (2H, m), 6.81 (1H, dd, *J* = 6.6, 2.3 Hz), 6.40 (1H, s), 5.42 (1H, s), 4.62 (1H, d, *J* = 4.2 Hz), 4.61–4.41 (4H, m), 3.76 (3H, s), 1.04 (9H, s); HRESIMS (*m/z*): calcd. for C_40_H_38_N_3_O_8_, 688.2659; found 688.2651 [M + H]^+^.

Compound **6L**, colorless oil; yield 72%; ^1^H NMR (400 MHz, CDCl_3_) *δ* 7.90 (1H, dd, *J* = 3.7, 1.2 Hz), 7.52 (1H, dd, *J* = 5.0, 1.2 Hz), 7.39–7.37 (4H, m), 7.33 (2H, t, J = 7.2 Hz), 7.30–7.27 (1H, m), 7.25–7.14 (3H, m), 6.90–6.85 (2H, m), 6.76 (1H, dd, *J* = 7.7, 1.1 Hz), 6.39 (1H, s), 5.40 (1H, s), 4.61 (1H, d, *J* = 4.2 Hz), 4.59–4.40 (4H, m), 3.76 (3H, s), 1.05 (9H, s); HRESIMS (*m/z*): calcd. for C_35_H_35_N_2_O_8_S, 643.2114; found 643.2084 [M + H]^+^.

Compound **6M**, colorless oil; yield 68%; ^1^H NMR (400 MHz, CDCl_3_) *δ* 7.64 (1H, d, *J* = 0.9 Hz), 7.38 (4H, d, *J* = 8.5 Hz), 7.32–7.31 (3H, m), 7.25–7.18 (3H, m), 6.87 (2H, d, *J* = 8.7 Hz), 6.77 (1H, d, *J* = 7.8 Hz), 6.59 (1H, dd, *J* = 3.5, 1.7 Hz), 5.40 (1H d, *J* = 2.4 Hz), 4.61 (1H, d, *J* = 4.2 Hz), 4.54–4.40 (4H, m), 3.76 (3H, s), 1.05 (9H, s); HRESIMS (*m/z*): calcd. for C_35_H_35_N_2_O_9_, 627.2343; found: 627.2318 [M + H]^+^.

### General procedure for the synthesis of compounds 7a–7**m** and 7A–7M

Ester compound **6** (0.10 mmol) was added to MeOH (2.0 mL) and treated with *p*-TsOH (0.20 mmol). The reaction mixture was stirred at room temperature for 3 h and then diluted with ethyl acetate. The organic layer was washed with a saturated aqueous solution of NaHCO_3_ and brine, dried with Na_2_SO_4_, filtered and concentrated. The crude mixture was purified by silica gel column chromatography (petroleum ether/EtOAc) to afford the desired hybrids **7a**–**7m** and **7A**–**7M** as white solid, yield 40–94%.

Compound **7a**, white solid; yield 52%; ^1^H NMR (400 MHz, CDCl_3_) *δ* 8.17 (2H, d, *J* = 8.5 Hz), 7.75 (2H, d, *J* = 7.3 Hz), 7.57–7.27 (10H, m), 7.23 (3H, t, *J* = 4.8 Hz), 6.83–6.81 (1H, m), 5.74–5.71 (1H, m), 4.77–4.75 (1H, m), 4.73–4.54 (4H, m), 3.86 (1H, d, *J* = 5.4 Hz); ^13^C NMR (100 MHz, CDCl_3_) *δ* 173.0, 167.5, 162.1, 152.4, 150.0, 138.0, 134.2, 131.9, 131.6, 131.5, 128.9, 128.7, 128.5, 128.0, 127.7, 127.3, 127.2, 127.0, 125.9, 108.4, 104.3, 73.5, 66.8, 64.4, 55.8; HRESIMS (*m/z*): calcd. for C_31_H_27_N_2_O_6_, 523.1869; found 523.1891 [M + H]^+^.

Compound **7b**, white solid; yield 73%; ^1^H NMR (400 MHz, CDCl_3_) *δ* 8.03 (2H, d, *J* = 8.5 Hz), 7.84 (2H, d, *J* = 7.3 Hz), 7.62 (1H, d, *J* = 8.8 Hz), 7.49 (2H, d, *J* = 7.2 Hz), 7.43 (2H, d, *J* = 8.5 Hz), 7.38 (1H, t, *J* = 7.4 Hz), 7.34–7.27 (2H, m), 7.25–7.19 (4H, m), 6.81–6.80 (1H, m), 5.73–5.70 (1H, m), 4.77 (1H, d, *J* = 2.2 Hz), 4.69–4.60 (4H, m), 4.03 (1H, s), 1.34 (9H, s); ^13^C NMR (100 MHz, CDCl_3_) *δ* 173.0, 167.5, 162.3, 155.1, 152.3, 149.9, 138.0, 134.1, 132.0, 131.6, 128.7, 128.5, 128.0, 127.5, 127.3, 127.2, 125.9, 125.6, 124.1, 108.3, 104.2, 73.5, 66.7, 64.3, 56.0, 35.1, 31.2; HRESIMS (*m/z*): calcd. for C_35_H_35_N_2_O_6_, 579.2495; found 579.2470 [M + H]^+^.

Compound **7c**, white solid; yield 52%; ^1^H NMR (400 MHz, CD_3_OD) *δ* 8.08 (2H, d, *J* = 8.4 Hz), 7.73 (2H, d, *J* = 7.0 Hz), 7.49–7.41 (3H, m), 7.39–7.24 (7H, m), 7.05 (2H, d, *J* = 8.3 Hz), 6.85 (1H, d, *J* = 7.0 Hz), 5.62 (1H, s), 4.72–4.44 (5H, m), 3.87 (3H, s); ^13^C NMR (100 MHz, CD_3_OD) *δ* 173.6, 169.9, 164.1, 151.3, 151.3, 139.9, 135.4, 132.7, 132.6, 130.3, 130.3, 129.4, 129.4, 128.7, 128.4, 128.3, 126.7, 120.4, 115.5, 109.3, 104.7, 74.9, 68.1, 64.9, 57.8, 56.0; HRESIMS (*m/z*): calcd. for C_32_H_29_N_2_O_7_, 553.1975; found 553.1960 [M + H]^+^.

Compound **7d**, white solid; yield 51%; ^1^H NMR (400 MHz, CDCl_3_) *δ* 8.11 (2H, d, *J* = 8.5 Hz), 7.73 (2H, d, *J* = 7.6 Hz), 7.48 (3H, t, *J* = 5.8 Hz), 7.38 (1H, t, *J* = 7.4 Hz), 7.34–7.27 (3H, m), 7.24–7.18 (3H, m), 7.11 (2H, t, *J* = 8.6 Hz), 6.82 (1H, d, *J* = 7.8 Hz), 5.73–5.72 (1H, m), 4.75 (1H, s), 4.72–4.50 (4H, m), 3.80 (1H, d, *J* = 4.4 Hz); ^13^C NMR (100 MHz, CDCl_3_) *δ* 173.0, 167.4, 166.1, 162.4 (d, *J* = 235.7 Hz), 161.2, 152.4, 150.0, 138.0, 134.2, 131.9, 131.7, 129.9 (d, *J* = 8.9 Hz), 128.8, 128.5, 128.1, 127.3, 127.2, 125.9, 123.3, 116.3, 116.1, 108.5, 104.2, 73.4, 66.8, 64.5, 55.8; HRESIMS (*m/z*): calcd. for C_31_H_26_FN_2_O_6_, 541.1775; found 541.1791 [M + H]^+^.

Compound **7e**, white solid; yield 45%; ^1^H NMR (400 MHz, CDCl_3_) *δ* 8.22 (2H, d, *J* = 8.1 Hz), 7.76–7.65 (4H, m), 7.49 (2H, d, *J* = 7.0 Hz), 7.43–7.35 (2H, m), 7.30 (3H, dt, *J* = 5.2, 4.1 Hz), 7.26–7.20 (3H, m), 6.85 (1H, dd, *J* = 8.0, 0.7 Hz), 5.75 (1H, dd, *J* = 9.0, 2.6 Hz), 4.74 (1H, d, *J* = 3.2 Hz), 4.73–4.53 (4H, m), 3.72 (1H, d, *J* = 4.5 Hz); ^13^C NMR (100 MHz, CDCl_3_) *δ* 173.0, 167.3, 160.5, 152.5, 150.4, 138.0, 134.2, 133.0 (d, *J* = 32.8 Hz), 131.8, 131.7, 130.2, 130.0, 128.8, 128.5, 128.1, 127.9, 127.3, 127.1, 126.6, 125.9 (q, *J* = 3.6 Hz), 125.2, 122.5, 108.6, 104.3, 73.4, 66.9, 64.5, 55.7; HRESIMS (*m/z*): calcd. for C_32_H_26_F_3_N_2_O_6_, 591.1743; found 591.1744 [M + H]^+^.

Compound **7f**, white solid; yield 52%; ^1^H NMR (400 MHz, CDCl_3_) *δ* 8.65 (1H, s), 8.17 (1H, d, *J* = 8.3 Hz), 7.91–7.84 (3H, m), 7.75 (2H, d, *J* = 7.6 Hz), 7.54–7.51 (5H, m), 7.40–7.28 (5H, m), 7.24–7.19 (2H, m), 6.84 (1H, dd, *J* = 6.5, 1.9 Hz), 5.75 (1H, d, *J* = 6.9 Hz), 4.78 (1H, s), 4.74–4.58 (4H, m), 3.80 (1H, s); ^13^C NMR (100 MHz, CDCl_3_) *δ* 173.1, 167.5, 162.3, 152.6, 150.1, 138.0, 134.8, 134.2, 133.0, 132.1, 131.7, 129.1, 128.8, 128.8, 128.5, 128.2, 128.1, 128.0, 127.9, 127.3, 127.2, 127.0, 125.9, 124.3, 124.1, 108.5, 104.3, 73.5, 66.9, 64.5, 55.9; HRESIMS (*m/z*): calcd. for C_35_H_29_N_2_O_6_, 573.2026; found 573.2045 [M + H]^+^.

Compound **7g**, white solid; yield 48%; ^1^H NMR (400 MHz, CDCl_3_) *δ* 9.32 (1H, d, *J* = 1.6 Hz), 8.67 (1H, dd, *J* = 4.8, 1.5 Hz), 8.37–8.31 (1H, m), 7.71 (2H, d, *J* = 7.3 Hz), 7.48 (2H, d, *J* = 7.2 Hz), 7.44–7.39 (1H, m), 7.38–7.33 (2H, m), 7.32–7.27 (3H, m), 7.25–7.20 (3H, m), 6.84 (1H, d, *J* = 7.9 Hz), 5.75 (1H, dd, *J* = 9.0, 2.5 Hz), 4.74 (1H, s), 4.70–4.53 (4H, m), 4.04 (1H, d, *J* = 3.4 Hz); ^13^C NMR (100 MHz, CDCl_3_) *δ* 172.9, 167.3, 159.6, 152.4, 151.9, 150.2, 148.6, 138.1, 134.9, 134.1, 131.6, 131.6, 128.7, 128.5, 128.0, 127.2, 127.1, 126.5, 123.8, 123.4, 108.6, 104.3, 73.4, 66.9, 64.4, 55.6; HRESIMS (*m/z*): calcd. for C_30_H_26_N_3_O_6_, 524.1822; found 524.1838 [M + H]^+^.

Compound **7h**, white solid; yield 50%; ^1^H NMR (600 MHz, CDCl_3_) *δ* 8.38 (1H, d, *J* = 2.8 Hz), 8.09 (1H, d, *J* = 8.7 Hz), 7.74–7.72 (2H, m), 7.56 (1H, d, *J* = 8.9 Hz), 7.51 (2H, d, *J* = 7.4 Hz), 7.36 (1H, t, *J* = 7.4 Hz,), 7.32–7.28 (2H, m), 7.27 (1H, d, *J* = 2.4 Hz), 7.25–7.18 (4H, m), 6.85–6.81 (1H, m), 5.74 (1H, dd,* J* = 8.9, 2.7 Hz), 4.78 (1H, s), 4.69–4.55 (4H, m), 4.01 (1H, d, *J* = 4.5 Hz), 3.91 (3H, s); ^13^C NMR (100 MHz, CDCl_3_) *δ* 173.0, 167.4, 160.6, 157.3, 152.6, 150.3, 138.4, 138.3, 138.2, 134.2, 131.9, 131.6, 128.7, 128.4, 128.0, 127.3, 127.2, 126.2, 124.7, 120.7, 108.8, 104.8, 73.5, 67.1, 64.4, 55.9, 55.8; HRESIMS (*m/z*): calcd. for C_31_H_28_N_3_O_7_, 554.1927; found 554.1928 [M + H]^+^.

Compound **7i**, white solid; yield 40%; ^1^H NMR (400 MHz, CDCl_3_) *δ* 8.97 (1H, s), 8.28 (1H, d, *J* = 8.3 Hz), 7.99 (1H, dd, *J* = 8.3, 1.9 Hz), 7.72–7.67 (2H, m), 7.50 (2H, d, *J* = 7.2 Hz), 7.38–7.27 (6H, m), 7.21 (2H, t, *J* = 7.8 Hz), 6.88 (1H, dd, *J* = 7.7, 0.8 Hz), 5.76 (1H, dd, *J* = 9.1, 2.6 Hz), 4.79–4.75 (1H, m), 4.71–4.55 (4H, m), 3.73 (1H, d, *J* = 4.9 Hz); ^13^C NMR (150 MHz, CDCl_3_) *δ* 173.0, 167.3, 159.1, 152.8, 150.8, 148.8, 147.1 (q, *J* = 3.9 Hz), 138.1, 134.5 (q, *J* = 3.3 Hz), 134.1, 131.7, 128.7, 128.5, 128.1, 127.9 (d, *J* = 33.5 Hz), 127.7, 127.3, 127.1, 123.2, 123.1 (d, *J* = 272.8 Hz), 108.7, 104.9, 73.4, 67.0, 64.4, 55.6; HRESIMS (*m/z*): calcd. for C_31_H_25_F_3_N_3_O_6_, 592.1695; found 592.1684 [M + H]^+^.

Compound **7j**, white solid; yield 52%; ^1^H NMR (600 MHz, CDCl_3_) *δ* 8.57 (1H, d, *J* = 5.0 Hz), 8.00 (1H, s), 7.73–7.71 (2H, m), 7.53–7.51 (3H, m), 7.37–7.33 (1H, m), 7.30–7.29 (4H, m), 7.22–7.18 (3H, m), 6.84 (1H, dd, *J* = 6.3, 2.6 Hz), 5.73 (1H, dd, *J* = 9.0, 2.7 Hz), 4.78 (1H, dd, *J* = 5.2, 2.8 Hz), 4.71–4.57 (4H, m), 3.98 (1H, d, *J* = 5.3 Hz), 2.33 (3H, s); ^13^C NMR (150 MHz, CDCl_3_) *δ* 173.0, 167.4, 160.7, 152.7, 150.5, 149.9, 148.7, 145.6, 138.1, 134.2, 131.8, 131.6, 128.7, 128.4, 128.0, 127.4, 127.1, 126.7, 126.6, 124.5, 108.6, 104.9, 73.4, 67.0, 64.4, 55.8, 21.1; HRESIMS (*m/z*): calcd. for C_31_H_28_N_3_O_6_, 538.1978; found 538.1969 [M + H]^+^.

Compound **7k**, white solid; yield 42%; ^1^H NMR (400 MHz, CDCl_3_) *δ* 9.60 (1H, d, *J* = 2.1 Hz), 8.86 (1H, d, *J* = 1.8 Hz), 8.14 (1H, d, *J* = 8.5 Hz), 7.84 (1H, d, *J* = 8.1 Hz), 7.82–7.77 (1H, m), 7.74–7.70 (2H, m), 7.63–7.58 (1H, m), 7.51 (2H, d, *J* = 7.3 Hz), 7.41–7.35 (2H, m), 7.32 (2H, dd, *J* = 7.6, 2.4 Hz), 7.30–7.27 (2H, m), 7.26–7.21 (2H, m), 6.86 (1H, dd, *J* = 7.5, 1.4 Hz), 5.77 (1H, dd, *J* = 9.0, 2.5 Hz), 4.77 (1H, d, *J* = 2.2 Hz), 4.74–4.56 (4H, m), 3.82 (1H, s); ^13^C NMR (100 MHz, CDCl_3_) *δ* 173.0, 167.3, 160.0, 152.5, 150.3, 149.1, 148.5, 138.1, 135.5, 134.2, 131.8, 131.7, 131.4, 129.6, 128.8, 128.8, 128.5, 128.1, 127.8,127.3, 127.2, 126.5, 120.3, 108.7, 104.3, 73.5, 67.0, 64.5, 55.6; HRESIMS (*m/z*): calcd. for C_34_H_28_N_3_O_6_, 574.1978; found 574.1979 [M + H]^+^.

Compound **7l**, white solid; yield 64%; ^1^H NMR (400 MHz, CDCl_3_) *δ* 7.79 (1H, dd, *J* = 3.7, 1.1 Hz), 7.77–7.72 (2H, m), 7.52–7.45 (4H, m), 7.38 (1H, t, *J* = 7.4 Hz), 7.34–7.29 (2H, m), 7.25 (3H, t, *J* = 10.3 Hz), 7.20–7.17 (1H, m), 7.12 (1H, dd, *J* = 5.0, 3.8 Hz), 6.81 (1H, d, *J* = 7.9 Hz), 5.72 (1H, dd, *J* = 8.9, 2.7 Hz), 4.75 (1H, d, *J* = 2.8 Hz), 4.70–4.53 (4H, m), 3.84 (1H, s); ^13^C NMR (100 MHz, CDCl_3_) *δ* 172.9, 167.4, 158.1, 152.0, 149.9, 138.1, 134.1, 131.8, 131.6, 130.3, 130.1, 129.3, 128.7, 128.5, 128.3, 128.0, 127.3, 127.2, 125.8, 108.5, 104.1, 73.6, 66.8, 64.3, 55.8; HRESIMS (*m/z*): calcd. for C_29_H_25_N_2_O_6_S, 529.1433; found 529.1421 [M + H]^+^.

Compound **7m**, white solid; yield 42%; ^1^H NMR (600 MHz, CDCl_3_) *δ* 7.78 (2H, d, *J* = 7.3 Hz), 7.58 (1H, d, *J* = 0.9 Hz), 7.54 (1H, d, *J* = 8.9 Hz), 7.47 (2H, d, *J* = 7.5 Hz), 7.40 (1H, t, *J* = 7.4 Hz), 7.31–7.26 (4H, m), 7.26–7.22 (2H, dd, *J* = 7.7, 5.3 Hz), 7.20 (1H, d, *J* = 7.8 Hz), 7.17 (1H, d, *J* = 3.3 Hz), 6.82 (1H, d, *J* = 8.0 Hz), 6.56 (1H, dd, *J* = 3.4, 1.7 Hz), 5.71 (1H, dd, *J* = 8.9, 2.4 Hz), 4.77 (1H, d, *J* = 1.8 Hz), 4.68–4.62 (2H, m), 4.61–4.56 (1H, m), 4.55–4.49 (1H, m), 4.05 (1H, s); ^13^C NMR (150 MHz, CDCl_3_) *δ* 173.0, 167.5, 154.4, 151.8, 150.2, 145.8, 142.4, 138.2, 134.2, 131.7, 131.5, 128.7, 128.5, 127.9, 127.3, 127.2, 126.1, 114.5, 112.4, 108.9, 104.2, 73.5, 67.0, 64.3, 55.8; HRESIMS (*m/z*): calcd. for C_29_H_25_N_2_O_7_, 513.1662; found 513.1678 [M + H]^+^.

Compound **7A**, white solid; yield 72%; ^1^H NMR (400 MHz, CDCl_3_) *δ* 8.24–8.20 (2H, m), 7.49 (3H, q, *J* = 6.4 Hz), 7.40 (2H, d, *J* = 7.4 Hz), 7.34–7.27 (3H, m), 7.26–7.22 (2H, m), 6.89 (1H, d, *J* = 7.6 Hz), 5.75 (1H, d, *J* = 8.8 Hz), 5.22 (1H, d, *J* = 8.6 Hz), 4.68–4.50 (5H, m), 3.47 (1H, s), 1.35 (9H, s); ^13^C NMR (100 MHz, CDCl_3_) *δ* 173.0, 162.0, 155.5, 152.5, 150.2, 138.7, 132.0, 131.5, 128.9, 128.6, 127.8, 127.7, 127.1, 127.0, 125.8, 108.8, 104.2, 79.8, 73.6, 67.0, 64.4, 56.6, 28.3; HRESIMS (*m/z*): calcd. for C_29_H_31_N_2_O_7_, 519.2131; found 519.2121 [M + H]^+^.

Compound **7B**, white solid; yield 70%; ^1^H NMR (400 MHz, CDCl_3_) *δ* 8.14 (2H, d, *J* = 8.4 Hz), 7.50 (2H, d, *J* = 8.5 Hz), 7.40 (2H, d, *J* = 7.3 Hz), 7.38–7.34 (1H, m), 7.31 (2H, t, *J* = 7.3 Hz), 7.29–7.26 (1H, m), 7.26–7.21 (2H, m), 6.89 (1H, d, *J* = 7.2 Hz), 5.74 (1H, d, *J* = 8.9 Hz), 5.22 (1H, d, *J* = 8.2 Hz), 4.71–4.57 (5H, m), 3.42 (1H, s), 1.37 (9H, s); ^13^C NMR (100 MHz, CDCl_3_) *δ* 173.1, 162.3, 155.5, 155.1, 152.5, 150.1, 132.2, 128.7, 127.8, 127.6, 127.1, 126.9, 125.9, 125.6, 124.3, 108.8, 104.2, 79.9, 73.7, 67.0, 64.4, 56.6, 35.2, 31.3, 28.3; HRESIMS (*m/z*): calcd. for C_33_H_39_N_2_O_7_, 575.2757; found 575.2738 [M + H]^+^.

Compound **7C**, white solid; yield 68%; ^1^H NMR (400 MHz, CDCl_3_) *δ* 8.15 (2H, d, *J* = 8.7 Hz), 7.39 (2H, d, *J* = 7.3 Hz), 7.31 (2H, t, *J* = 7.4 Hz), 7.26 (1H, d, *J* = 7.1 Hz), 7.24–7.19 (2H, m), 6.97 (2H, d, *J* = 8.8 Hz), 6.87 (1H, d, *J* = 7.2 Hz), 5.76 (1H, d, *J* = 8.9 Hz), 5.22 (1H, d, *J* = 8.5 Hz), 4.68–4.58 (5H, m), 3.87 (3H, s), 3.49 (1H, s), 1.34 (9H, s); ^13^C NMR (100 MHz, CDCl_3_) *δ* 173.0, 162.3, 162.2, 155.5, 152.4, 149.9, 138.7, 132.1, 129.5, 128.6, 127.8, 127.1, 125.3, 119.6, 114.4, 108.7, 104.1, 79.8, 73.6, 66.9, 64.4, 56.6, 55.6, 28.3; HRESIMS (*m/z*): calcd. for C_30_H_33_N_2_O_8_, 549.2237; found 549.2248 [M + H]^+^.

Compound **7D**, white solid; yield 93%; ^1^H NMR (400 MHz, CDCl_3_) *δ* 8.20 (2H, dd, *J* = 8.6, 5.4 Hz), 7.39 (2H, d, *J* = 7.4 Hz), 7.30 (3H, m), 7.26–7.20 (2H, m), 7.15 (2H, t, *J* = 8.6 Hz), 6.89 (1H, d, *J* = 7.8 Hz), 5.73 (1H, d, *J* = 8.8 Hz), 5.22 (1H, d, *J* = 8.7 Hz), 4.70–4.60 (5H, m), 3.45 (1H, d, *J* = 4.2 Hz), 1.34 (9H, s); ^13^C NMR (100 MHz, CDCl_3_) *δ* 173.0, 166.1, 162.4 (d, *J* = 243.6 Hz), 161.2, 155.5, 152.5, 150.2, 138.7, 132.0, 130.0 (d, *J* = 8.9 Hz), 128.6, 127.8, 127.1, 125.9, 123.4, 123.4, 116.3, 116.1, 108.8, 104.1, 79.8, 73.6, 67.0, 64.4, 56.6, 28.3; HRESIMS (*m/z*): calcd. for C_29_H_30_FN_2_O_7_, 537.2037; found 537.2015 [M + H]^+^.

Compound **7E**, white solid; yield 57%; ^1^H NMR (400 MHz, CDCl_3_) *δ* 8.33 (2H, d, *J* = 8.1 Hz), 7.73 (2H, d, *J* = 8.3 Hz), 7.40 (2H, d, *J* = 7.3 Hz), 7.32–7.27 (3H, m), 7.29–7.23 (2H, m), 6.92 (1H, d, *J* = 8.0 Hz), 5.67 (1H, d, *J* = 8.7 Hz), 5.22 (1H, d, *J* = 8.4 Hz), 4.70–4.58 (5H, m), 3.34 (1H, s), 1.34 (9H, s); ^13^C NMR (100 MHz, CDCl_3_) *δ* 173.0, 160.5, 155.4, 152.6, 150.5, 138.7, 133.0 (d, *J* = 32.7 Hz), 131.9, 130.4, 128.7, 128.0, 127.9, 127.1, 126.6, 125.9 (q, *J* = 3.8 Hz), 125.2, 122.5, 108.9, 104.3, 79.9, 73.6, 67.0, 64.4, 56.6, 28.3; HRESIMS (*m/z*): calcd. for C_30_H_30_F_3_N_2_O_7_, 587.2005; found 587.2017 [M + H]^+^.

Compound **7F**, white solid; yield 57%; ^1^H NMR (600 MHz, CDCl_3_) *δ* 8.74 (1H, s), 8.28 (1H, d, *J* = 8.5 Hz), 7.92 (2H, d, *J* = 8.3 Hz), 7.88 (1H, d, *J* = 7.7 Hz), 7.58–7.53 (2H, m), 7.42 (2H, d, *J* = 7.2 Hz), 7.32–7.30 (3H, m), 7.29–7.27 (2H, m), 6.92 (1H, d, *J* = 7.4 Hz), 5.76 (1H, d, *J* = 8.9 Hz), 5.24 (1H, d, *J* = 8.9 Hz), 4.73–4.60 (5H, m), 3.40 (1H, d, *J* = 3.7 Hz), 1.35 (9H, s); ^13^C NMR (150 MHz, CDCl_3_) *δ* 173.1, 162.2, 155.5, 152.6, 150.3, 138.7, 134.8, 133.1, 132.2, 129.1, 128.8, 128.7, 128.2, 128.0, 127.9, 127.1, 127.0, 125.9, 124.4, 124.2, 108.7, 104.2, 79.9, 73.6, 67.0, 64.4, 56.6, 28.3; HRESIMS (*m/z*): calcd. for C_33_H_33_N_2_O_7_, 569.2288; found 569.2262 [M + H]^+^.

Compound **7G**, white solid; yield 51%; ^1^H NMR (400 MHz, CDCl_3_) *δ* 9.40 (1H, d, *J* = 1.9 Hz), 8.70 (1H, dd, *J* = 4.8, 1.6 Hz), 8.45 (1H, d, *J* = 8.0 Hz), 7.42–7.36 (3H, m), 7.33–7.28 (3H, m), 7.26–7.22 (2H, m), 6.91 (1H, d, *J* = 7.9 Hz), 5.71 (1H, d, *J* = 8.9 Hz), 5.22 (1H, d, *J* = 8.8 Hz), 4.69–4.56 (5H, m), 3.65 (1H, s), 1.34 (9H, s); ^13^C NMR (100 MHz, CDCl_3_) *δ* 173.0, 159.6, 155.4, 152.5, 152.0, 150.4, 148.7, 138.8, 134.9, 131.7, 128.6, 127.8, 127.0, 126.5, 123.7, 123.5, 109.0, 104.2, 79.9, 73.6, 67.0, 64.3, 56.6, 28.3; HRESIMS (*m/z*): calcd. for C_28_H_30_N_3_O_7_, 520.2084; found 520.2097 [M + H]^+^.

Compound **7H**, white solid; yield 52%; ^1^H NMR (400 MHz, CDCl_3_) *δ* 8.43 (1H, d, *J* = 2.8 Hz), 8.25 (1H, d, *J* = 8.7 Hz), 7.40 (2H, d, *J* = 7.3 Hz), 7.39–7.26 (5H, m), 7.24 (1H, t, *J* = 4.8 Hz), 6.92–6.87 (1H, m), 5.73 (1H, d, *J* = 9.0 Hz), 5.22 (1H, d, *J* = 8.5 Hz), 4.68–4.57 (5H, m), 3.93 (3H, s), 3.58 (3H, s), 1.33 (9H, s); ^13^C NMR (100 MHz, CDCl_3_) *δ* 173.0, 160.6, 157.4, 155.5, 152.7, 150.4, 138.4, 131.9, 128.6, 127.8, 127.1, 126.2, 124.8, 120.7, 109.0, 104.7, 79.9, 73.7, 67.2, 64.3, 56.6, 56.0, 28.3; HRESIMS (*m/z*): calcd. for C_29_H_32_N_3_O_8_, 550.2189; found 550.2189 [M + H]^+^.

Compound **7I**, white solid; yield 90%; ^1^H NMR (400 MHz, CDCl_3_) *δ* 9.06–8.97 (1H, m), 8.42 (1H, d, *J* = 8.1 Hz), 8.04 (1H, d, *J* = 8.1 Hz), 7.40–7.38 (3H, m), 7.35–7.28 (3H, m), 7.27–7.22 (1H, m), 6.94 (1H, d, *J* = 7.8 Hz), 5.66 (1H, d, *J* = 9.0 Hz), 5.22 (1H, d, *J* = 8.8 Hz), 4.72–4.56 (5H, m), 3.43 (1H, d, *J* = 5.1 Hz), 1.32 (9H, s); ^13^C NMR (100 MHz, CDCl_3_) *δ* 173.0, 159.1, 155.4, 152.9, 150.9, 149.0, 147.1 (dd, *J* = 7.9, 3.9 Hz), 138.7, 134.5 (q, *J* = 3.3 Hz), 131.8, 128.7, 128.0 (d, *J* = 33.5 Hz), 127.9, 127.6, 127.1, 123.3, 123.2 (d, *J* = 272.8 Hz), 109.1, 104.8, 79.9, 73.6, 67.1, 64.3, 56.6, 28.3; HRESIMS (*m/z*): calcd. for C_29_H_29_F_3_N_3_O_7_, 588.1958; found 588.1942 [M + H]^+^.

Compound **7J**, white solid; yield 70%; ^1^H NMR (400 MHz, CDCl_3_) *δ* 8.58 (1H, d, *J* = 5.0 Hz), 8.12 (1H, s), 7.40 (2H, d, *J* = 7.2 Hz), 7.33–7.27 (4H, m), 7.22 (2H, dd, *J* = 12.6, 5.7 Hz), 6.89 (1H, d, *J* = 6.3 Hz), 5.78 (1H, d, *J* = 8.9 Hz), 5.21 (1H, d, *J* = 8.3 Hz), 4.69–4.55 (5H, m), 3.66 (1H, d, *J* = 3.9 Hz), 2.36 (3H, s), 1.30 (9H, s); ^13^C NMR (100 MHz, CDCl_3_) *δ* 173.0, 160.7, 155.5, 152.8, 150.6, 150.0, 148.6, 145.8, 138.7, 131.9, 128.6, 127.8, 127.1, 126.7, 126.6, 124.5, 108.9, 104.8, 79.8, 73.6, 67.2, 64.3, 56.7, 28.3, 21.1; HRESIMS (*m/z*): calcd. for C_29_H_32_N_3_O_7_, 534.2240; found 534.2238 [M + H]^+^.

Compound **7K**, white solid; yield 94%; ^1^H NMR (400 MHz, CDCl_3_) *δ* 9.69 (1H, d, *J* = 2.1 Hz), 8.95 (1H, s), 8.16 (1H, d, *J* = 8.5 Hz), 7.90 (1H, d, *J* = 8.1 Hz), 7.82–7.77 (1H, m), 7.65–7.59 (1H, m), 7.41 (2H, d, *J* = 7.4 Hz), 7.36–7.29 (4H, m), 7.28–7.23 (1H, m), 6.93 (1H, d, *J* = 7.6 Hz), 5.67 (1H, d, *J* = 9.0 Hz), 5.24 (1H, d, *J* = 9.0 Hz), 4.73–4.64 (4H, m), 4.59 (1H, s), 3.46 (1H, d, *J* = 5.2 Hz), 1.35 (9H, s); ^13^C NMR (100 MHz, CDCl_3_) *δ* 173.1, 160.0, 155.4, 152.6, 150.5, 149.1, 148.6, 138.8, 135.5 131.9, 131.4, 129.7, 128.8, 128.7, 127.9, 127.8, 127.3, 127.1, 126.5, 120.4, 109.0, 104.3, 79.9, 73.6, 67.0, 64.4, 56.6, 28.3; HRESIMS (*m/z*): calcd. for C_32_H_32_N_3_O_7_, 570.2240; found 570.2219 [M + H]^+^.

Compound **7L**, white solid; yield 44%; ^1^H NMR (400 MHz, CDCl_3_) *δ* 7.63 (1H, d, *J* = 1.0 Hz), 7.38 (2H, d, *J* = 7.4 Hz), 7.34–7.27 (3H, m), 7.25 (2H, d, *J* = 3.2 Hz), 7.21 (1H, dd, *J* = 8.1, 0.7 Hz), 6.89 (1H, d, *J* = 7.9 Hz), 6.59 (1H, dd, *J* = 3.5, 1.7 Hz), 5.64 (1H, d, *J* = 9.1 Hz), 5.22 (1H, d, *J* = 8.5 Hz), 4.71–4.55 (5H, m), 3.45 (1H, d, *J* = 4.8 Hz), 1.37 (9H, s); ^13^C NMR (100 MHz, CDCl_3_) *δ* 173.0, 155.4, 154.3, 151.9, 150.3, 145.7, 142.6, 138.9, 131.6, 128.6, 127.8, 127.0, 126.0, 114.4, 112.4, 109.2, 104.2, 79.9, 73.7, 67.2, 64.4, 56.5, 28.4; HRESIMS (*m/z*): calcd. for C_27_H_29_N_2_O_8_, 509.1924; found 509.1934 [M + H]^+^.

Compound **7M**, white solid; yield 64%; ^1^H NMR (400 MHz, CDCl_3_) *δ* 7.88 (1H, dd, *J* = 3.6, 0.9 Hz), 7.52 (1H, dd, *J* = 5.0, 1.1 Hz), 7.39 (2H, d, *J* = 7.4 Hz), 7.31 (2H, t, *J* = 7.4 Hz), 7.28–7.22 (2H, m), 7.19 (1H, dd, *J* = 8.1, 0.8 Hz), 7.17–7.13 (1H, m), 6.88 (1H, d, *J* = 7.9 Hz), 5.69 (1H, d, *J* = 8.9 Hz), 5.22 (1H, d, *J* = 8.7 Hz), 4.69–4.53 (5H, m), 3.46 (1H, d, *J* = 4.7 Hz), 1.35 (9H, s); ^13^C NMR (100 MHz, CDCl_3_) *δ* 173.0, 158.1, 155.5, 152.1, 150.0, 138.8, 131.9, 130.2, 130.1, 129.5, 128.6, 128.3, 127.8, 127.0, 125.8, 109.0, 104.0, 79.9, 73.7, 67.0, 64.4, 56.6, 28.3; HRESIMS (*m/z*): calcd. for C_27_H_29_N_2_O_7_S, 525.1695; found 525.1707 [M + H]^+^.

### In vitro antiproliferative assay

The twenty-six newly synthesized hybrids were evaluated in vitro for their antiproliferative activity against human tumor cell lines (MDA-MB-231 and HepG-2) by MTT assay. MTT (M2128) was purchased from Sigma-Aldrich (St. Louis, Mo, USA). The MDA-MB-231 and HepG-2 cells were purchased from American Type Culture Collection (ATCC, Manassas, VA, USA). Cells were cultured in DMEM with 10% fetal bovine serum and incubated with 5% CO_2_. Tested cell lines were seeded at a density of 5 × 10^3^/well in a 96-well plate for 24 h, and then treated with different concentrations of compounds dissolved in 100% DMSO for 24 h, with the final DMSO concentrations lower than 0.1%. Control cells were treated with paclitaxel containing 0.1% DMSO. DMSO served as a negative control. Then, 10 μL MTT (5 mg/mL) were added into each well and incubated for another 4 h. The purple formazan crystals were solved in 100 μL DMSO and the absorbance was detected at 570 nm by a microplate reader (Thermo MK3, USA). The IC_50_ values were calculated according to the dose-dependent curves. All the tests were repeated in at least three independent experiments. Inhibition rate = (OD _control_ − OD _treated_) / (OD _control_ − OD _vacuity_) × 100%.

## Supplementary Information

Supplementary Information.

## Data Availability

All data generated or analyzed during this study are included in this published article and its supplementary information files.
