# Supplementary material for: Molecular-docking-guided design, palladium-catalyzed synthesis and anticancer activity of paclitaxel-benzoxazoles hybrids
Source: Sci Rep. 2022 Jun 15;12:10021. doi: 10.1038/s41598-022-14172-3 (PMC9200075; doi:10.1038/s41598-022-14172-3)
Supplement: Supplementary file 1 — Supplementary Information. [file 41598_2022_14172_MOESM1_ESM.pdf]

SUPPLEMENTARY INFORMATION

for

Molecular-docking-guided design, palladium-catalyzed synthesis and anticancer activity of paclitaxel-benzoxazoles hybrids

Ting Jiang[1], Ya-Nan Cao[2], Jin-Bu Xu[2], Feng Gao[2], Ling-Li Zheng[1,*]

[1] Department of Pharmacy, The First Afflicted Hospital of Chengdu Medical College, No. 278, Baoguang Rd, Xindu Region, Chengdu 610500, PR China

[2] School of Life Science and Engineering, Southwest Jiaotong University, No. 111, Erhuan Rd, Chengdu, 610031, People's Republic of China

* Corresponding author

Email: zhenglingli@cmc.edu.cn

Table of Content

Figure S1 ^{1}H NMR (400 MHz) spectrum of compound **3a** in $CDCl_3$ 5

Figure S2 ^{1}H NMR (400 MHz) spectrum of compound **3b** in $CDCl_3$ 5

Figure S3 ^{1}H NMR (400 MHz) spectrum of compound **3c** in $CDCl_3$......................6

Figure S4 ^{1}H NMR (400 MHz) spectrum of compound **3d** in $CDCl_3$ 6

Figure S5 ^{1}H NMR (400 MHz) spectrum of compound **3e** in $CDCl_3$......................7

Figure S6 ^{1}H NMR (400 MHz) spectrum of compound **3f** in $CDCl_3$ 7

Figure S7 ^{1}H NMR (400 MHz) spectrum of compound **3g** in $CDCl_3$ 8

Figure S8 ^{1}H NMR (400 MHz) spectrum of compound **3h** in $CDCl_3$ 8

Figure S9 ^{1}H NMR (400 MHz) spectrum of compound **3i** in $CDCl_3$ 9

F**Figure S10** ^{1}H NMR (400 MHz) spectrum of compound **3j** in $CDCl_3$..................9

Figure S11 ^{1}H NMR (400 MHz) spectrum of compound **3k** in $CDCl_3$ 10

Figure S12 ^{1}H NMR (400 MHz) spectrum of compound **3l** in $CDCl_3$ 10

Figure S13 ^{1}H NMR (400 MHz) spectrum of compound **3m** in $CDCl_3$ 11

Figure S14 ^{1}H NMR (400 MHz) spectrum of compound **4a** in $CDCl_3$ 11
Figure S15 ^{1}H NMR (400 MHz) spectrum of compound **4b** in $CDCl_3$ 12
Figure S16 ^{1}H NMR (400 MHz) spectrum of compound **4c** in $CDCl_3$ 12
Figure S17 ^{1}H NMR (400 MHz) spectrum of compound **4d** in $CDCl_3$ 13
Figure S18 ^{1}H NMR (400 MHz) spectrum of compound **4e** in $CDCl_3$ 13
Figure S19 ^{1}H NMR (400 MHz) spectrum of compound **4f** in $CDCl_3$ 14
Figure S20 ^{1}H NMR (400 MHz) spectrum of compound **4g** in $CDCl_3$ 14
Figure S21 ^{1}H NMR (400 MHz) spectrum of compound **4h** in $CDCl_3$ 15
Figure S22 ^{1}H NMR (400 MHz) spectrum of compound **4i** in $CDCl_3$ 15
Figure S23 ^{1}H NMR (400 MHz) spectrum of compound **4j** in $CDCl_3$ 16
Figure S24 ^{1}H NMR (400 MHz) spectrum of compound **4k** in $CDCl_3$ 16
Figure S25 ^{1}H NMR (400 MHz) spectrum of compound **4l** in $CDCl_3$ 17
Figure S26 ^{1}H NMR (400 MHz) spectrum of compound **4m** in $CDCl_3$ 17
Figure S27 ^{1}H NMR (400 MHz) spectrum of compound **6a** in $CDCl_3$ 18
Figure S28 ^{1}H NMR (400 MHz) spectrum of compound **6b** in $CDCl_3$ 18
Figure S29 ^{1}H NMR (400 MHz) spectrum of compound **6c** in $CDCl_3$ 19
Figure S30 ^{1}H NMR (400 MHz) spectrum of compound **6d** in $CDCl_3$ 19
Figure S31 ^{1}H NMR (400 MHz) spectrum of compound **6e** in $CDCl_3$ 20
Figure S32 ^{1}H NMR (400 MHz) spectrum of compound **6f** in $CDCl_3$ 20
Figure S33 ^{1}H NMR (400 MHz) spectrum of compound **6g** in $CDCl_3$ 21
Figure S34 ^{1}H NMR (400 MHz) spectrum of compound **6h** in $CDCl_3$ 21
Figure S35 ^{1}H NMR (400 MHz) spectrum of compound **6i** in $CDCl_3$ 22
Figure S36 ^{1}H NMR (400 MHz) spectrum of compound **6j** in $CDCl_3$ 22
Figure S37 ^{1}H NMR (400 MHz) spectrum of compound **6k** in $CDCl_3$ 23
Figure S38 ^{1}H NMR (400 MHz) spectrum of compound **6l** in $CDCl_3$ 23
Figure S39 ^{1}H NMR (400 MHz) spectrum of compound **6m** in $CDCl_3$ 24
Figure S40 ^{1}H NMR (400 MHz) spectrum of compound **6A** in $CDCl_3$ 24
Figure S41 ^{1}H NMR (400 MHz) spectrum of compound **6B** in $CDCl_3$ 25
Figure S42 ^{1}H NMR (400 MHz) spectrum of compound **6C** in $CDCl_3$ 25
Figure S43 ^{1}H NMR (400 MHz) spectrum of compound **6D** in $CDCl_3$ 26
Figure S44 ^{1}H NMR (400 MHz) spectrum of compound **6E** in $CDCl_3$ 26

Figure S45 ^{1}H NMR (400 MHz) spectrum of compound **6F** in $CDCl_3$ 27
Figure S46 ^{1}H NMR (400 MHz) spectrum of compound **6G** in $CDCl_3$ 27
Figure S47 ^{1}H NMR (400 MHz) spectrum of compound **6H** in $CDCl_3$ 28
Figure S48 ^{1}H NMR (400 MHz) spectrum of compound **6I** in $CDCl_3$ 28
Figure S49 ^{1}H NMR (400 MHz) spectrum of compound **6J** in $CDCl_3$ 29
Figure S50 ^{1}H NMR (400 MHz) spectrum of compound **6K** in $CDCl_3$ 29
Figure S51 ^{1}H NMR (400 MHz) spectrum of compound **6L** in $CDCl_3$ 30
Figure S52 ^{1}H NMR (400 MHz) spectrum of compound **6M** in $CDCl_3$ 30
Figure S53 ^{1}H NMR (400 MHz) spectrum of compound **7a** in $CDCl_3$ 31
Figure S54 ^{13}C NMR (100 MHz) spectrum of compound **7a** in $CDCl_3$ 31
Figure S55 ^{1}H NMR (400 MHz) spectrum of compound **7b** in $CDCl_3$ 32
Figure S56 ^{13}C NMR (100 MHz) spectrum of compound **7b** in $CDCl_3$ 32
Figure S57 ^{1}H NMR (400 MHz) spectrum of compound **7c** in CD_3OD 33
Figure S58 ^{13}C NMR (100 MHz) spectrum of compound **7c** in CD_3OD 33
Figure S59 ^{1}H NMR (400 MHz) spectrum of compound **7d** in $CDCl_3$ 34
Figure S60 ^{13}C NMR (100 MHz) spectrum of compound **7d** in $CDCl_3$ 34
Figure S61 ^{1}H NMR (400 MHz) spectrum of compound **7e** in $CDCl_3$ 35
Figure S62 ^{13}C NMR (100 MHz) spectrum of compound **7e** in $CDCl_3$ 35
Figure S63 ^{1}H NMR (400 MHz) spectrum of compound **7f** in $CDCl_3$ 36
Figure S64 ^{13}C NMR (100 MHz) spectrum of compound **7f** in $CDCl_3$ 36
Figure S65 ^{1}H NMR (400 MHz) spectrum of compound **7g** in $CDCl_3$ 37
Figure S66 ^{13}C NMR (100 MHz) spectrum of compound **7g** in $CDCl_3$ 37
Figure S67 ^{1}H NMR (600 MHz) spectrum of compound **7h** in $CDCl_3$ 38
Figure S68 ^{13}C NMR (100 MHz) spectrum of compound **7h** in $CDCl_3$ 38
Figure S69 ^{1}H NMR (400 MHz) spectrum of compound **7i** in $CDCl_3$ 39
Figure S70 ^{13}C NMR (150 MHz) spectrum of compound **7i** in $CDCl_3$ 39
Figure S71 ^{1}H NMR (600 MHz) spectrum of compound **7j** in $CDCl_3$ 40
Figure S72 ^{13}C NMR (150 MHz) spectrum of compound **7j** in $CDCl_3$ 40
Figure S73 ^{1}H NMR (400 MHz) spectrum of compound **7k** in $CDCl_3$ 41
Figure S74 ^{13}C NMR (100 MHz) spectrum of compound **7k** in $CDCl_3$ 41
Figure S75 ^{1}H NMR (400 MHz) spectrum of compound **7l** in $CDCl_3$ 42

Figure S76 ^{13}C NMR (100 MHz) spectrum of compound **7l** in $CDCl_3$ 42
Figure S77 ^{1}H NMR (600 MHz) spectrum of compound **7m** in $CDCl_3$ 43
Figure S78 ^{13}C NMR (150 MHz) spectrum of compound **7m** in $CDCl_3$ 43
Figure S79 ^{1}H NMR (400 MHz) spectrum of compound **7A** in $CDCl_3$ 44
Figure S80 ^{13}C NMR (100 MHz) spectrum of compound **7A** in $CDCl_3$ 44
Figure S81 ^{1}H NMR (400 MHz) spectrum of compound **7B** in $CDCl_3$ 45
Figure S82 ^{13}C NMR (100 MHz) spectrum of compound **7B** in $CDCl_3$ 45
Figure S83 ^{1}H NMR (400 MHz) spectrum of compound **7C** in $CDCl_3$ 46
Figure S84 ^{13}C NMR (100 MHz) spectrum of compound **7C** in $CDCl_3$ 46
Figure S85 ^{1}H NMR (400 MHz) spectrum of compound **7D** in $CDCl_3$ 47
Figure S86 ^{13}C NMR (100 MHz) spectrum of compound **7D** in $CDCl_3$ 47
Figure S87 ^{1}H NMR (400 MHz) spectrum of compound **7E** in $CDCl_3$ 48
Figure S88 ^{13}C NMR (100 MHz) spectrum of compound **7E** in $CDCl_3$ 48
Figure S89 ^{1}H NMR (600 MHz) spectrum of compound **7F** in $CDCl_3$ 49
Figure S90 ^{13}C NMR (150 MHz) spectrum of compound **7F** in $CDCl_3$ 49
Figure S91 ^{1}H NMR (400 MHz) spectrum of compound **7G** in $CDCl_3$ 50
Figure S92 ^{13}C NMR (100 MHz) spectrum of compound **7G** in $CDCl_3$ 50
Figure S93 ^{1}H NMR (400 MHz) spectrum of compound **7H** in $CDCl_3$ 51
Figure S94 ^{13}C NMR (100 MHz) spectrum of compound **7H** in $CDCl_3$ 51
Figure S95 ^{1}H NMR (400 MHz) spectrum of compound **7I** in $CDCl_3$ 52
Figure S96 ^{13}C NMR (100 MHz) spectrum of compound **7I** in $CDCl_3$ 52
Figure S97 ^{1}H NMR (400 MHz) spectrum of compound **7J** in $CDCl_3$ 53
Figure S98 ^{13}C NMR (100 MHz) spectrum of compound **7J** in $CDCl_3$ 53
Figure S99 ^{1}H NMR (400 MHz) spectrum of compound **7K** in $CDCl_3$ 54
Figure S100 ^{13}C NMR (100 MHz) spectrum of compound **7K** in $CDCl_3$ 54
Figure S101 ^{1}H NMR (400 MHz) spectrum of compound **7L** in $CDCl_3$ 55
Figure S102 ^{13}C NMR (100 MHz) spectrum of compound **7L** in $CDCl_3$ 55
Figure S103 ^{1}H NMR (400 MHz) spectrum of compound **7M** in $CDCl_3$ 56
Figure S104 ^{13}C NMR (100 MHz) spectrum of compound **7M** in $CDCl_3$ 56

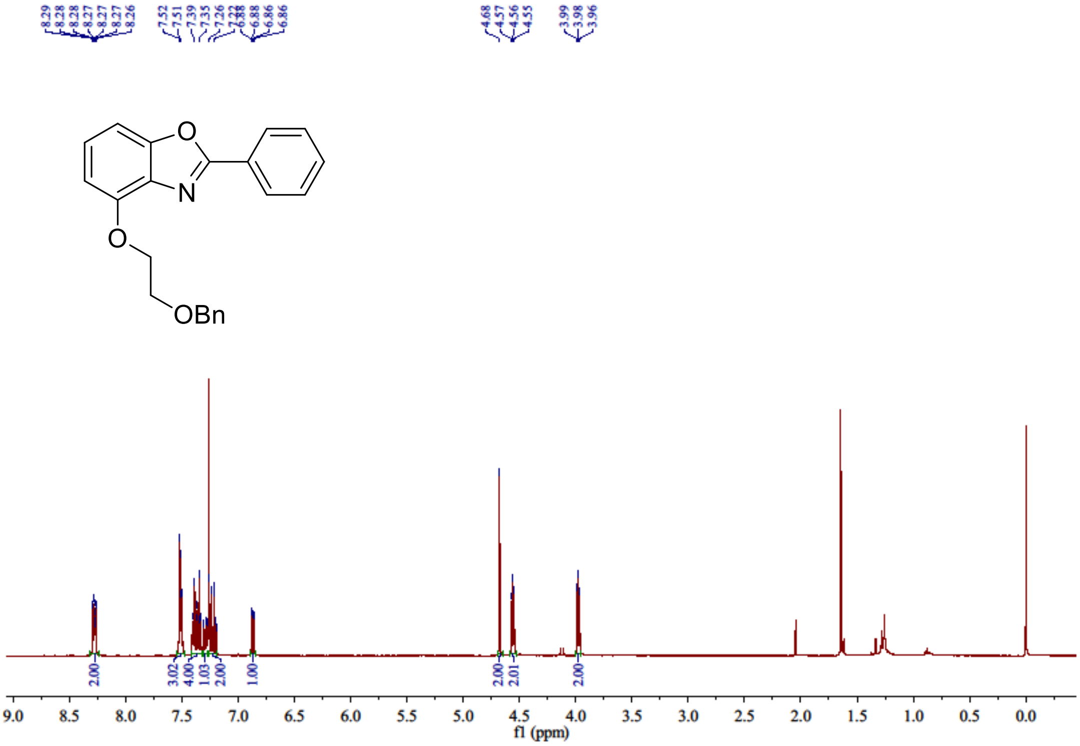

Figure S1 ^{1}H NMR (400 MHz) spectrum of compound **3a** in $CDCl_3$

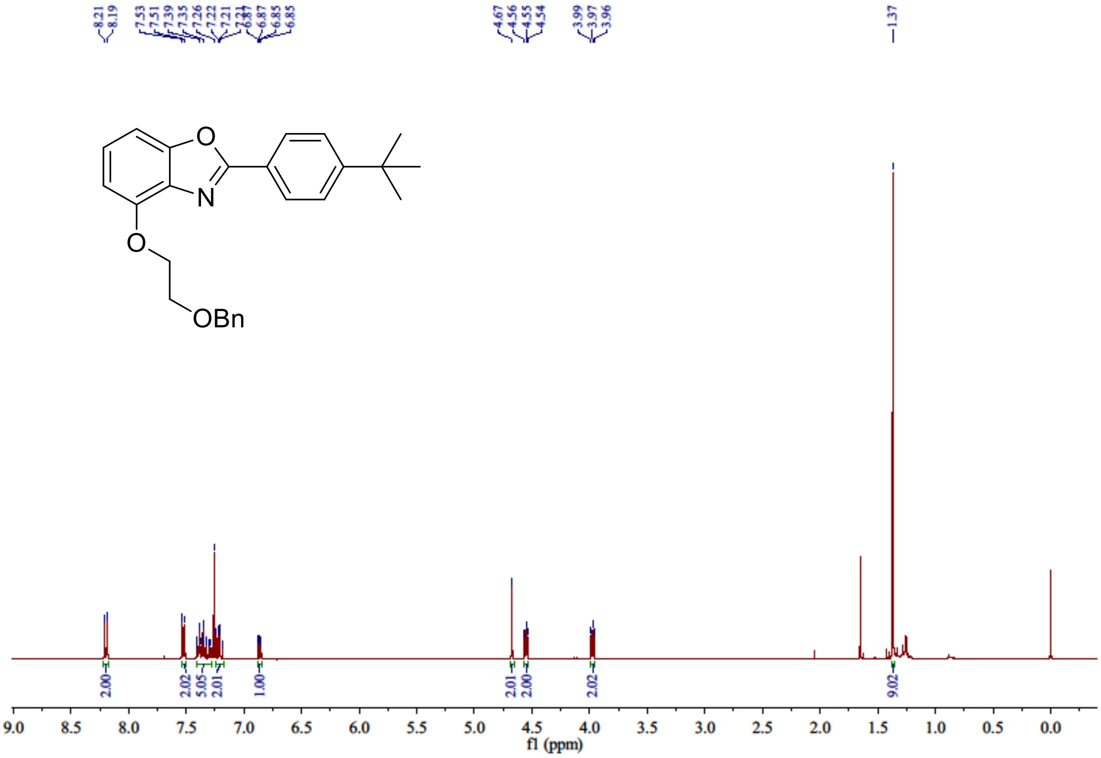

Figure S2 ^{1}H NMR (400 MHz) spectrum of compound **3b** in $CDCl_3$

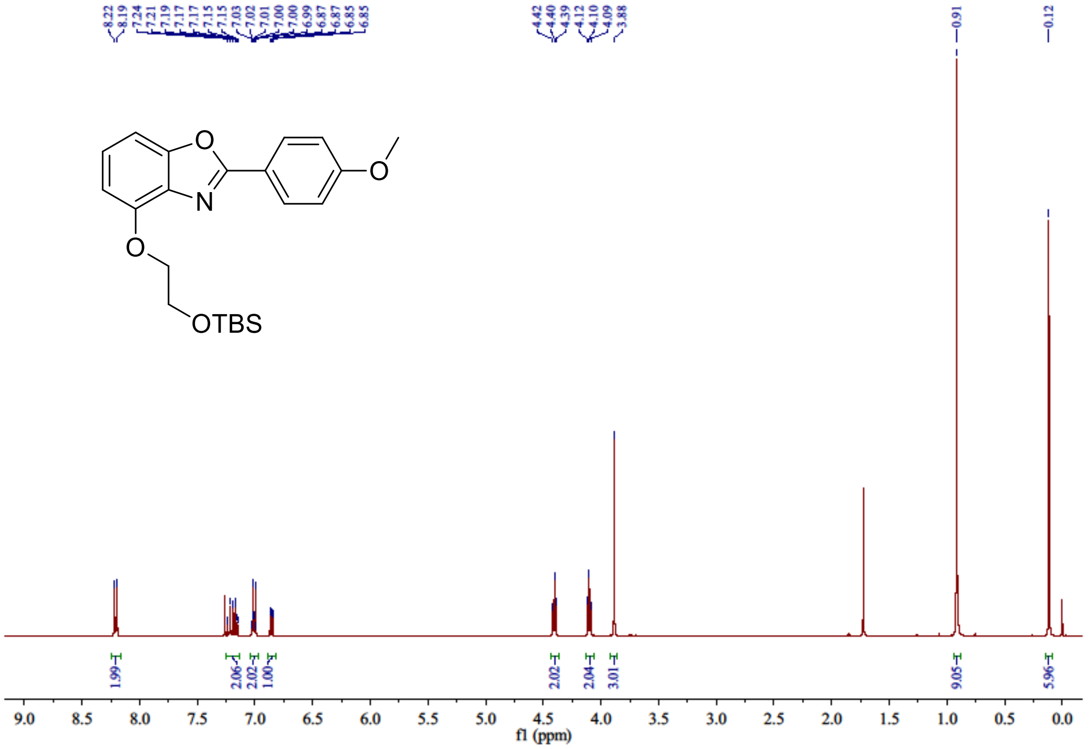

Figure S3 ^{1}H NMR (400 MHz) spectrum of compound **3c** in $CDCl_3$

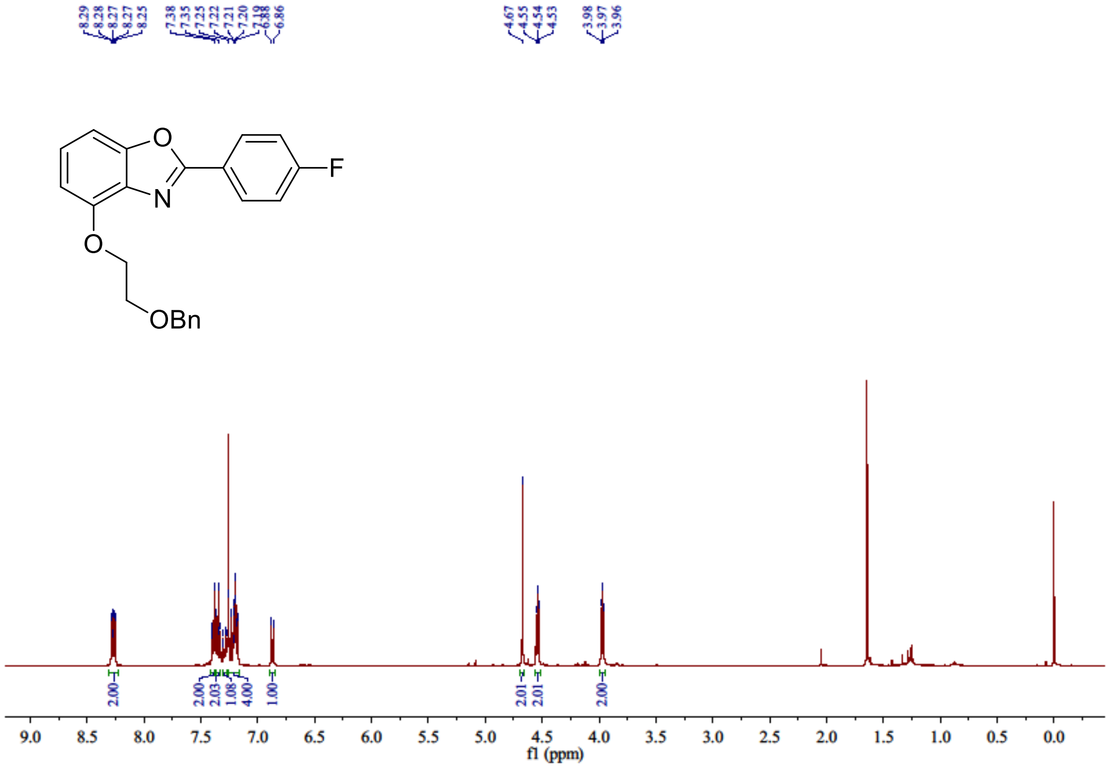

Figure S4 ^{1}H NMR (400 MHz) spectrum of compound **3d** in $CDCl_3$

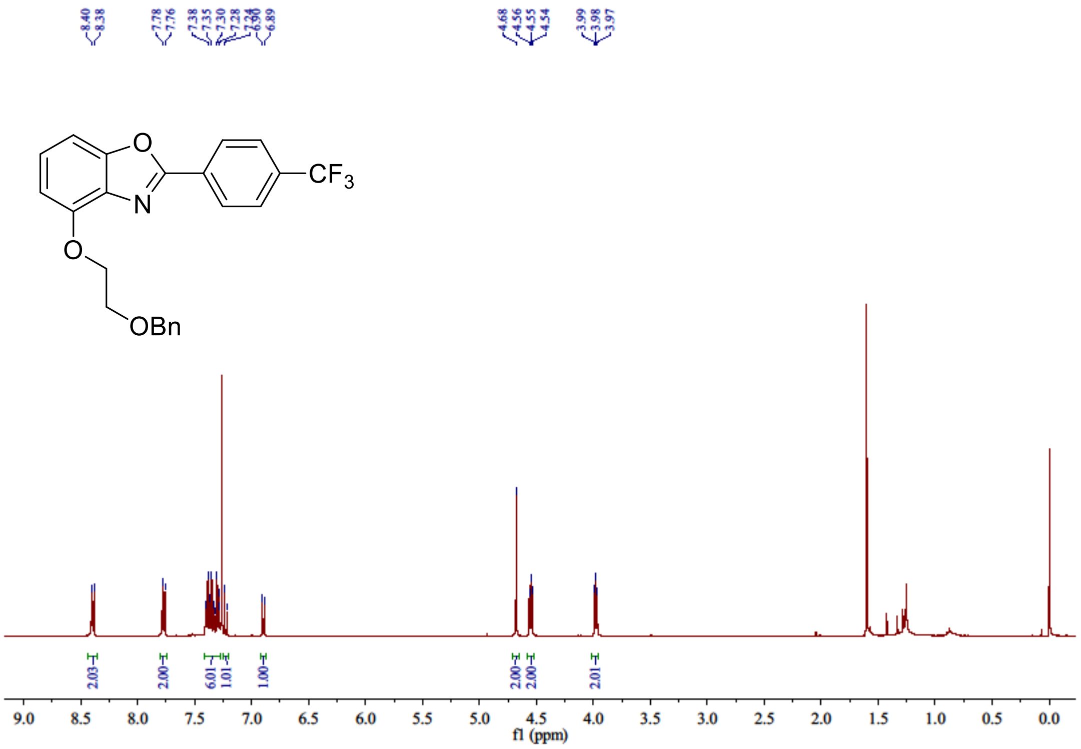

Figure S5 ^{1}H NMR (400 MHz) spectrum of compound **3e** in $CDCl_3$

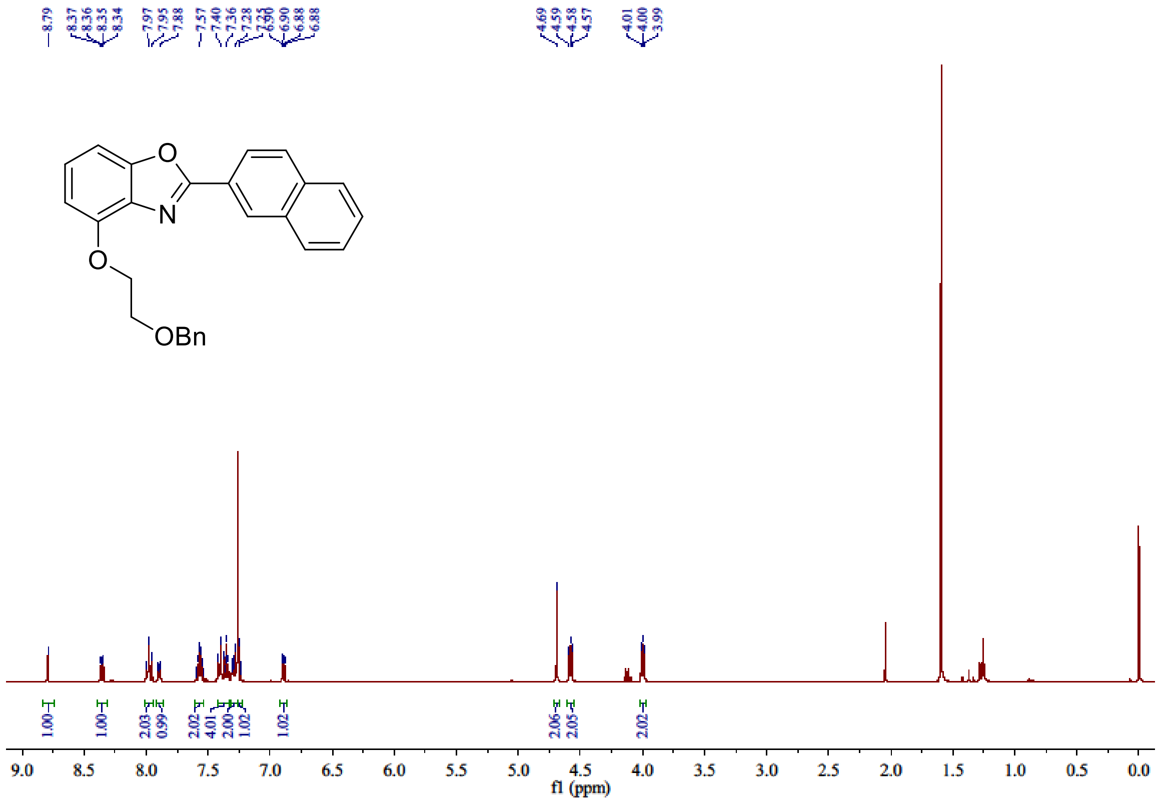

Figure S6 ^{1}H NMR (400 MHz) spectrum of compound **3f** in $CDCl_3$

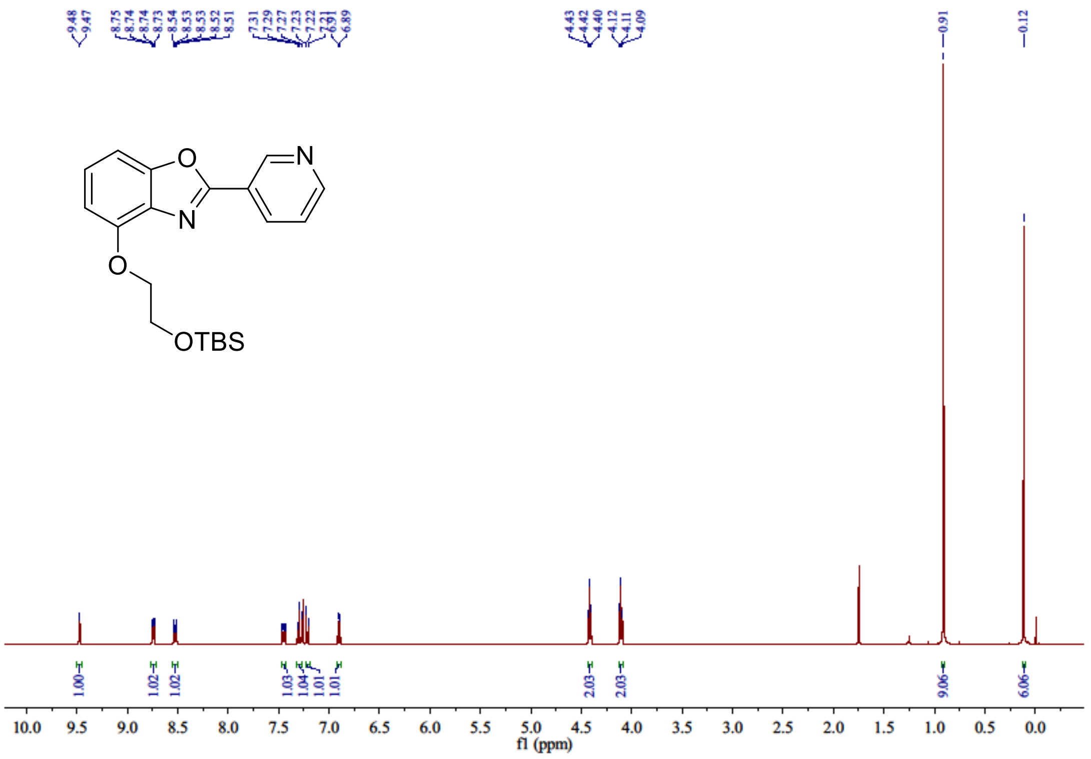

Figure S7 ^{1}H NMR (400 MHz) spectrum of compound **3g** in $CDCl_3$

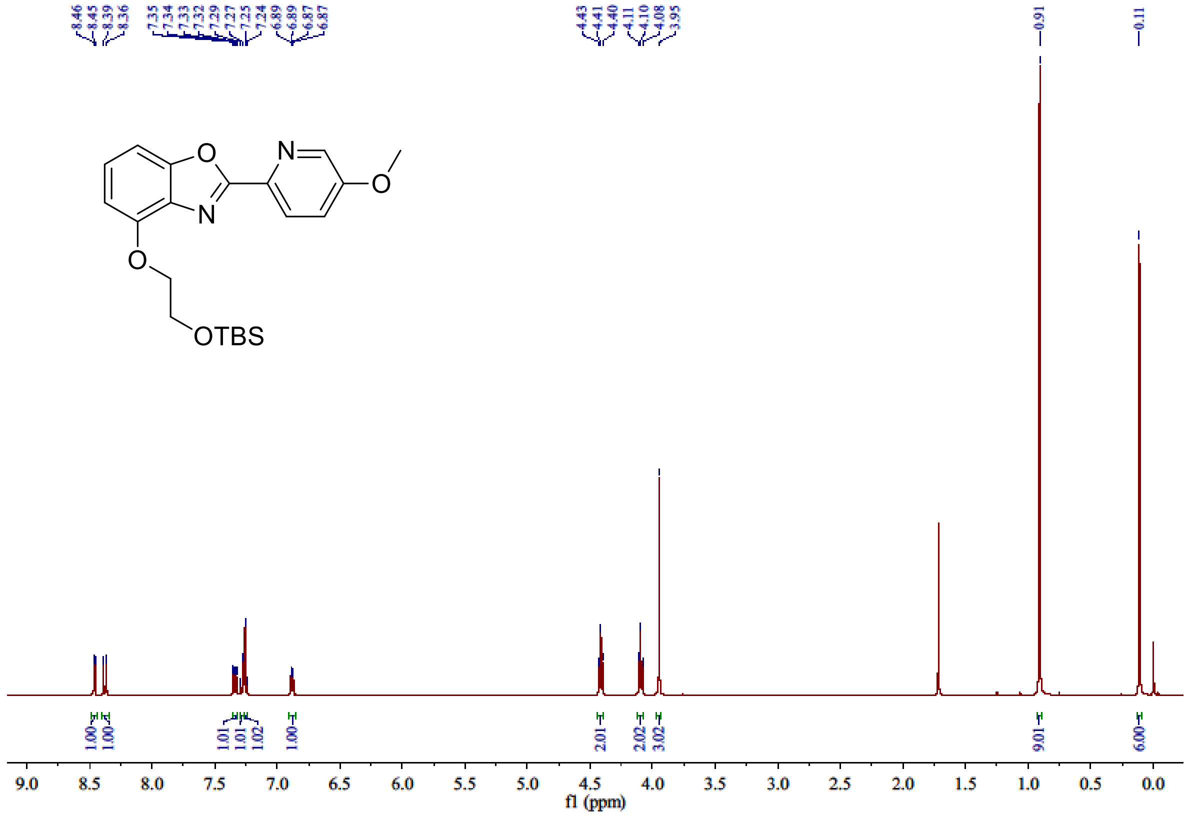

Figure S8 ^{1}H NMR (400 MHz) spectrum of compound **3h** in $CDCl_3$

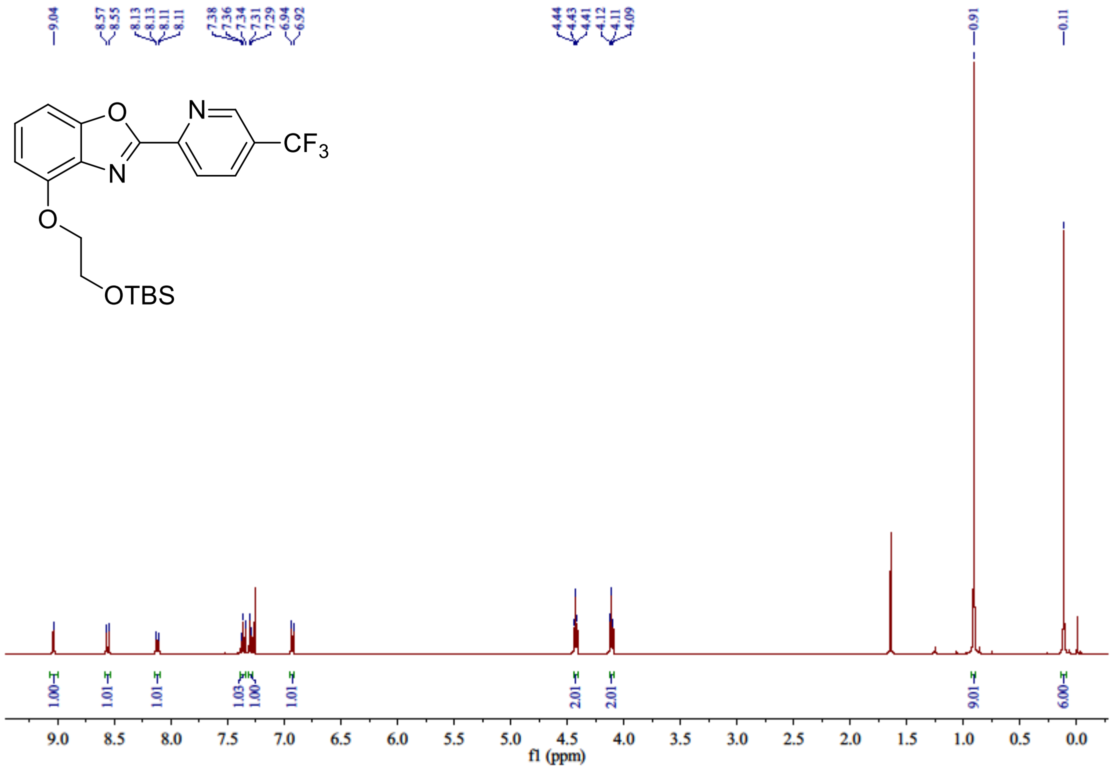

Figure S9 ^{1}H NMR (400 MHz) spectrum of compound **3i** in $CDCl_3$

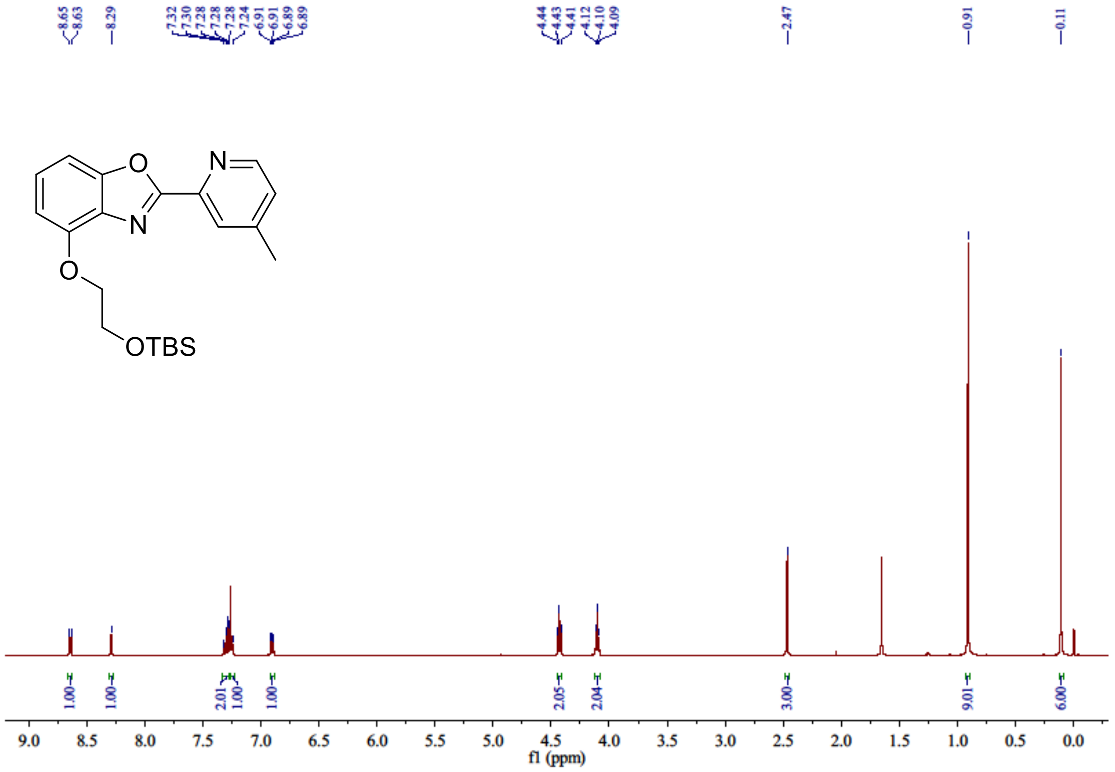

Figure S10 ^{1}H NMR (400 MHz) spectrum of compound **3j** in $CDCl_3$

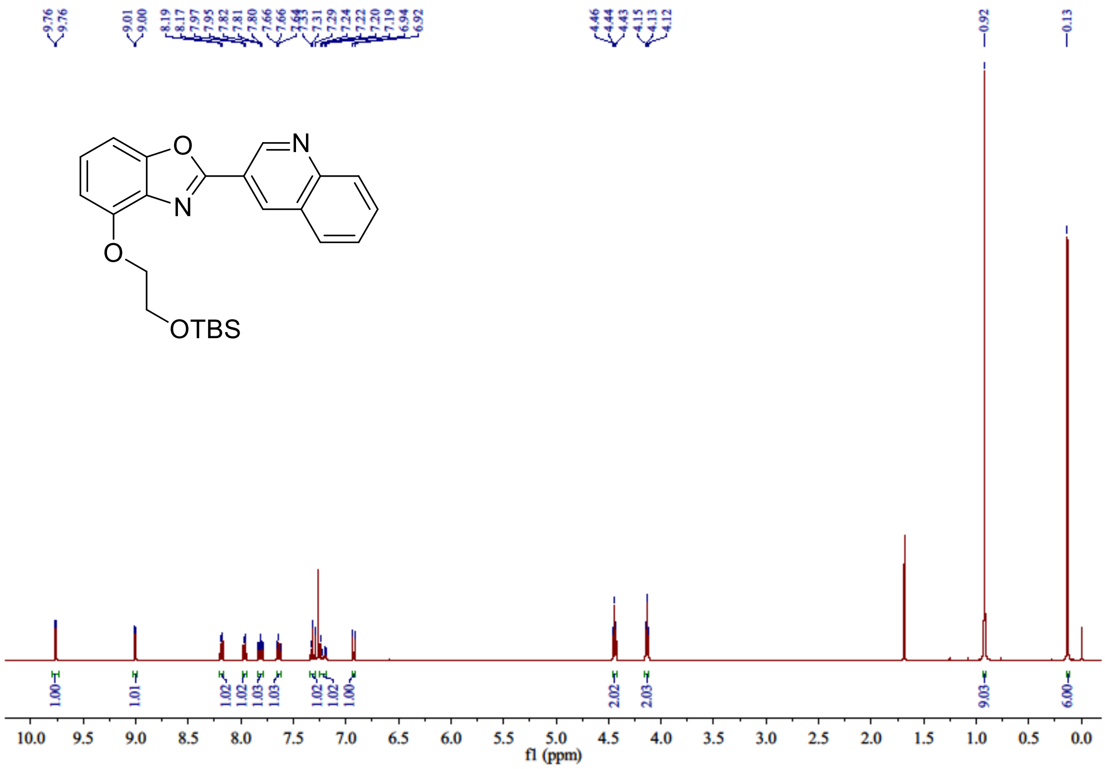

Figure S11 ^{1}H NMR (400 MHz) spectrum of compound **3k** in $CDCl_3$

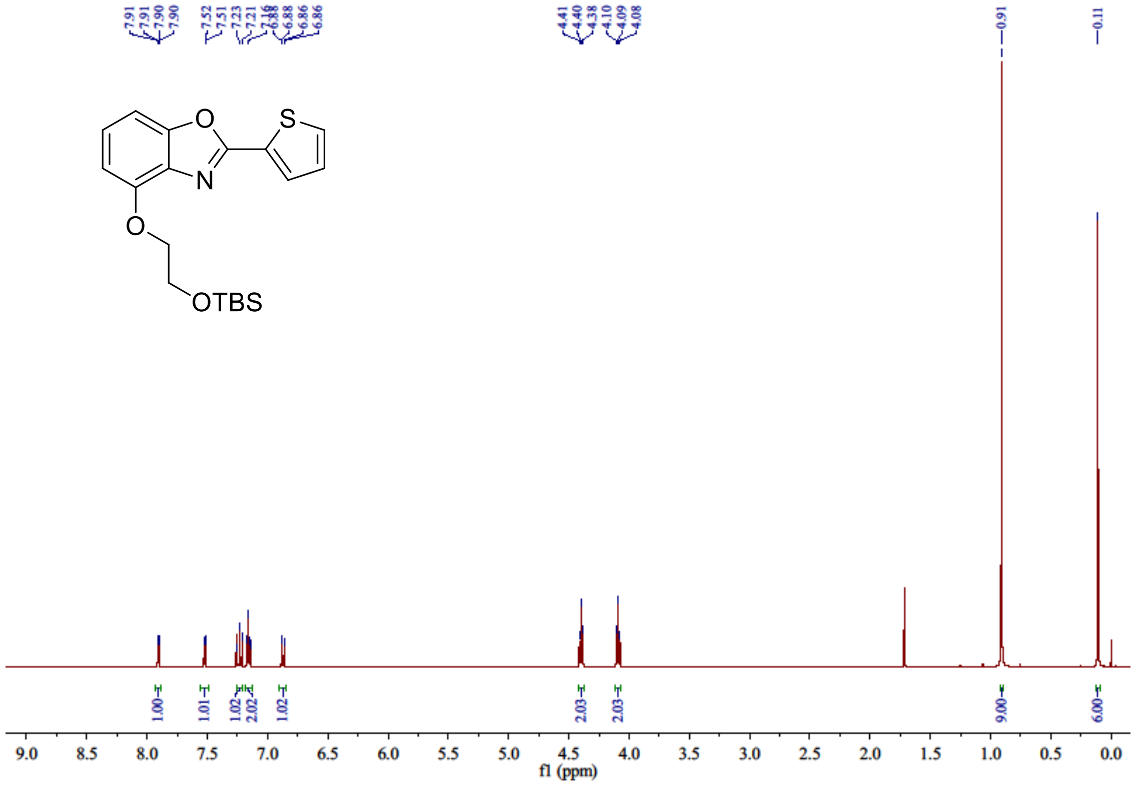

Figure S12 ^{1}H NMR (400 MHz) spectrum of compound **3l** in $CDCl_3$

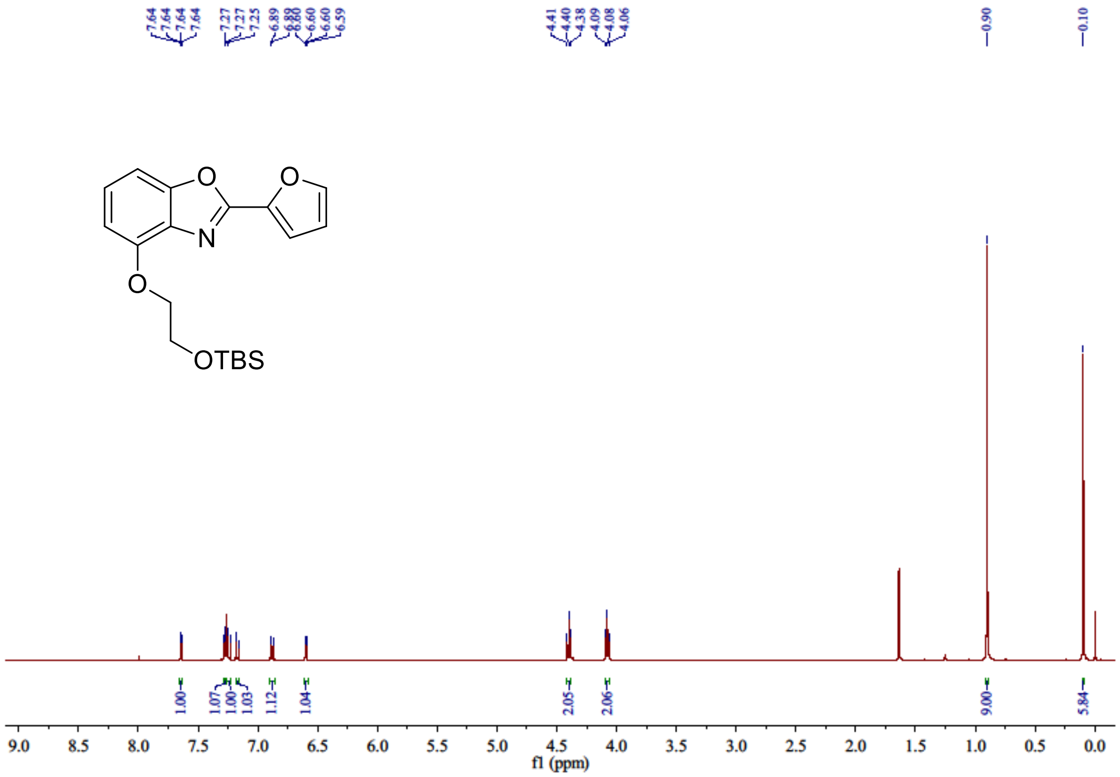

Figure S13 ^{1}H NMR (400 MHz) spectrum of compound **3m** in $CDCl_3$

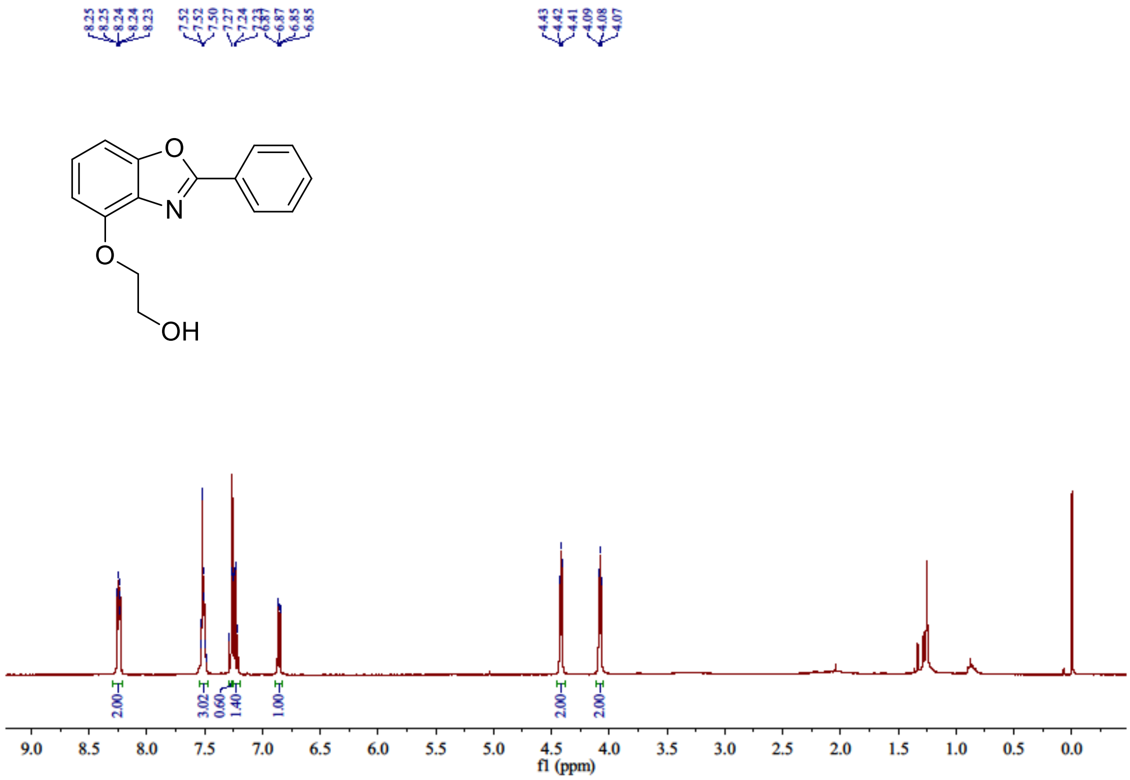

Figure S14 ^{1}H NMR (400 MHz) spectrum of compound **4a** in $CDCl_3$

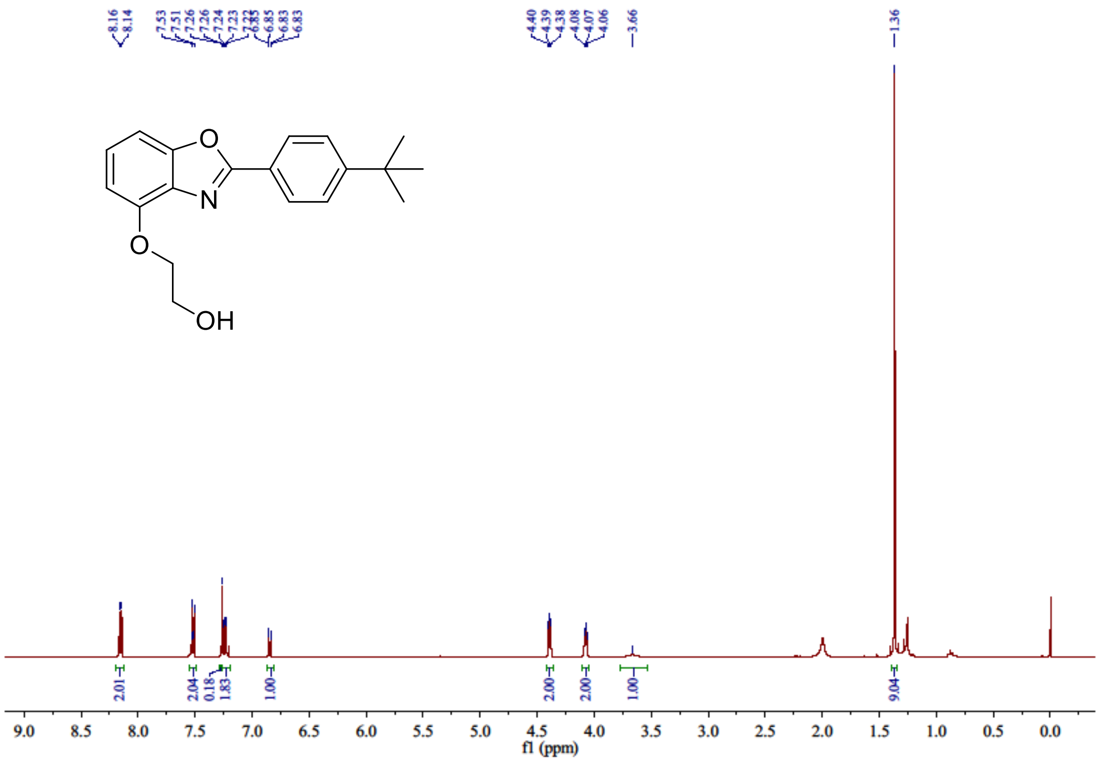

Figure S15 ^{1}H NMR (400 MHz) spectrum of compound **4b** in $CDCl_3$

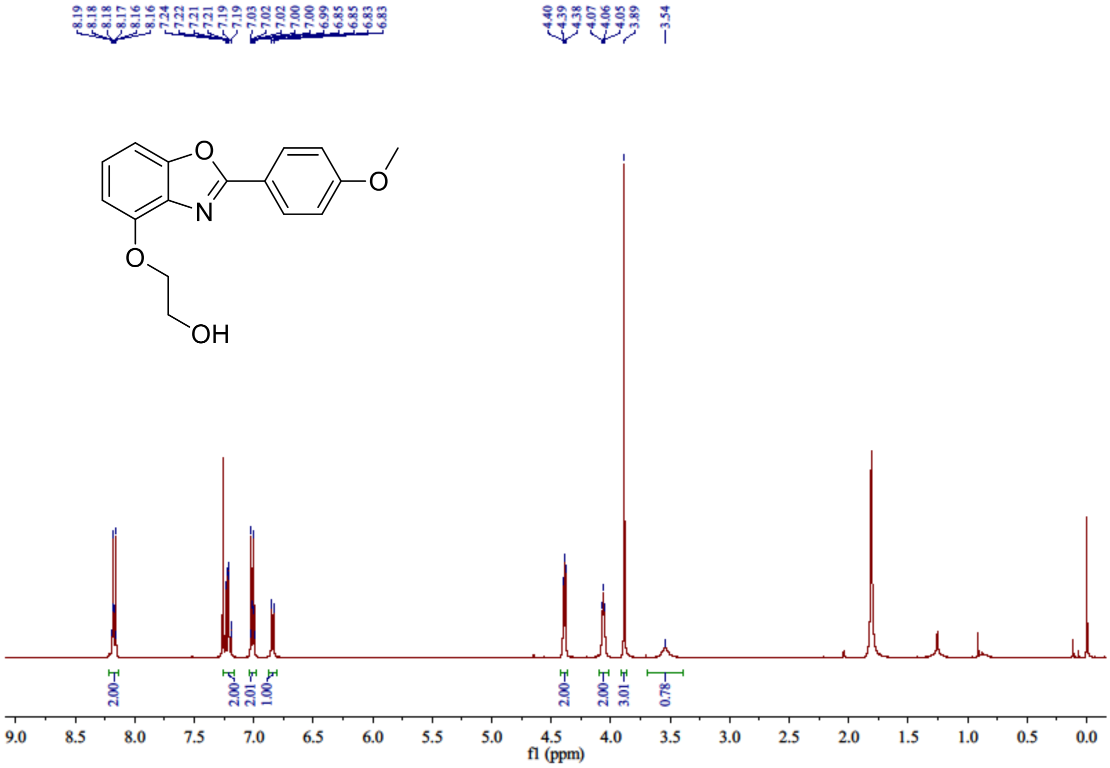

Figure S16 ^{1}H NMR (400 MHz) spectrum of compound **4c** in $CDCl_3$

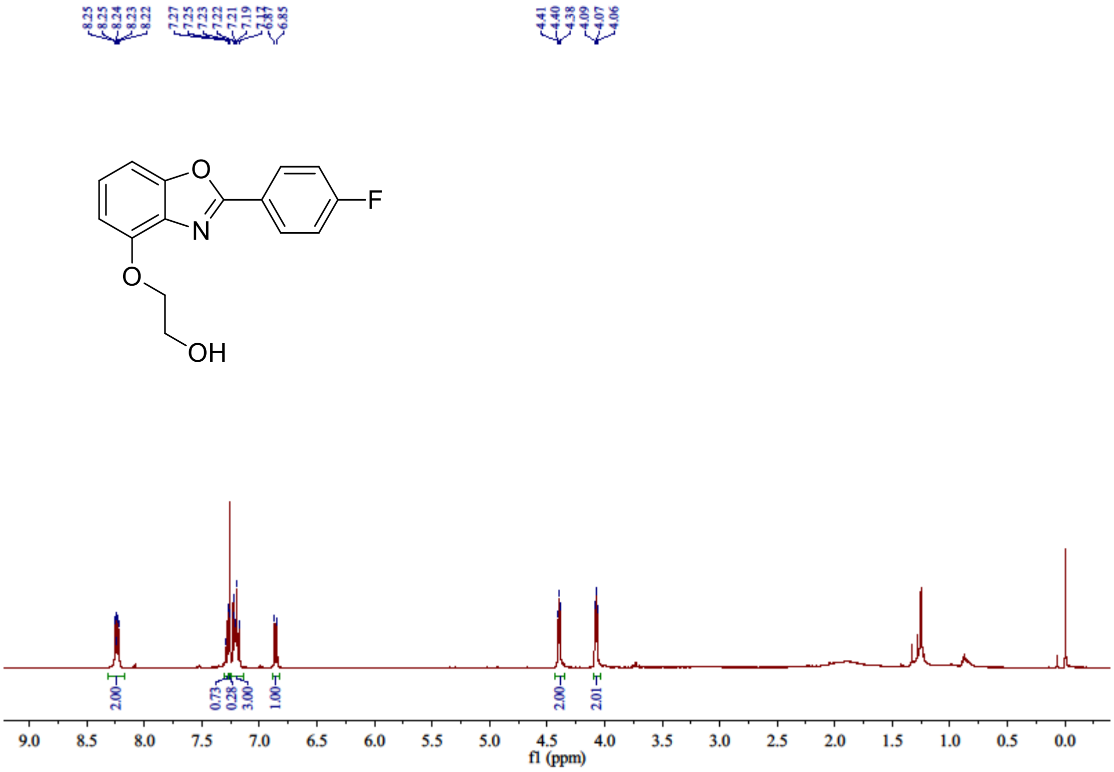

Figure S17 ^{1}H NMR (400 MHz) spectrum of compound **4d** in $CDCl_3$

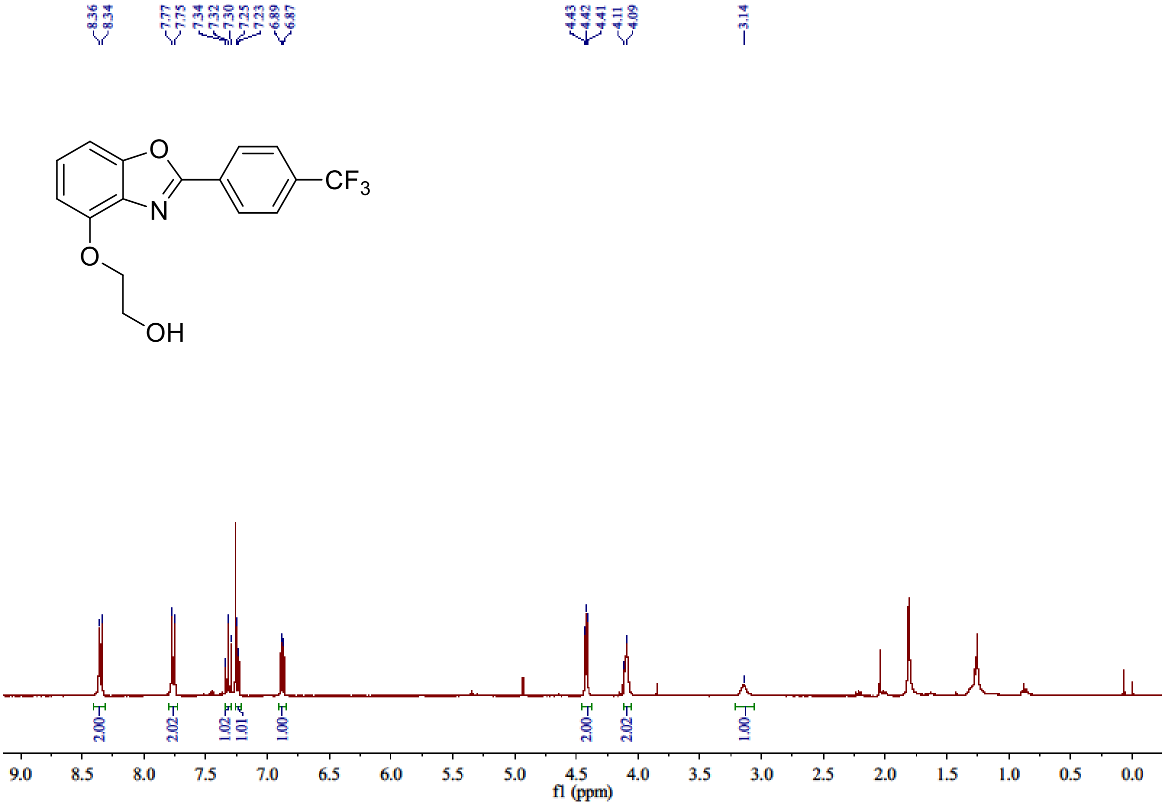

Figure S18 ^{1}H NMR (400 MHz) spectrum of compound **4e** in $CDCl_3$

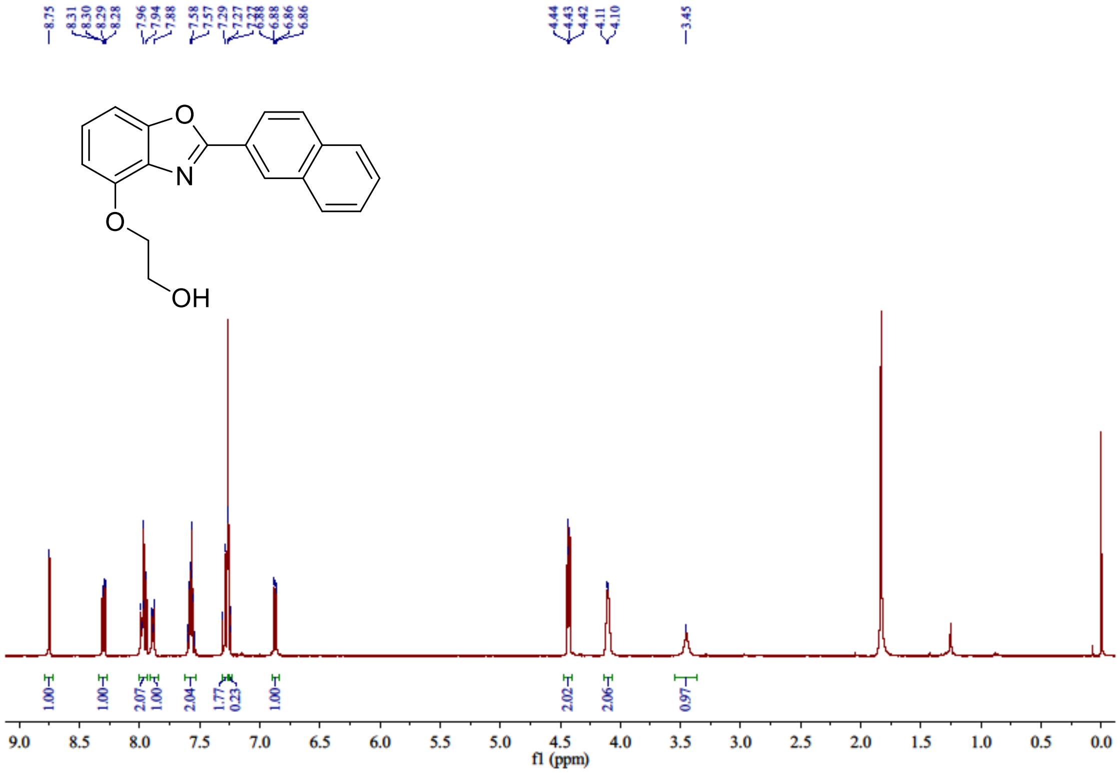

Figure S19 ^{1}H NMR (400 MHz) spectrum of compound **4f** in $CDCl_3$

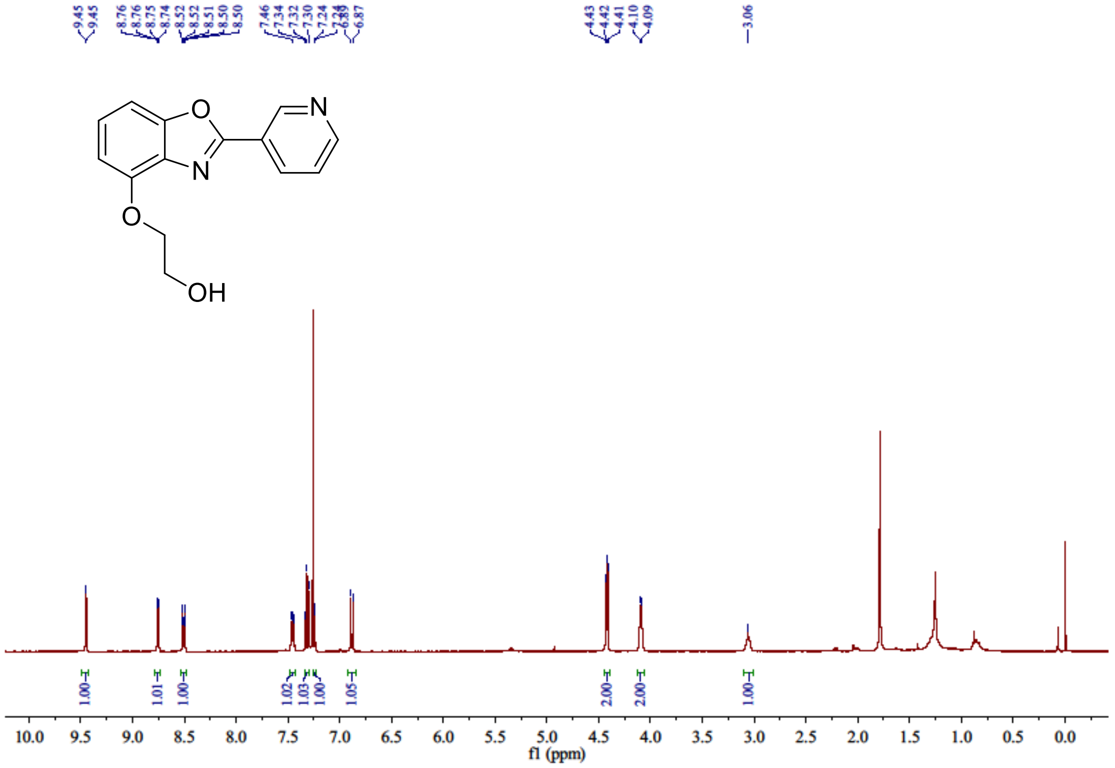

Figure S20 ^{1}H NMR (400 MHz) spectrum of compound **4g** in $CDCl_3$

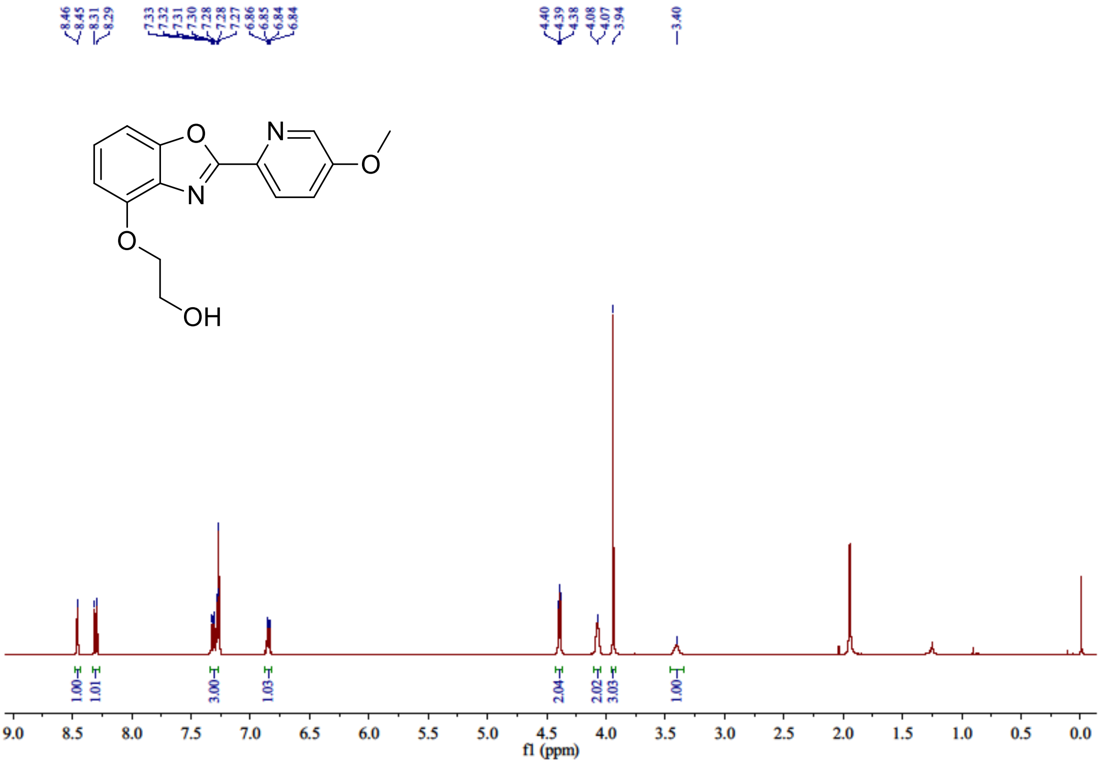

Figure S21 ^{1}H NMR (400 MHz) spectrum of compound **4h** in $CDCl_3$

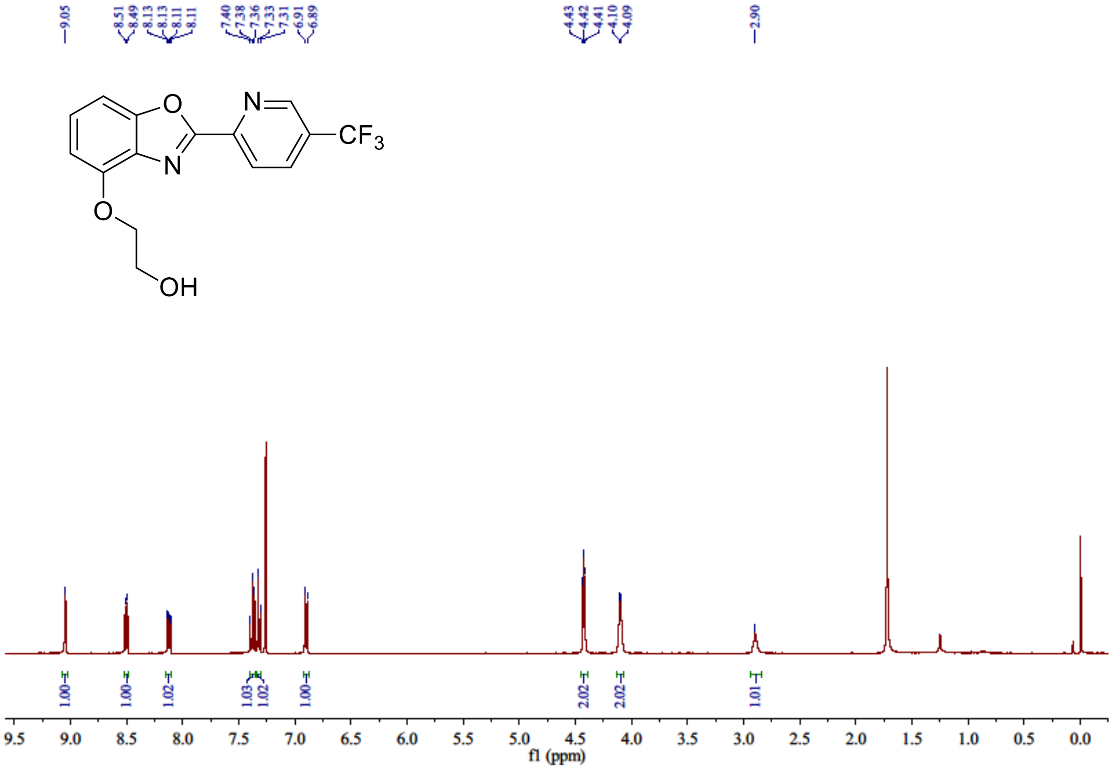

Figure S22 ^{1}H NMR (400 MHz) spectrum of compound **4i** in $CDCl_3$

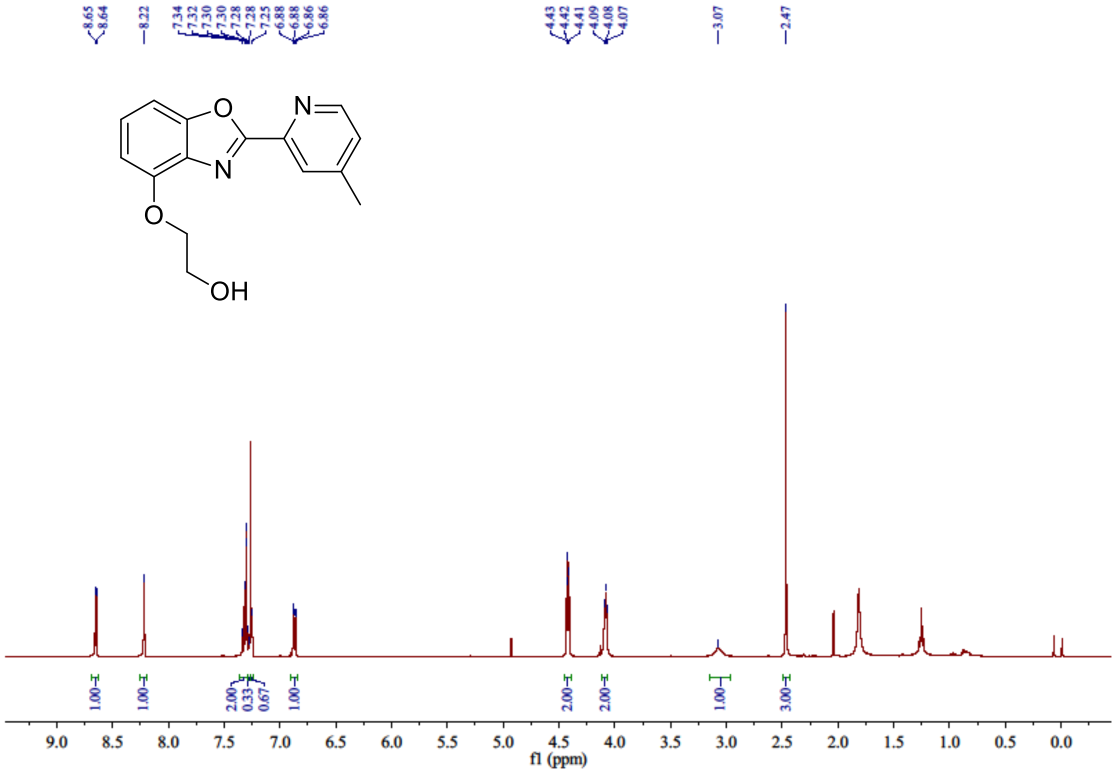

Figure S23 ^{1}H NMR (400 MHz) spectrum of compound **4j** in $CDCl_3$

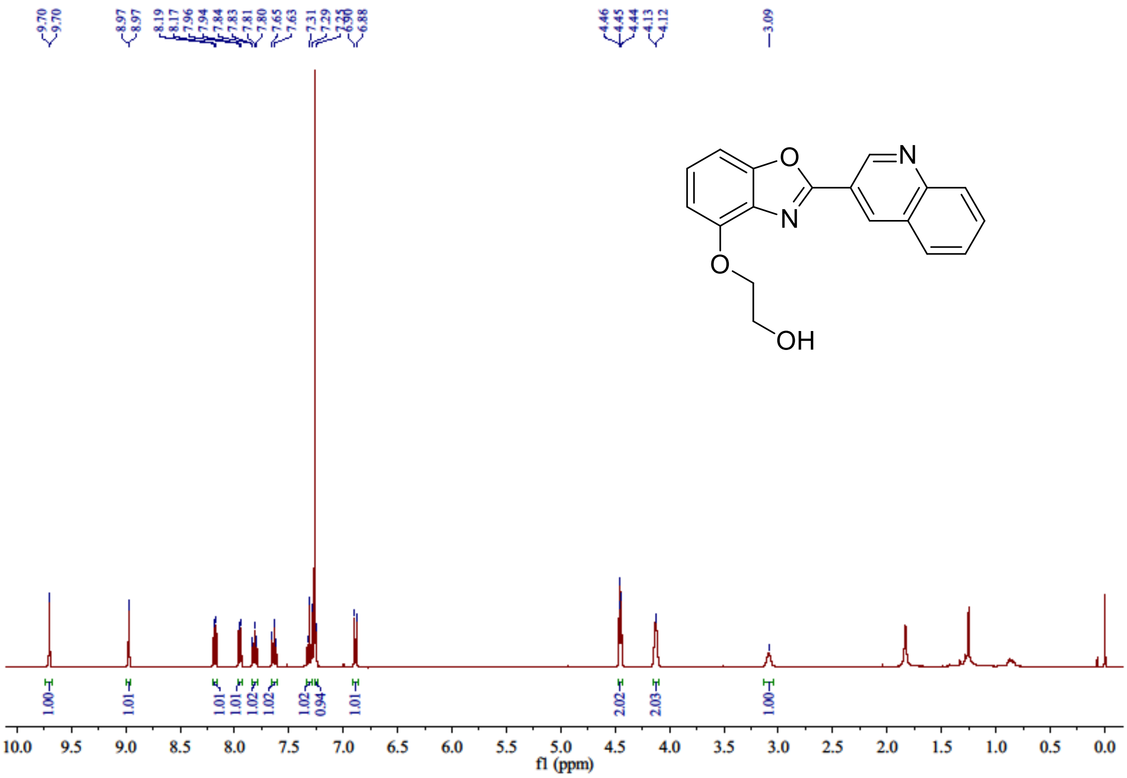

Figure S24 ^{1}H NMR (400 MHz) spectrum of compound **4k** in $CDCl_3$

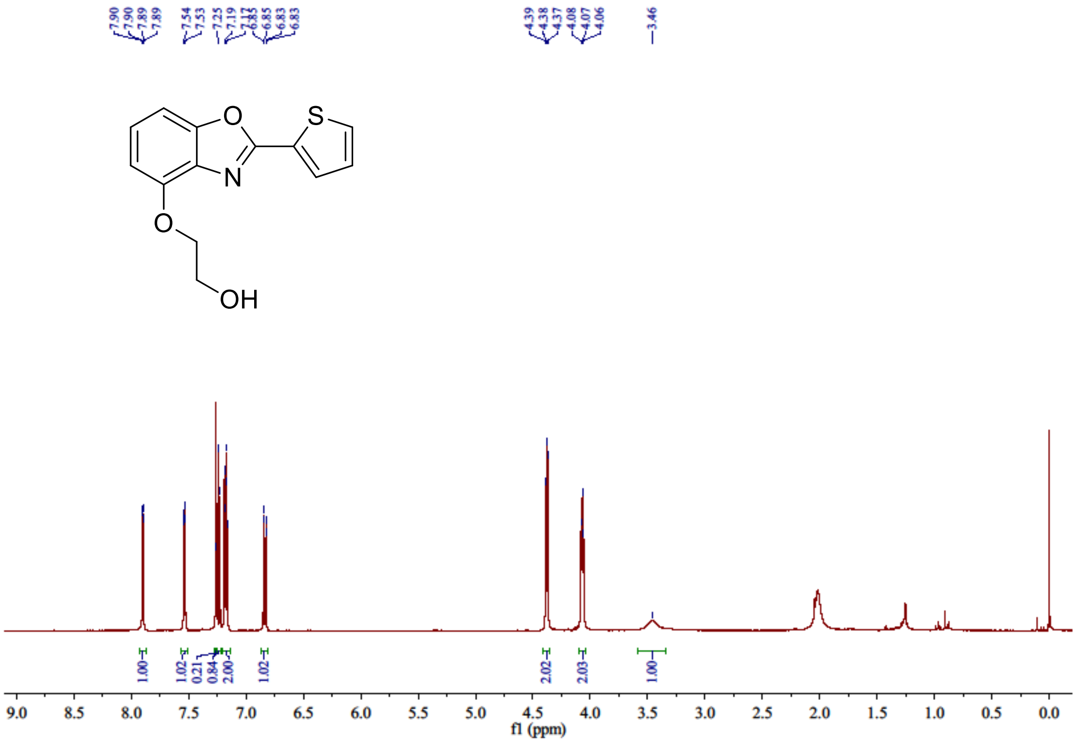

Figure S25 ^{1}H NMR (400 MHz) spectrum of compound **4l** in $CDCl_3$

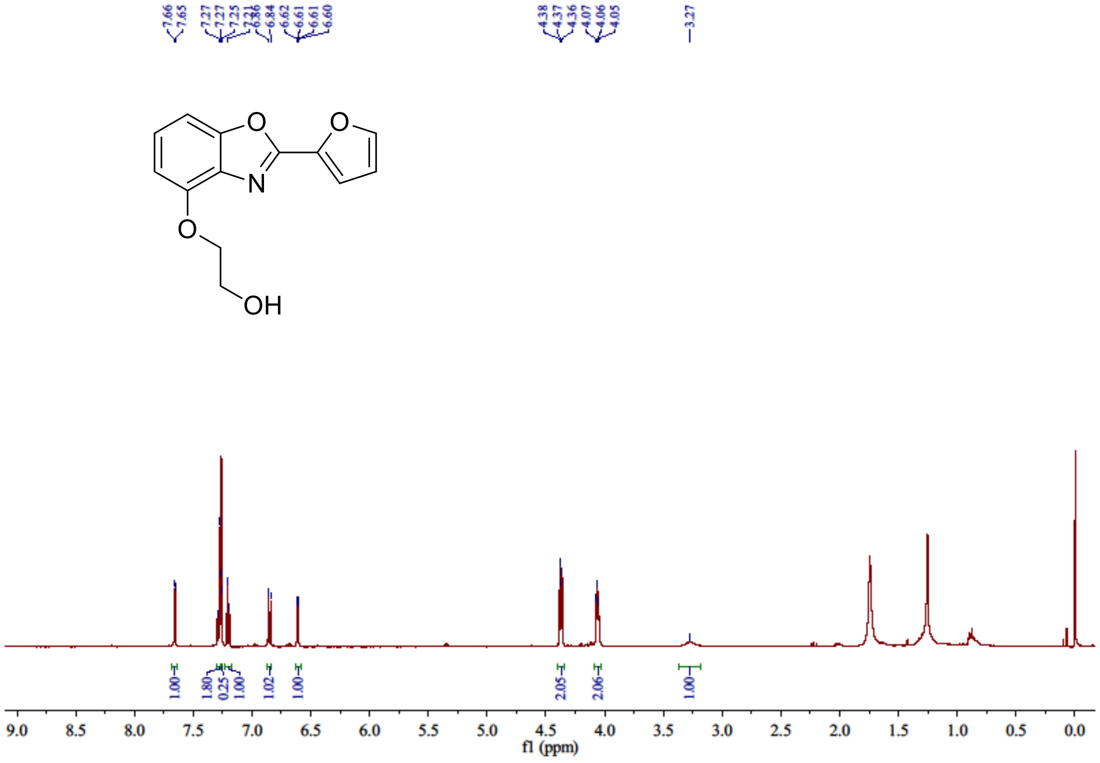

Figure S26 ^{1}H NMR (400 MHz) spectrum of compound **4m** in $CDCl_3$

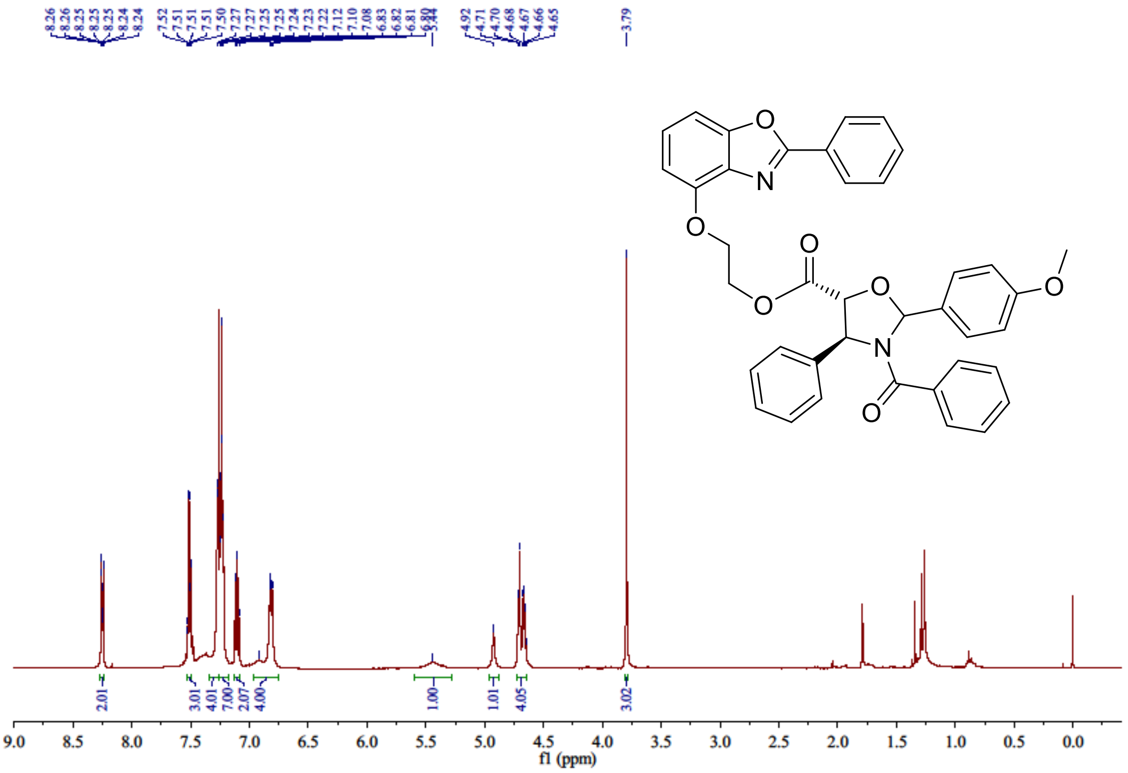

Figure S27 ^{1}H NMR (400 MHz) spectrum of compound **6a** in $CDCl_3$

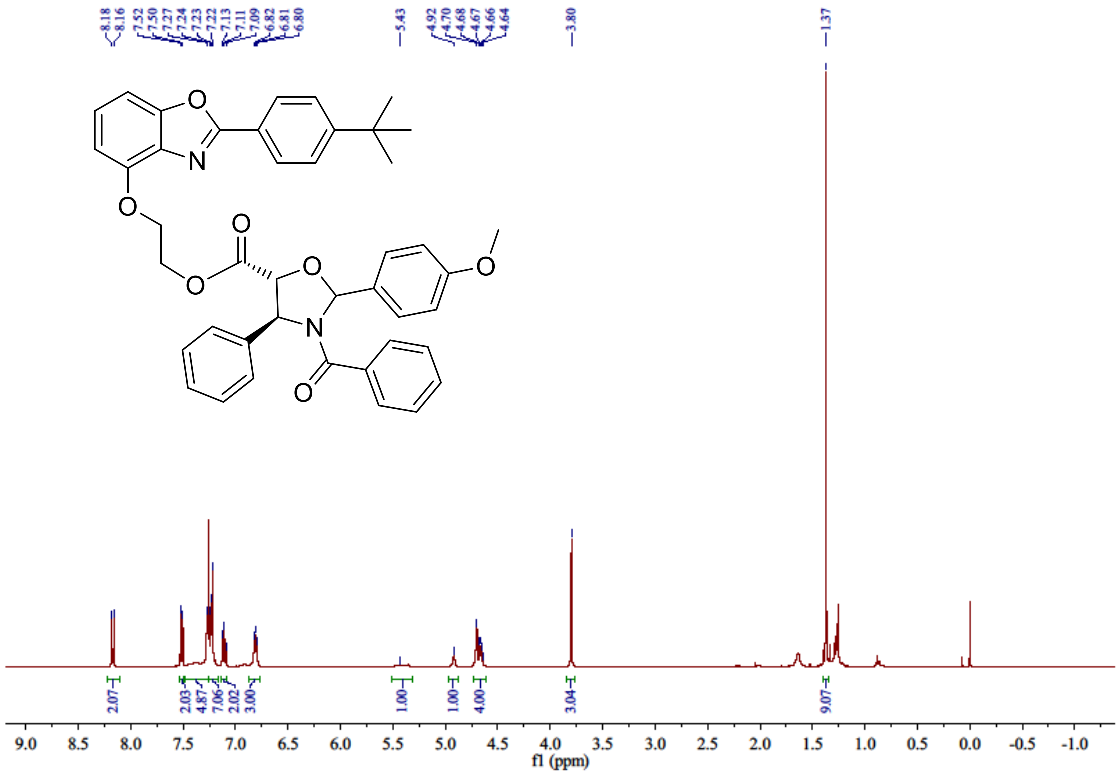

Figure S28 ^{1}H NMR (400 MHz) spectrum of compound **6b** in $CDCl_3$

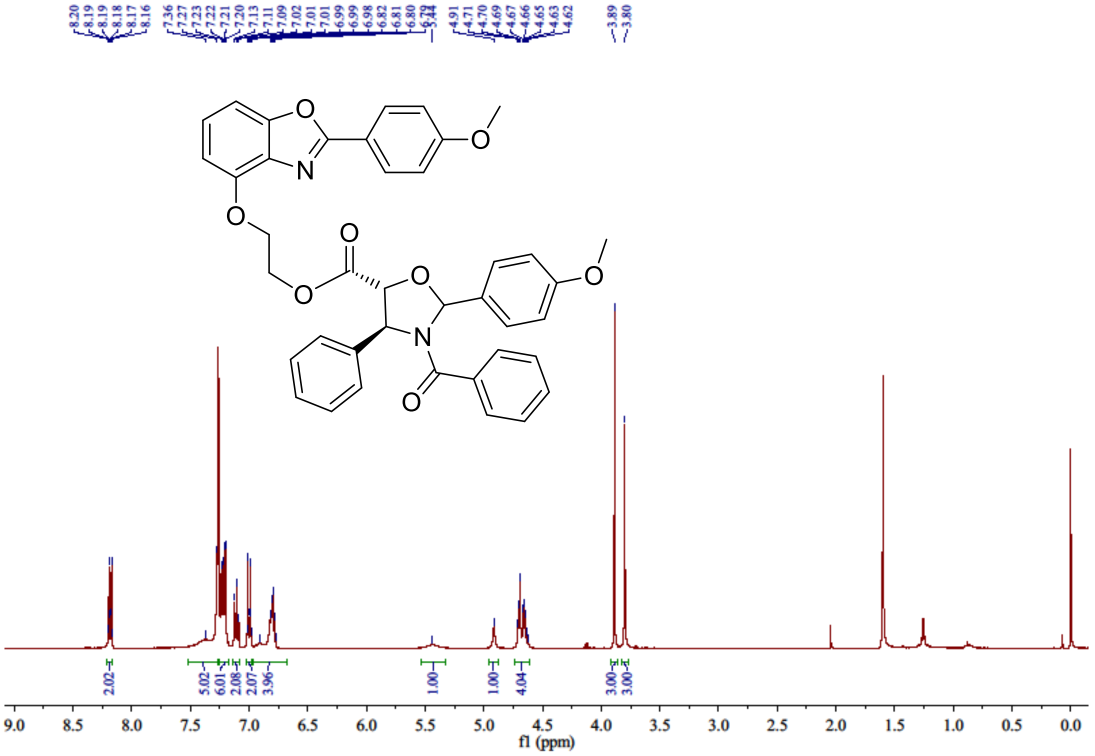

Figure S29 ^{1}H NMR (400 MHz) spectrum of compound **6c** in $CDCl_3$

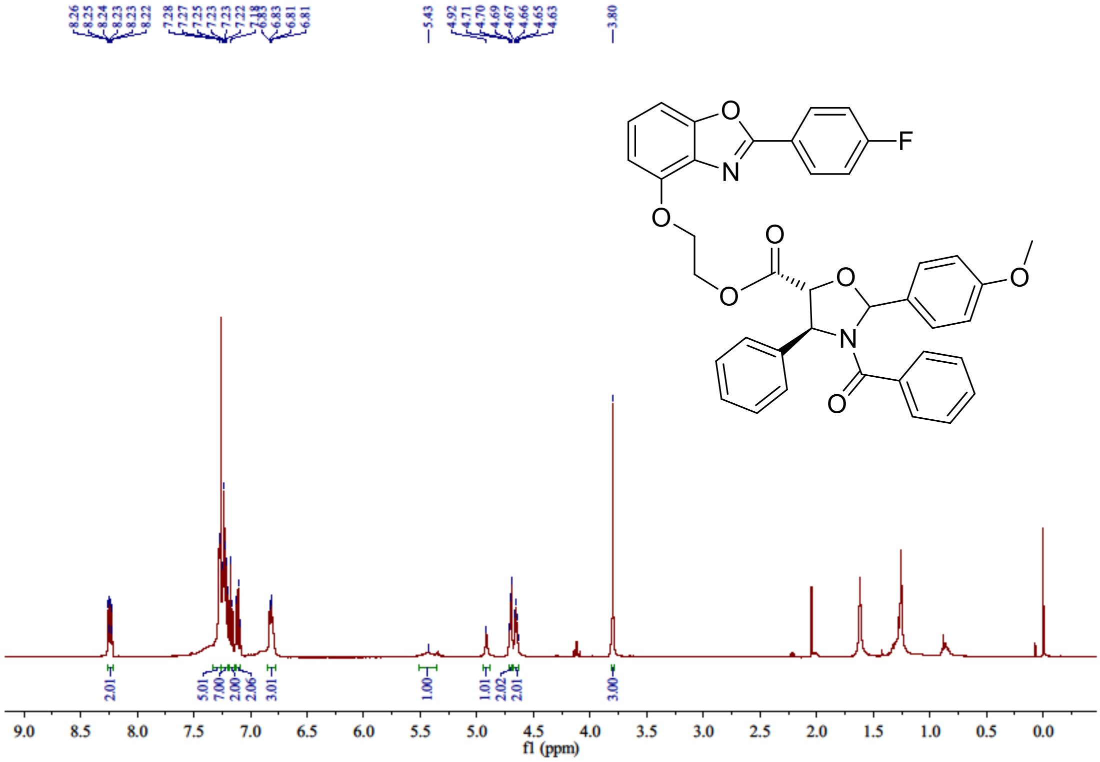

Figure S30 ^{1}H NMR (400 MHz) spectrum of compound **6d** in $CDCl_3$

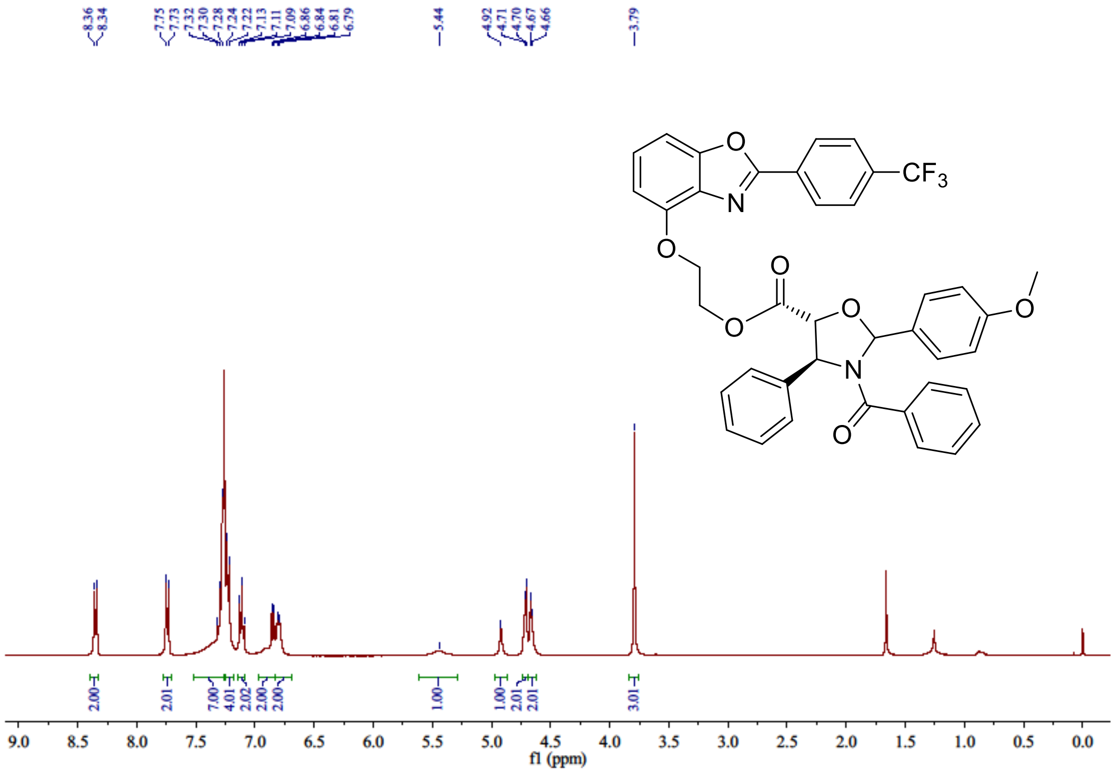

Figure S31 ^{1}H NMR (400 MHz) spectrum of compound **6e** in $CDCl_3$

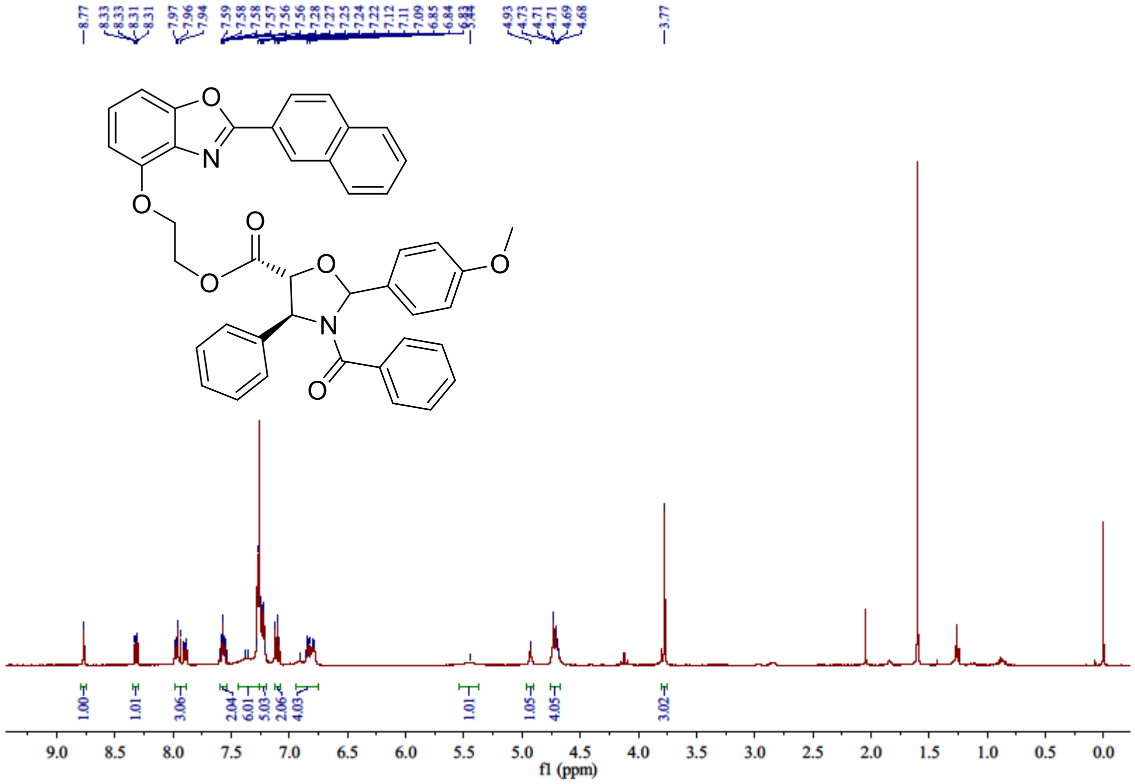

Figure S32 ^{1}H NMR (400 MHz) spectrum of compound **6f** in $CDCl_3$

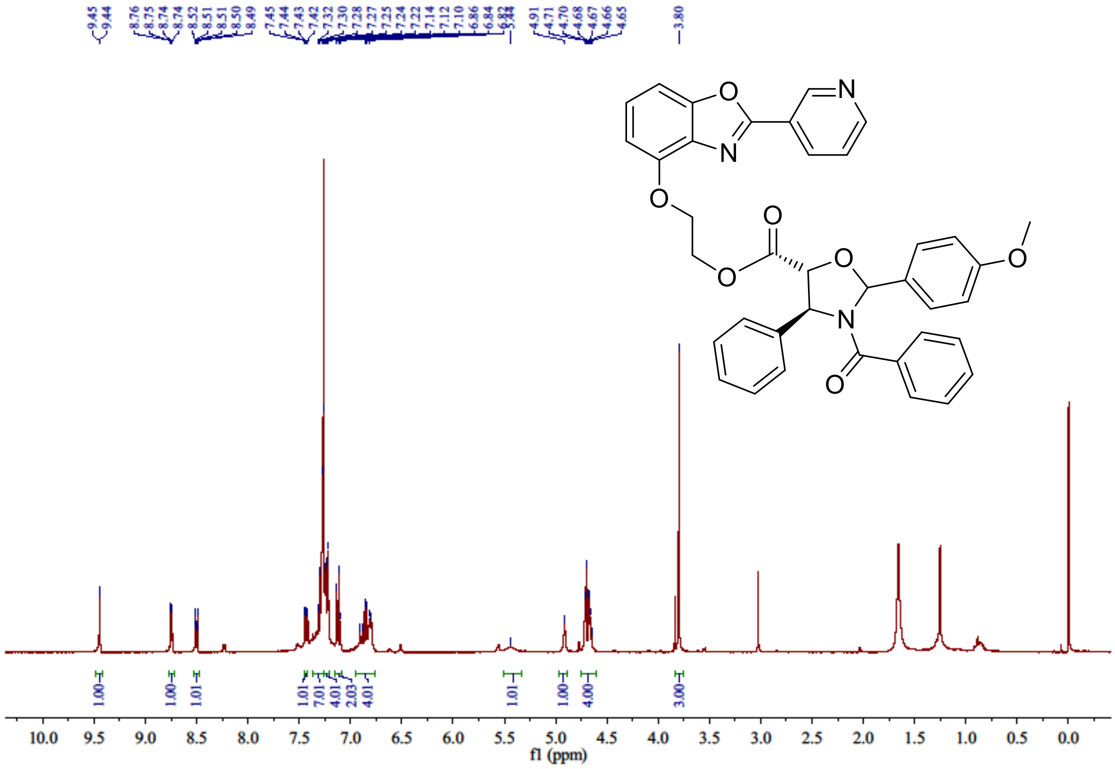

Figure S33 ^{1}H NMR (400 MHz) spectrum of compound **6g** in $CDCl_3$

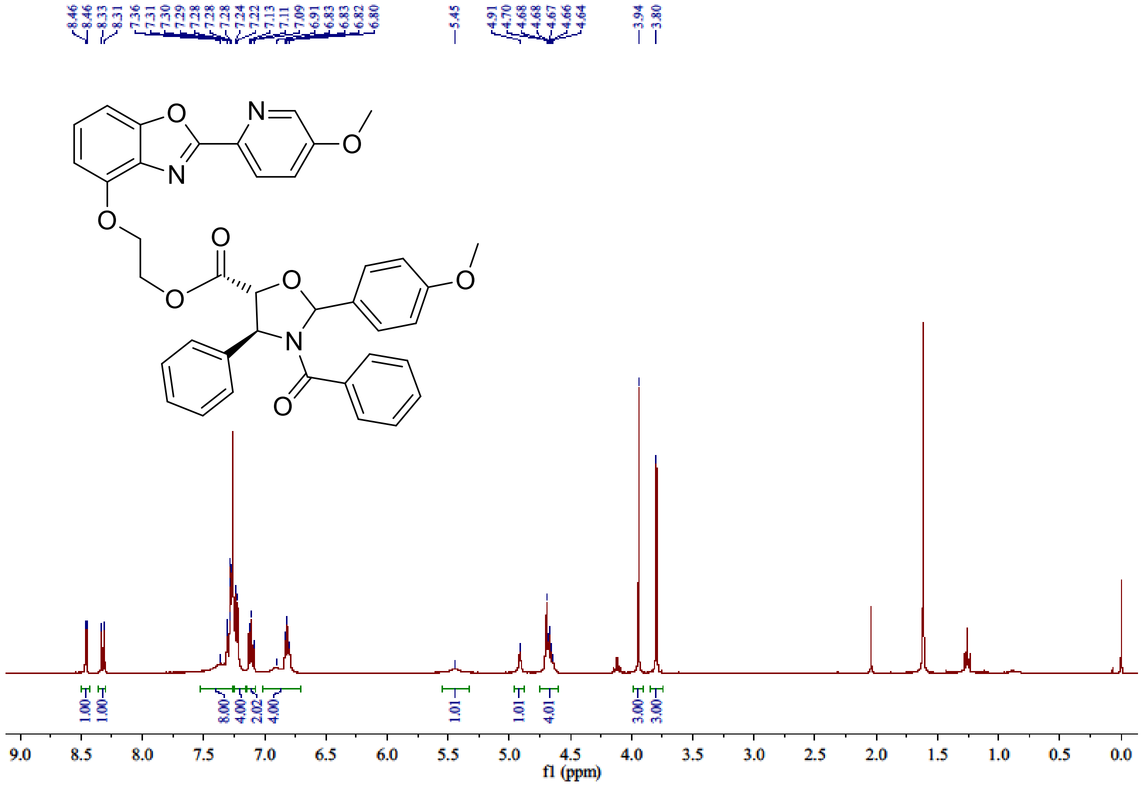

Figure S34 ^{1}H NMR (400 MHz) spectrum of compound **6h** in $CDCl_3$

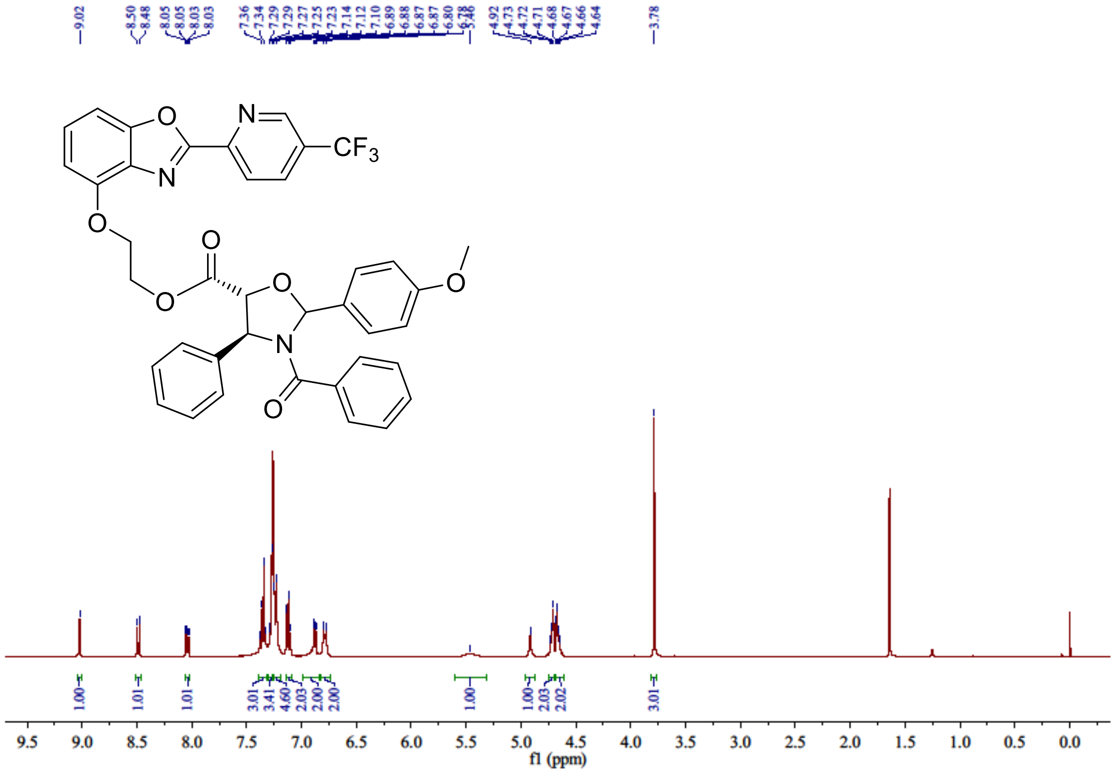

Figure S35 ^{1}H NMR (400 MHz) spectrum of compound **6i** in $CDCl_3$

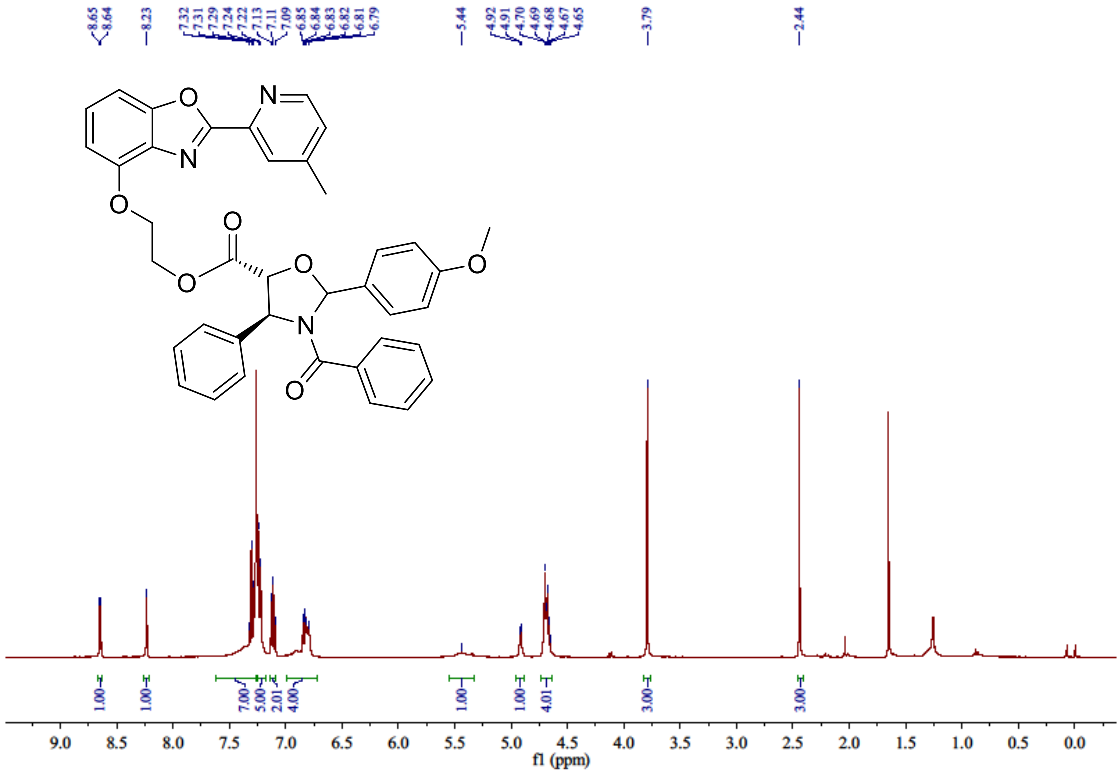

Figure S36 ^{1}H NMR (400 MHz) spectrum of compound **6j** in $CDCl_3$

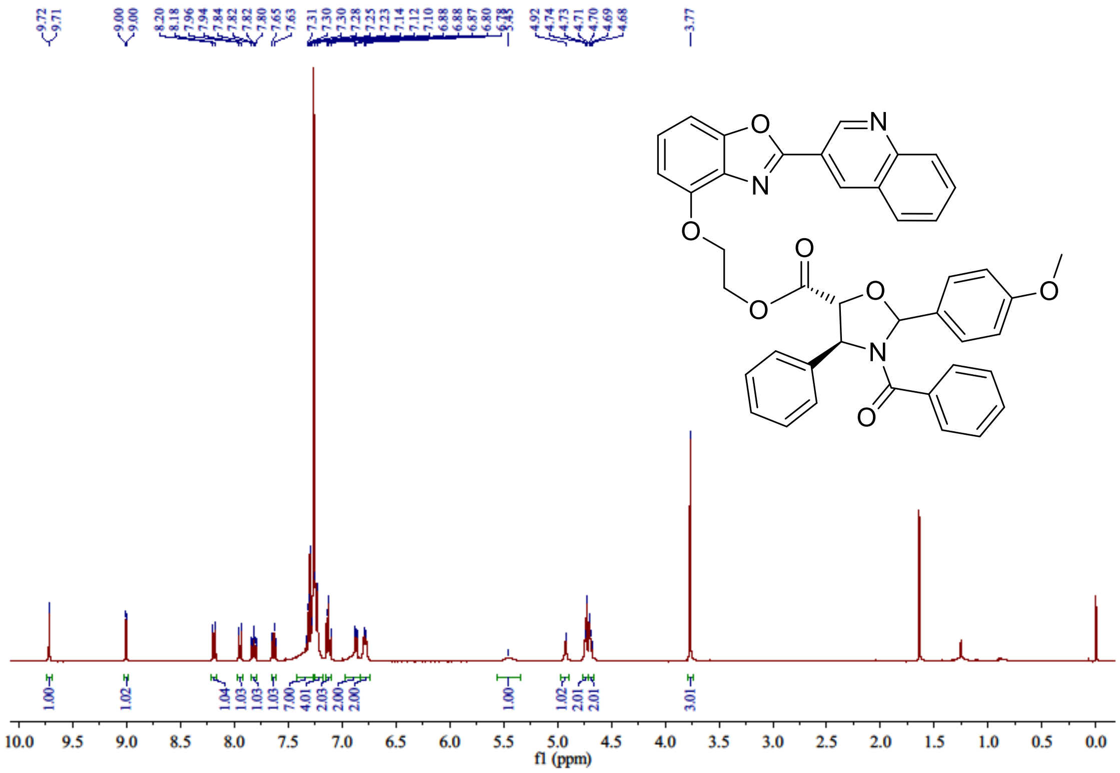

Figure S37 ^{1}H NMR (400 MHz) spectrum of compound **6k** in $CDCl_3$

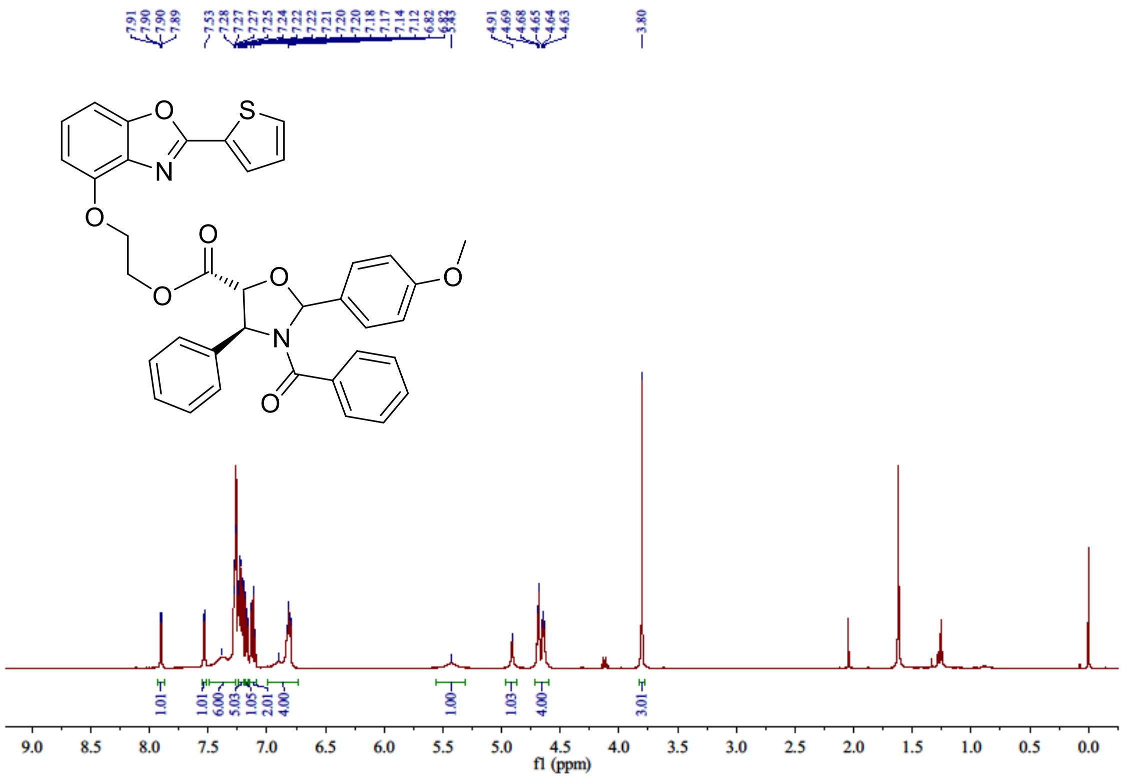

Figure S38 ^{1}H NMR (400 MHz) spectrum of compound **6l** in $CDCl_3$

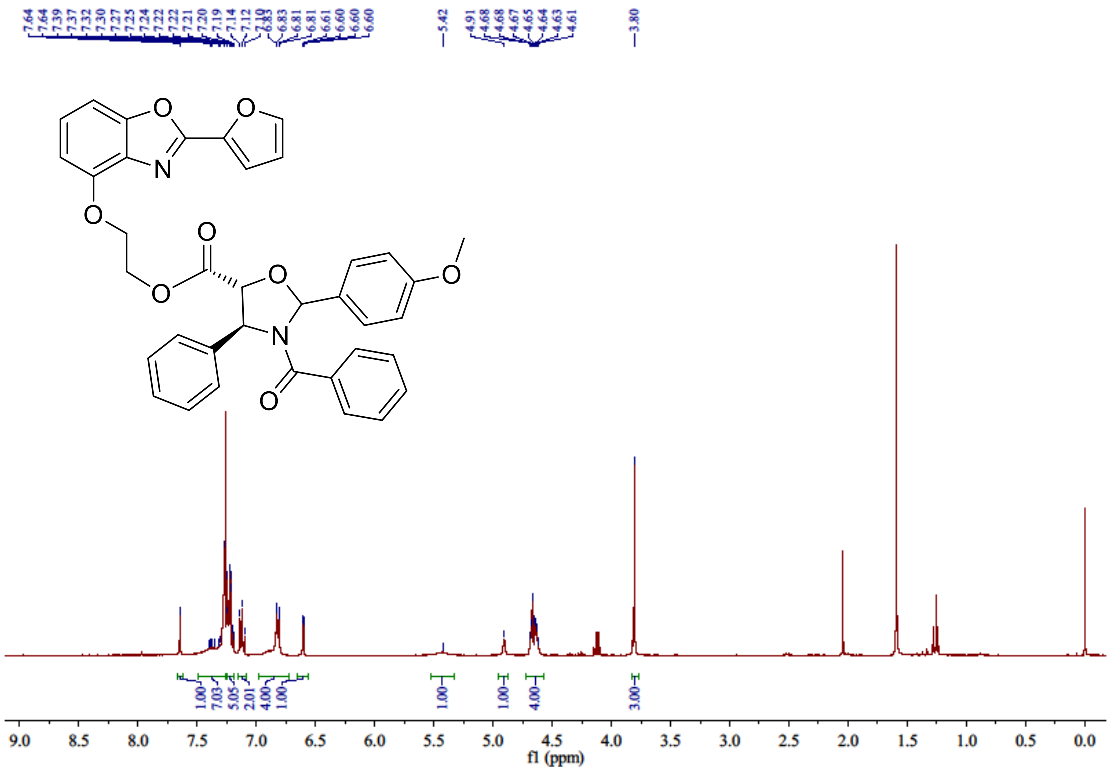

Figure S39 ^{1}H NMR (400 MHz) spectrum of compound **6m** in $CDCl_3$

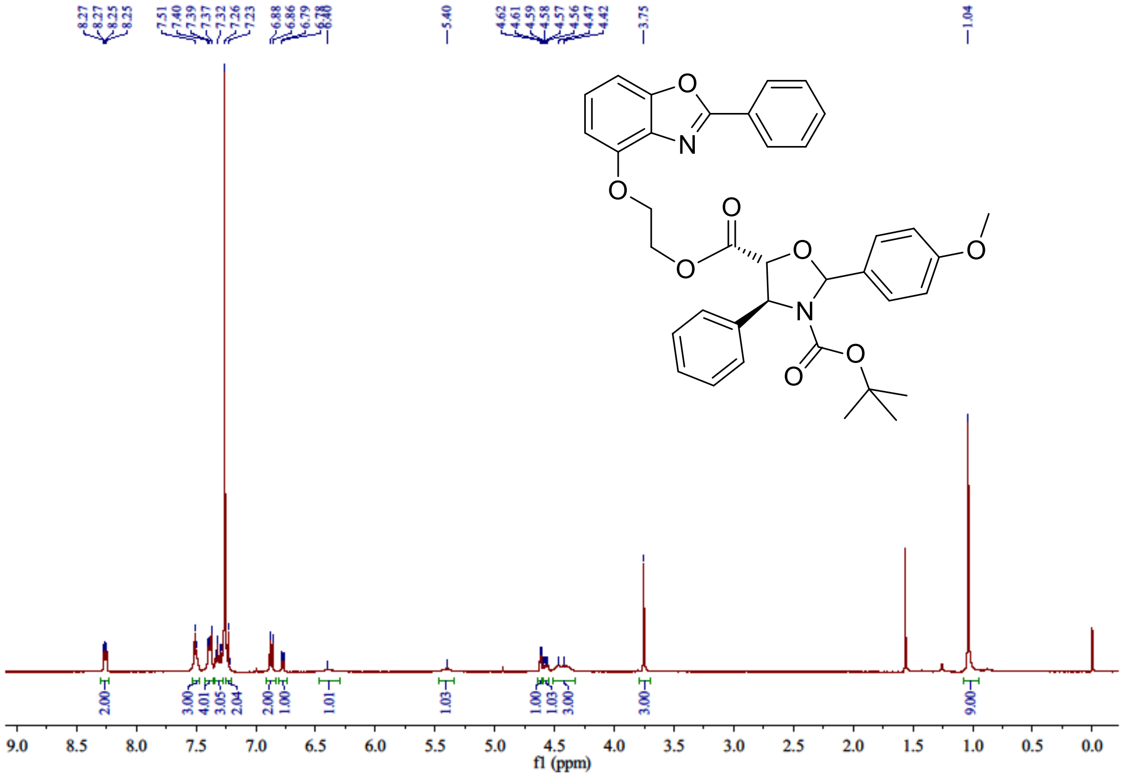

Figure S40 ^{1}H NMR (400 MHz) spectrum of compound **6A** in $CDCl_3$

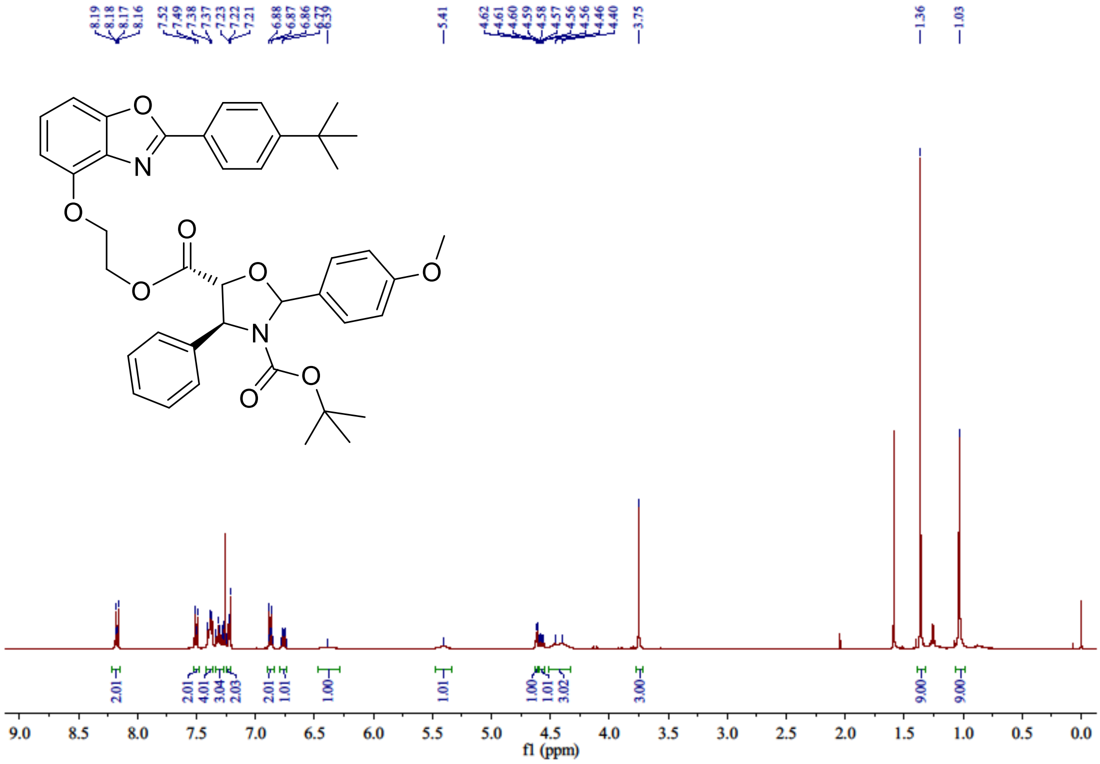

Figure S41 ^{1}H NMR (400 MHz) spectrum of compound **6B** in $CDCl_3$

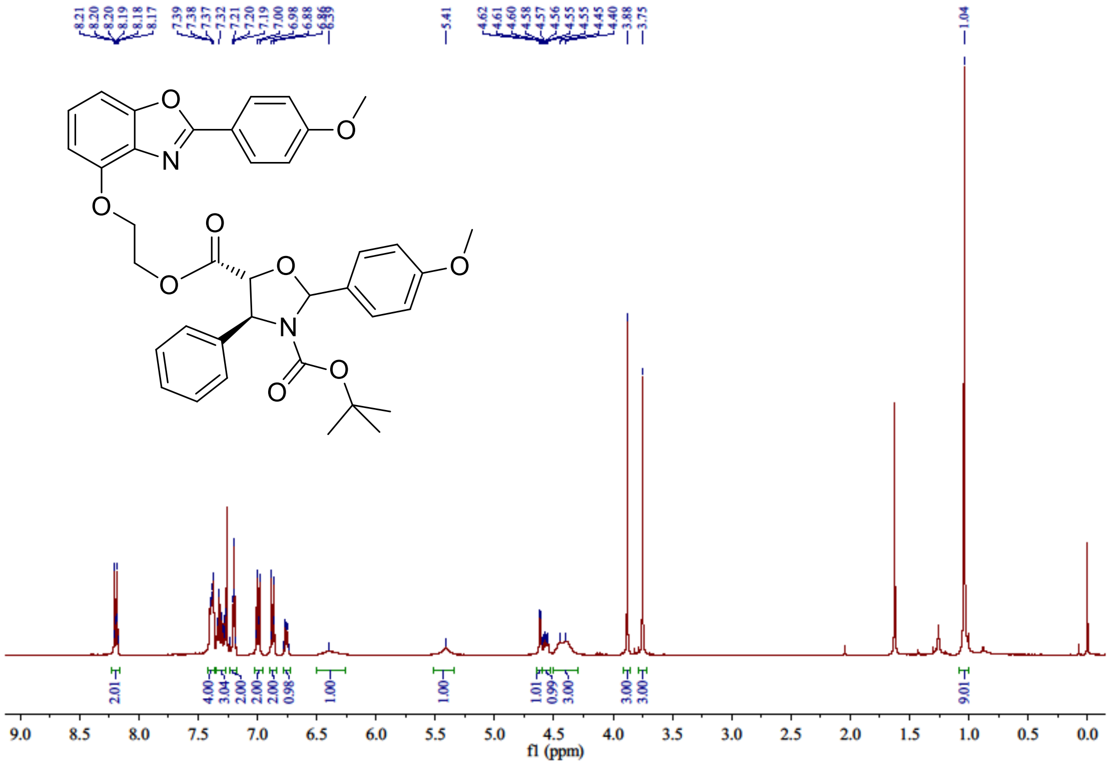

Figure S42 ^{1}H NMR (400 MHz) spectrum of compound **6C** in $CDCl_3$

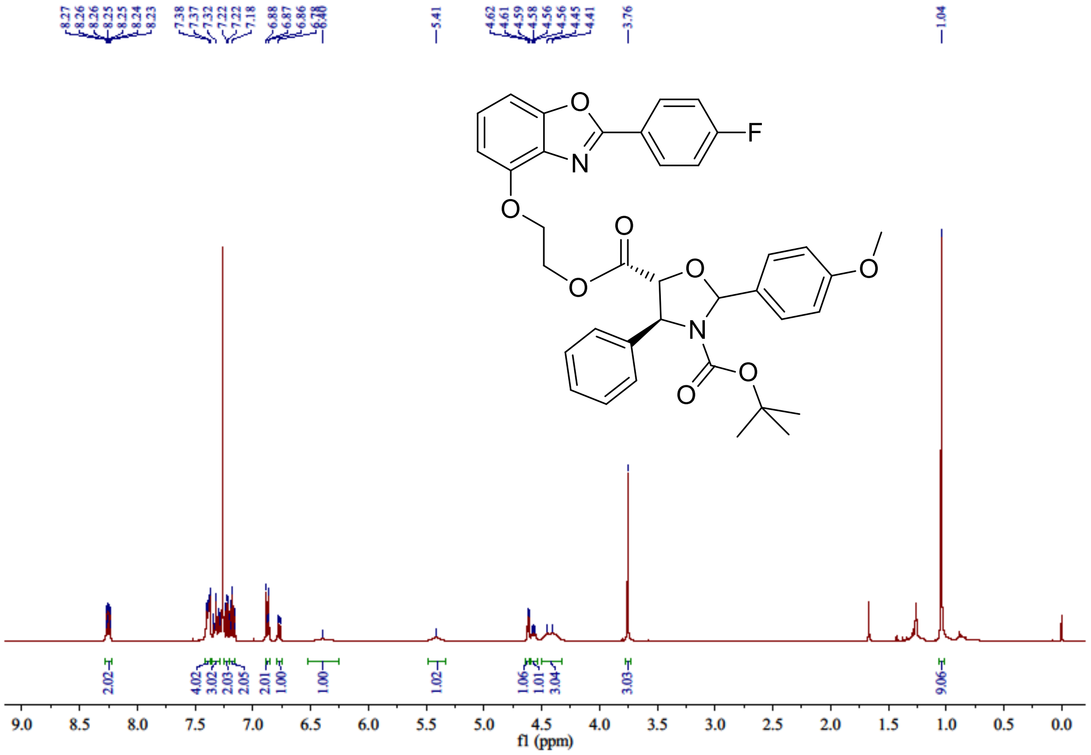

Figure S43 ^{1}H NMR (400 MHz) spectrum of compound **6D** in $CDCl_3$

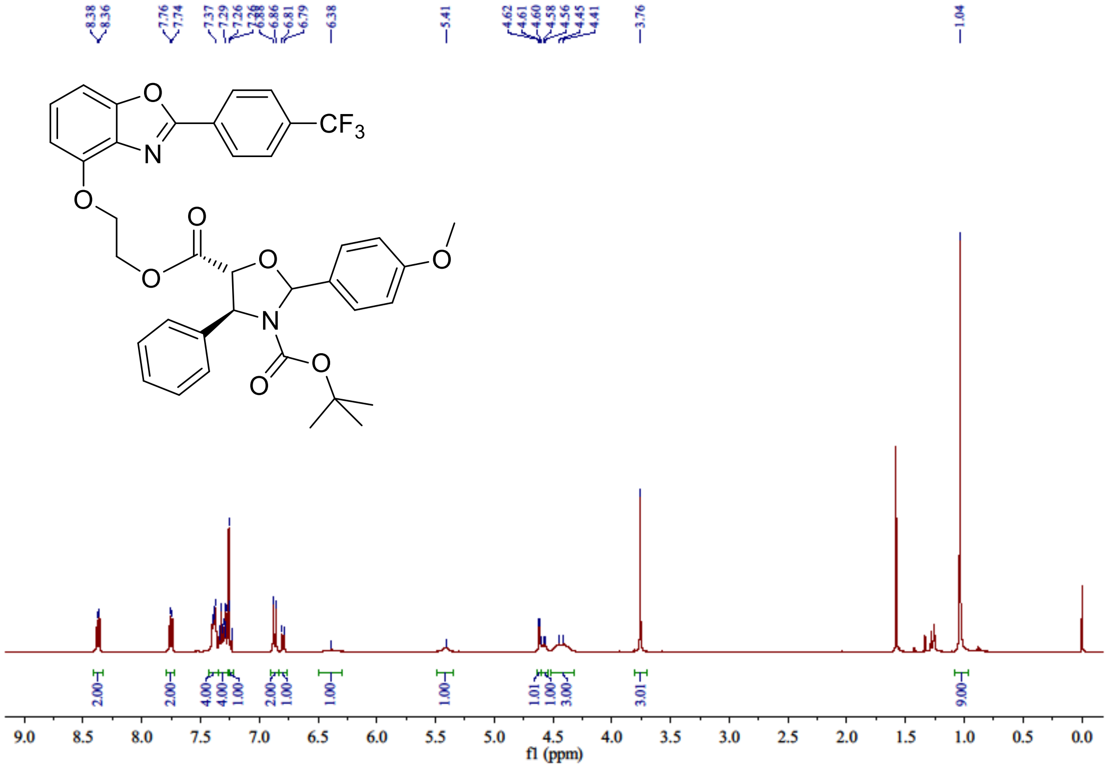

Figure S44 ^{1}H NMR (400 MHz) spectrum of compound **6E** in $CDCl_3$

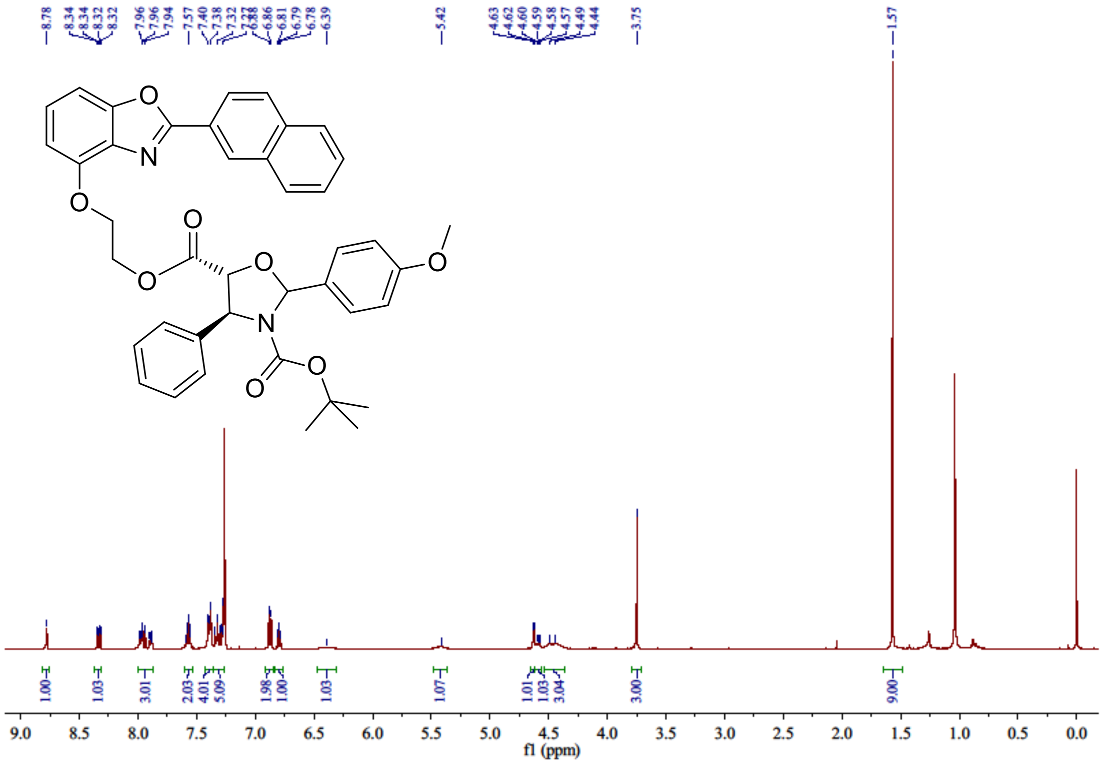

Figure S45 ^{1}H NMR (400 MHz) spectrum of compound **6F** in $CDCl_3$

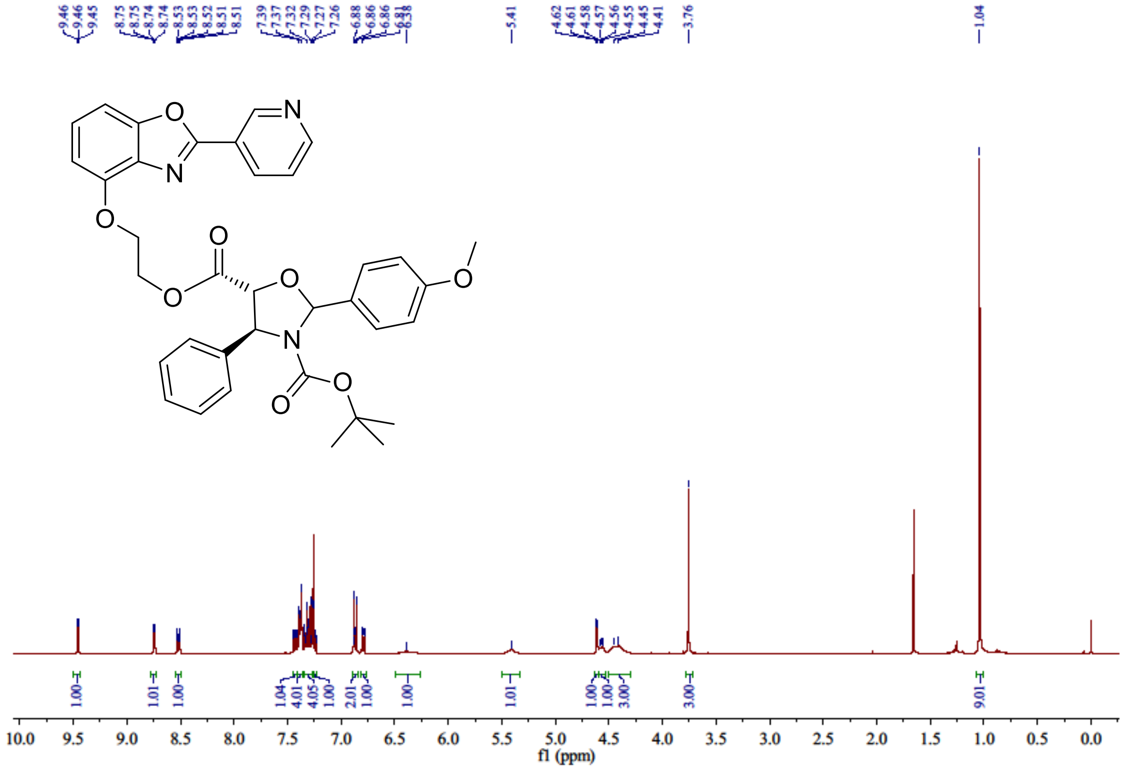

Figure S46 ^{1}H NMR (400 MHz) spectrum of compound **6G** in $CDCl_3$

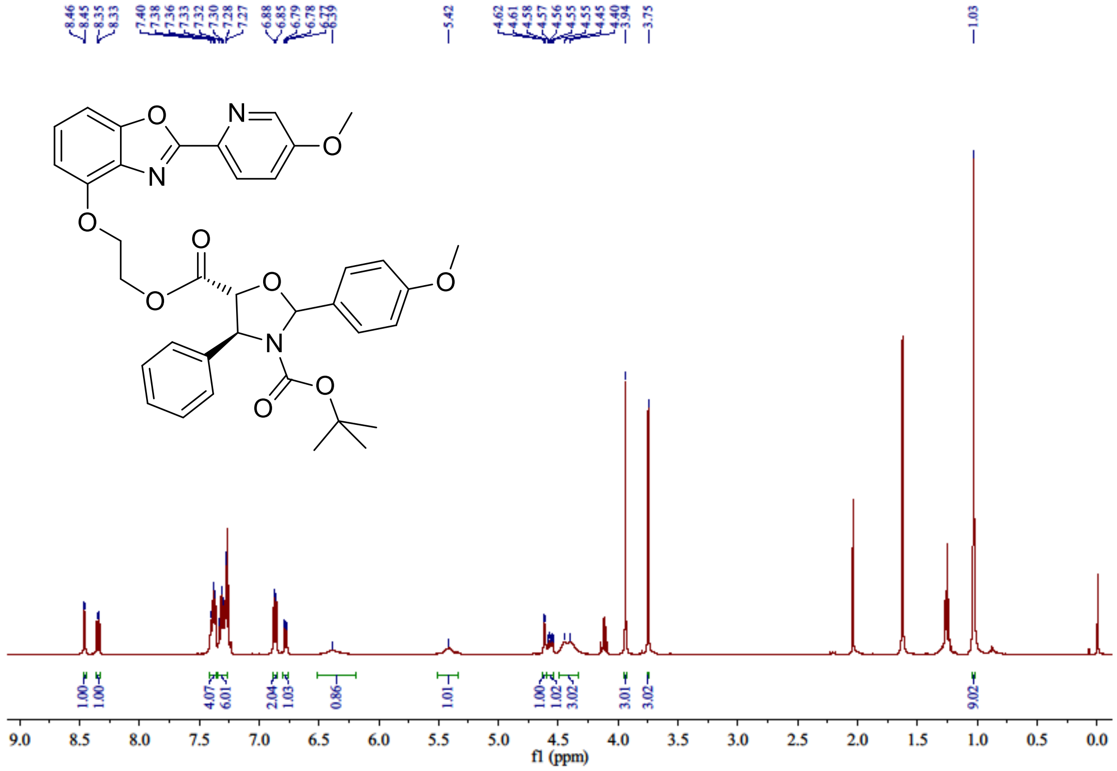

Figure S47 ^{1}H NMR (400 MHz) spectrum of compound **6H** in $CDCl_3$

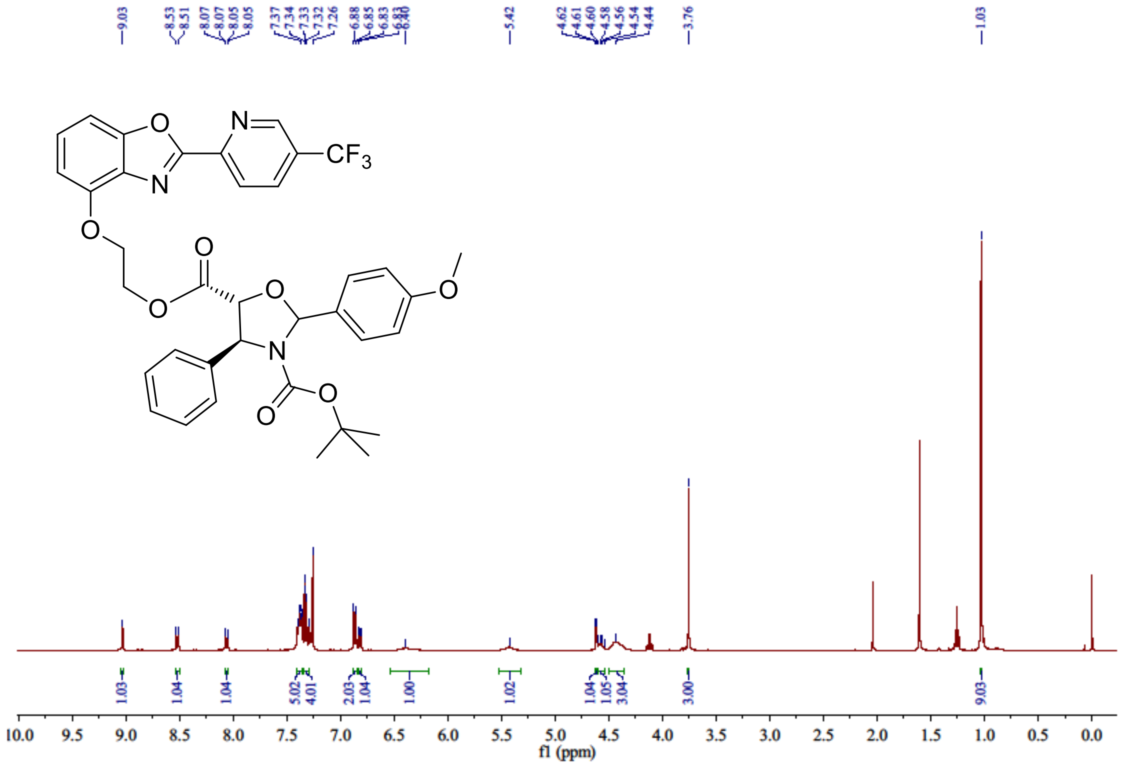

Figure S48 ^{1}H NMR (400 MHz) spectrum of compound **6I** in $CDCl_3$

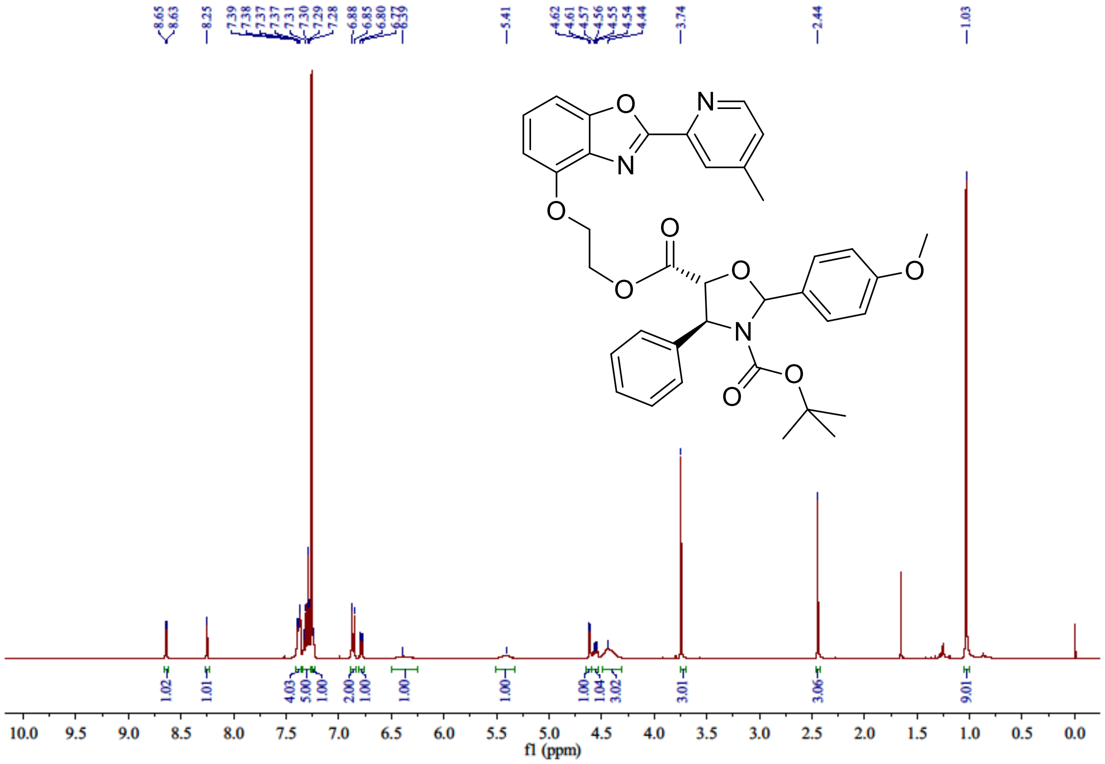

Figure S49 ^{1}H NMR (400 MHz) spectrum of compound **6J** in $CDCl_3$

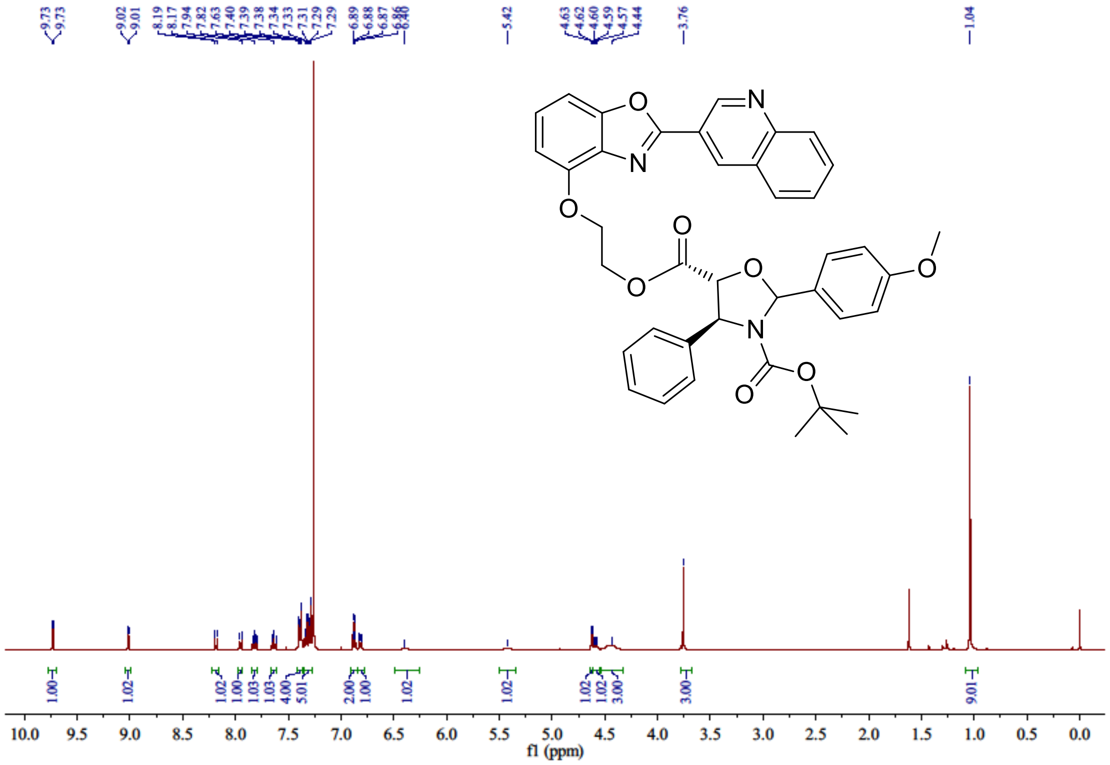

Figure S50 ^{1}H NMR (400 MHz) spectrum of compound **6K** in $CDCl_3$

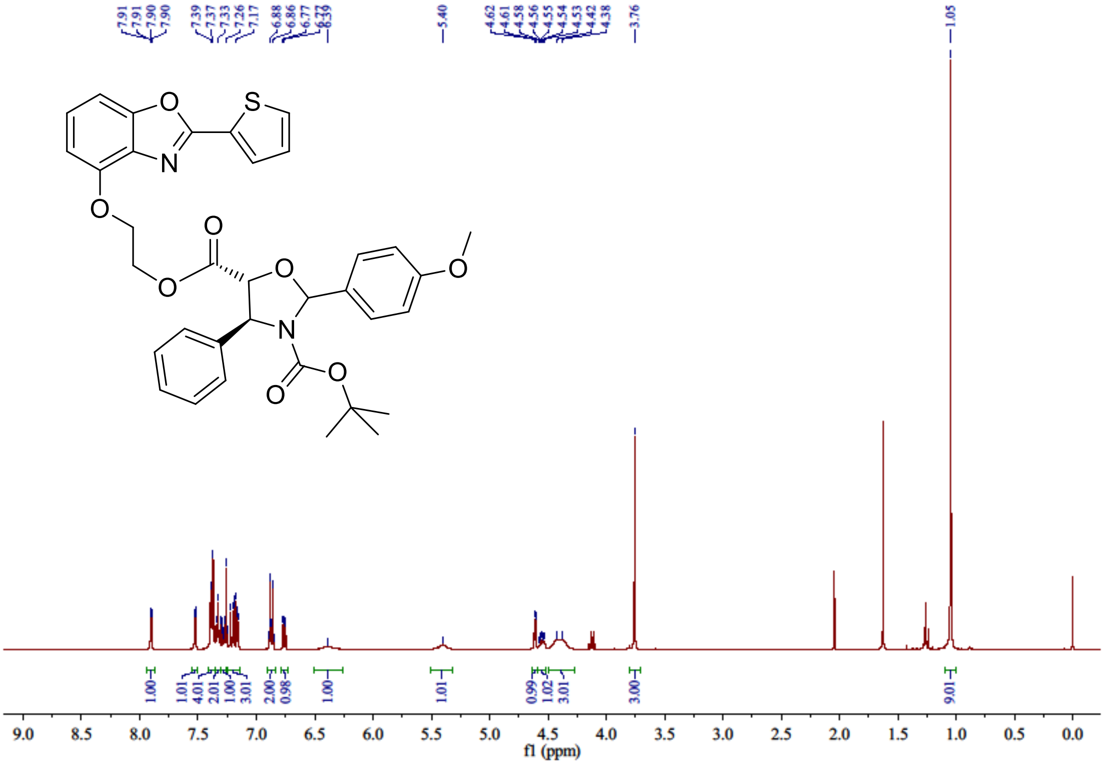

Figure S51 ^{1}H NMR (400 MHz) spectrum of compound **6L** in $CDCl_3$

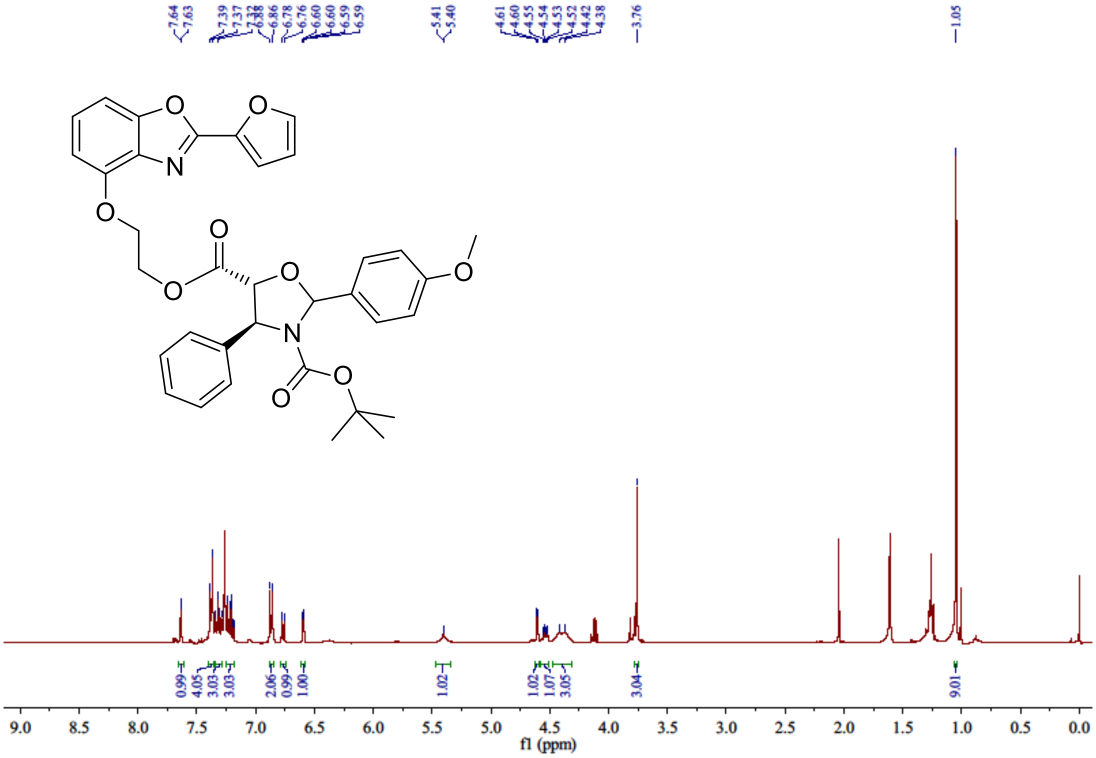

Figure S52 ^{1}H NMR (400 MHz) spectrum of compound **6M** in $CDCl_3$

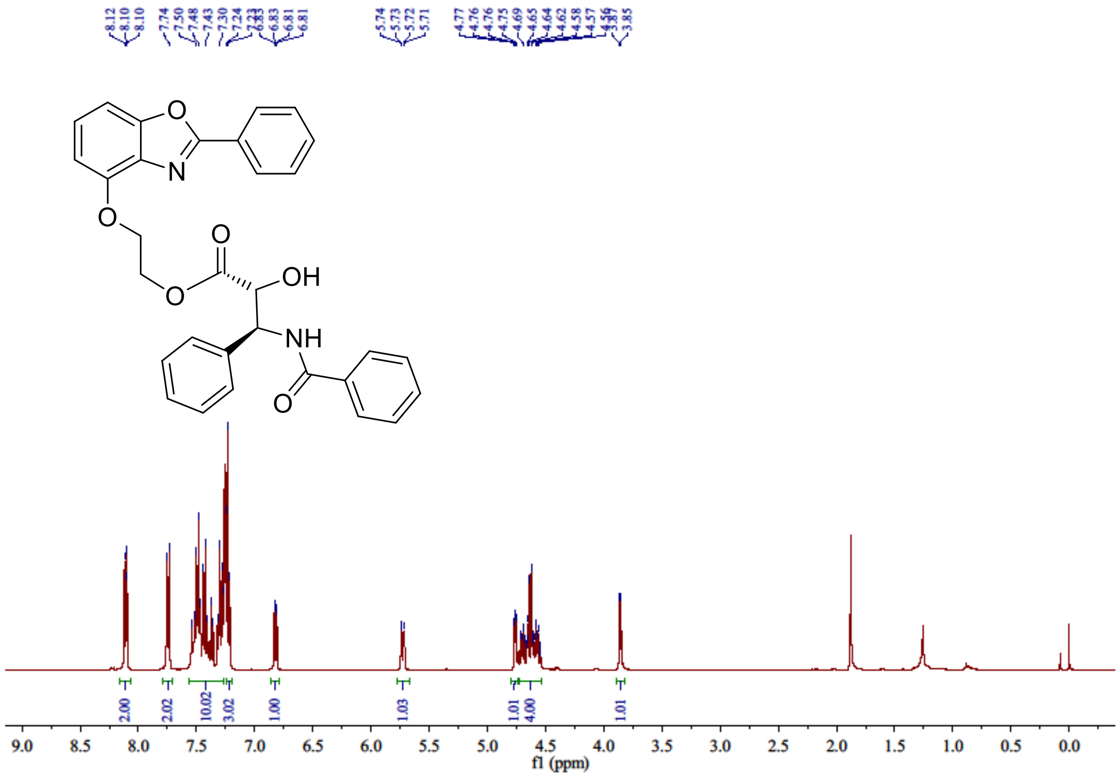

Figure S53 ^{1}H NMR (400 MHz) spectrum of compound **7a** in $CDCl_3$

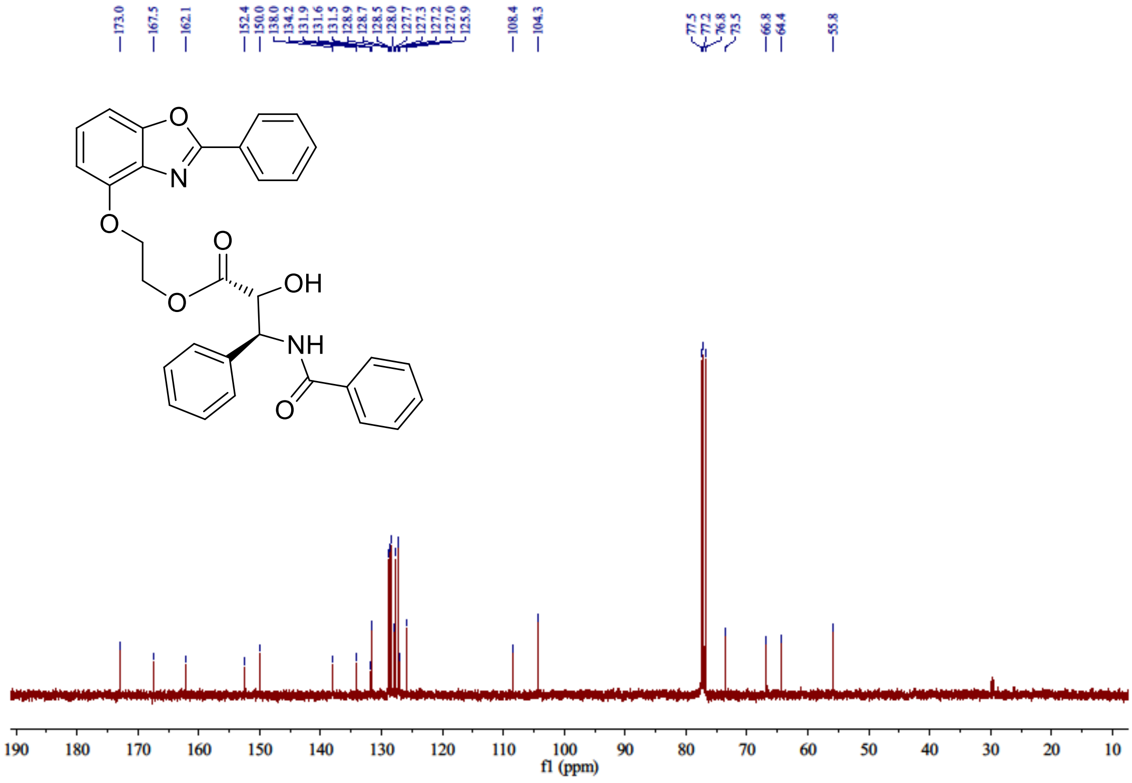

Figure S54 ^{13}C NMR (100 MHz) spectrum of compound **7a** in $CDCl_3$

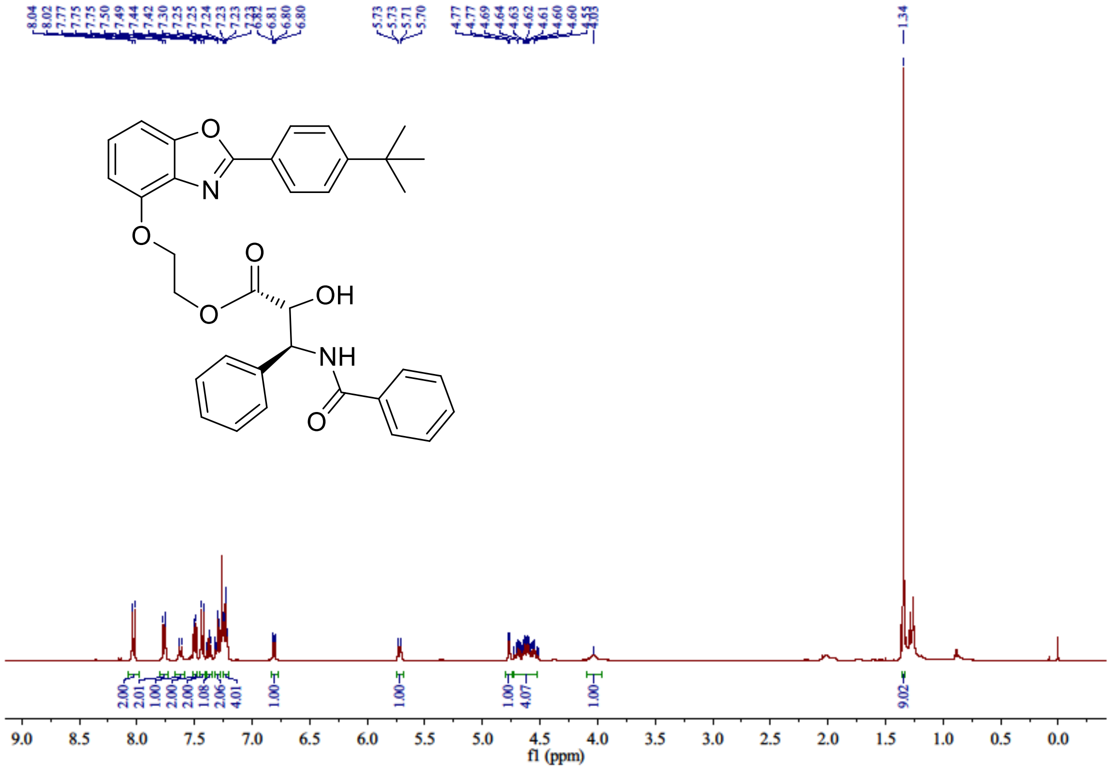

Figure S55 ^{1}H NMR (400 MHz) spectrum of compound **7b** in $CDCl_3$

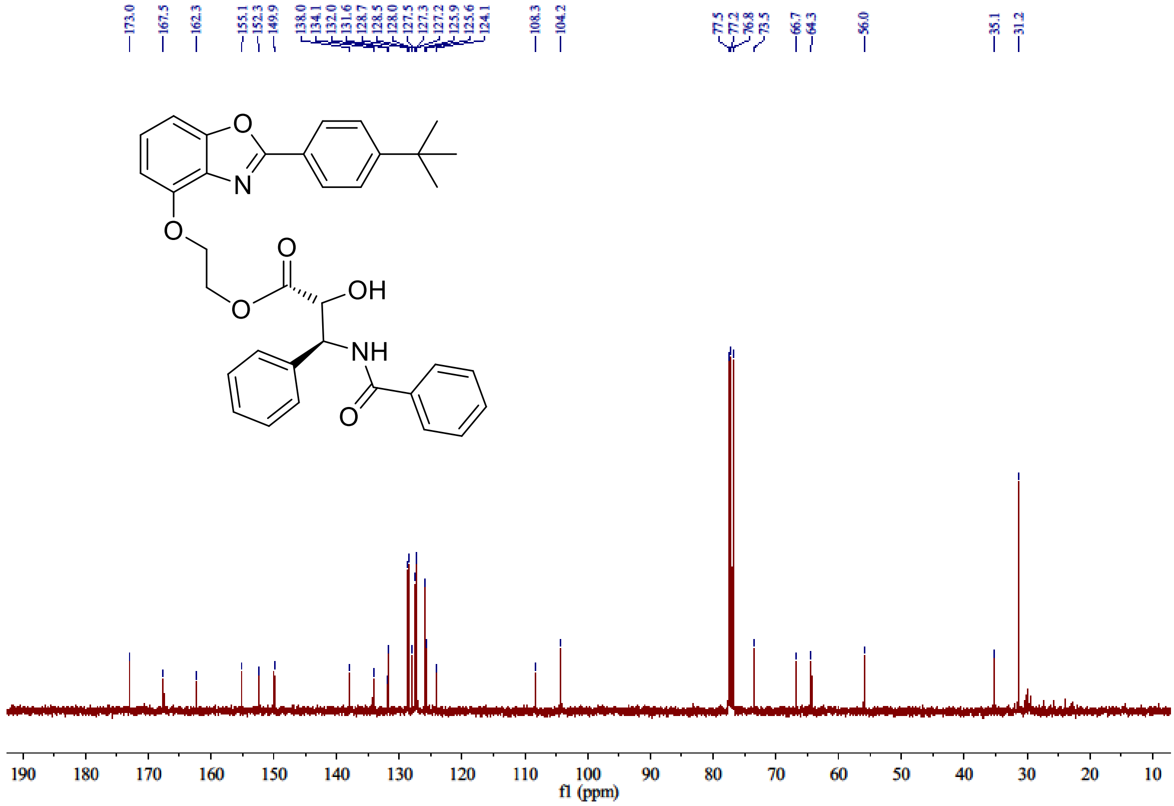

Figure S56 ^{13}C NMR (100 MHz) spectrum of compound **7b** in $CDCl_3$

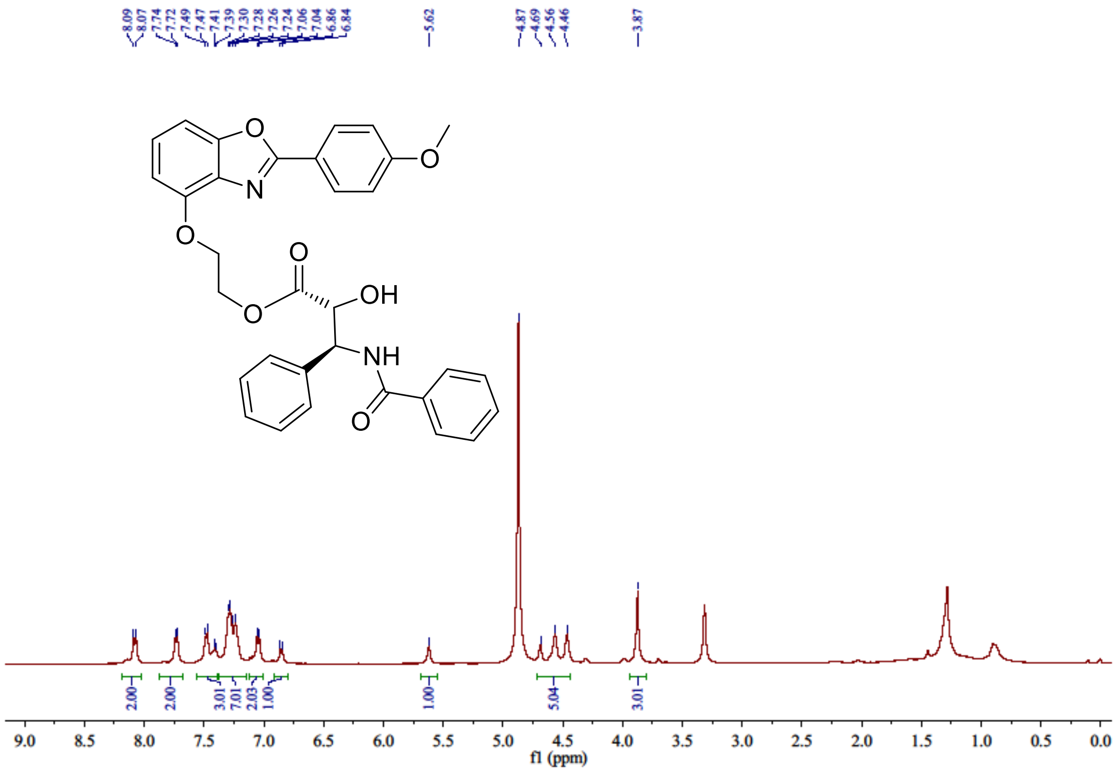

Figure S57 ^{1}H NMR (400 MHz) spectrum of compound **7c** in CD_3OD

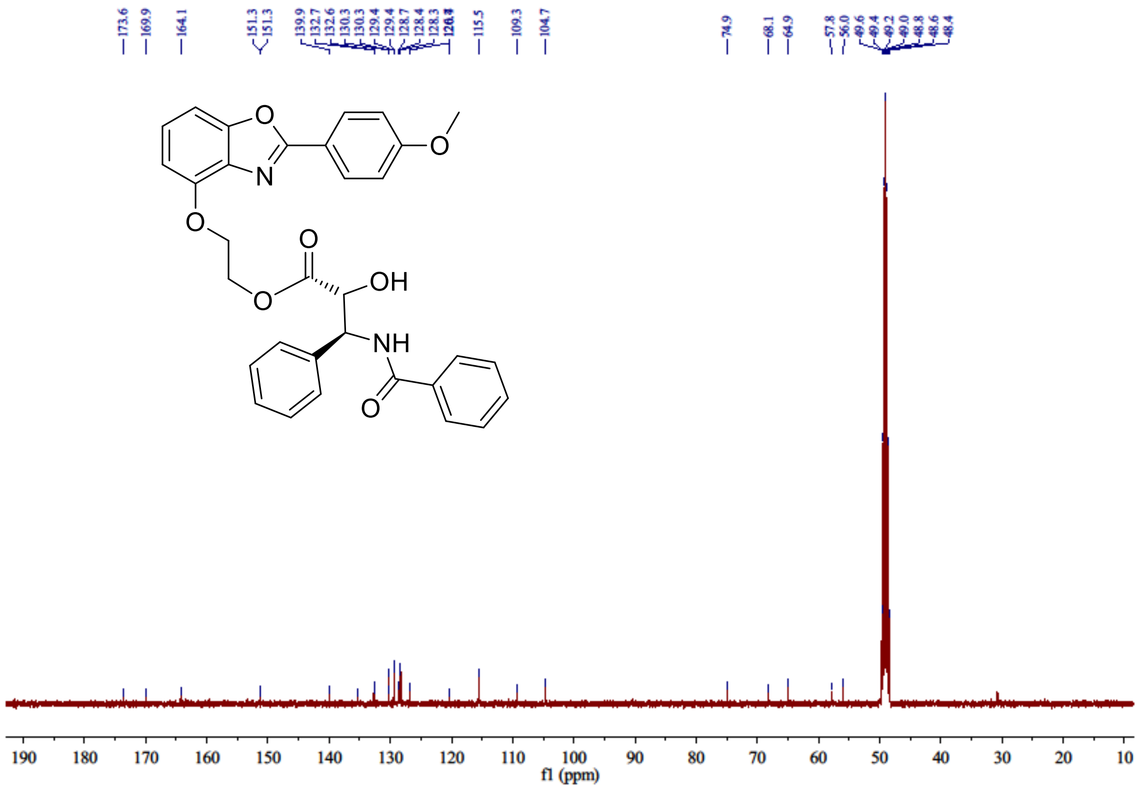

Figure S58 ^{13}C NMR (100 MHz) spectrum of compound **7c** in CD_3OD

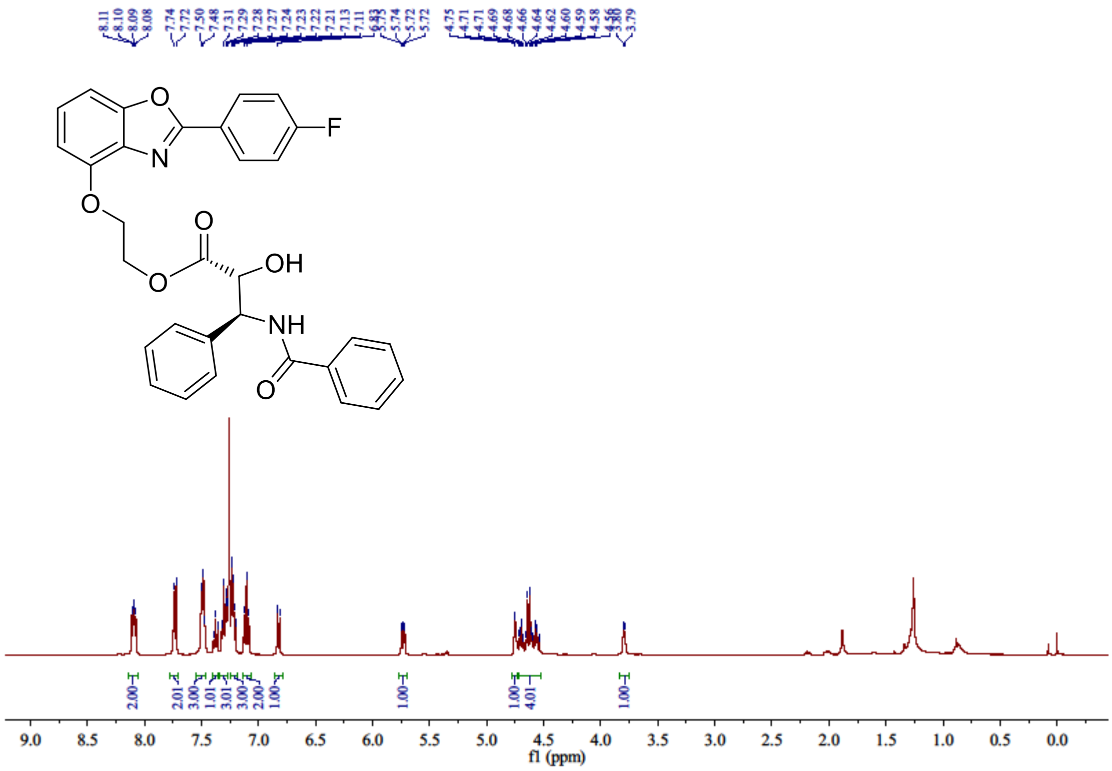

Figure S59 ^{1}H NMR (400 MHz) spectrum of compound **7d** in $CDCl_3$

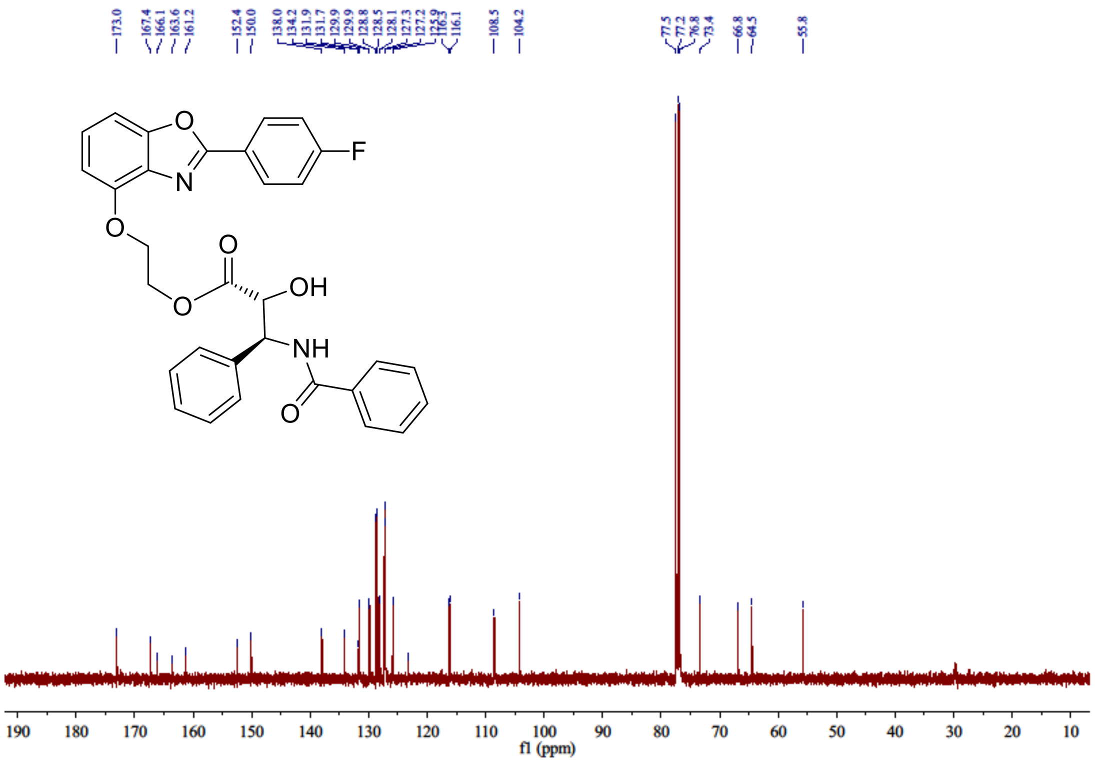

Figure S60 ^{13}C NMR (100 MHz) spectrum of compound **7d** in $CDCl_3$

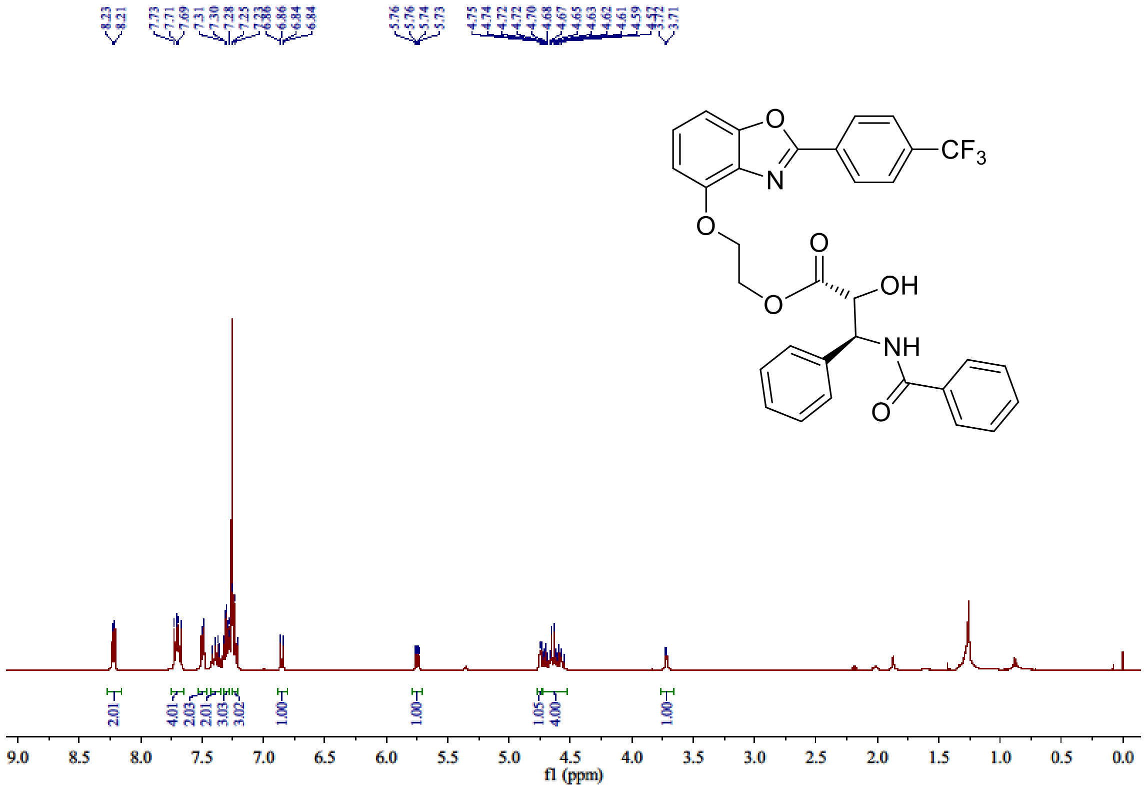

Figure S61 ^{1}H NMR (400 MHz) spectrum of compound **7e** in $CDCl_3$

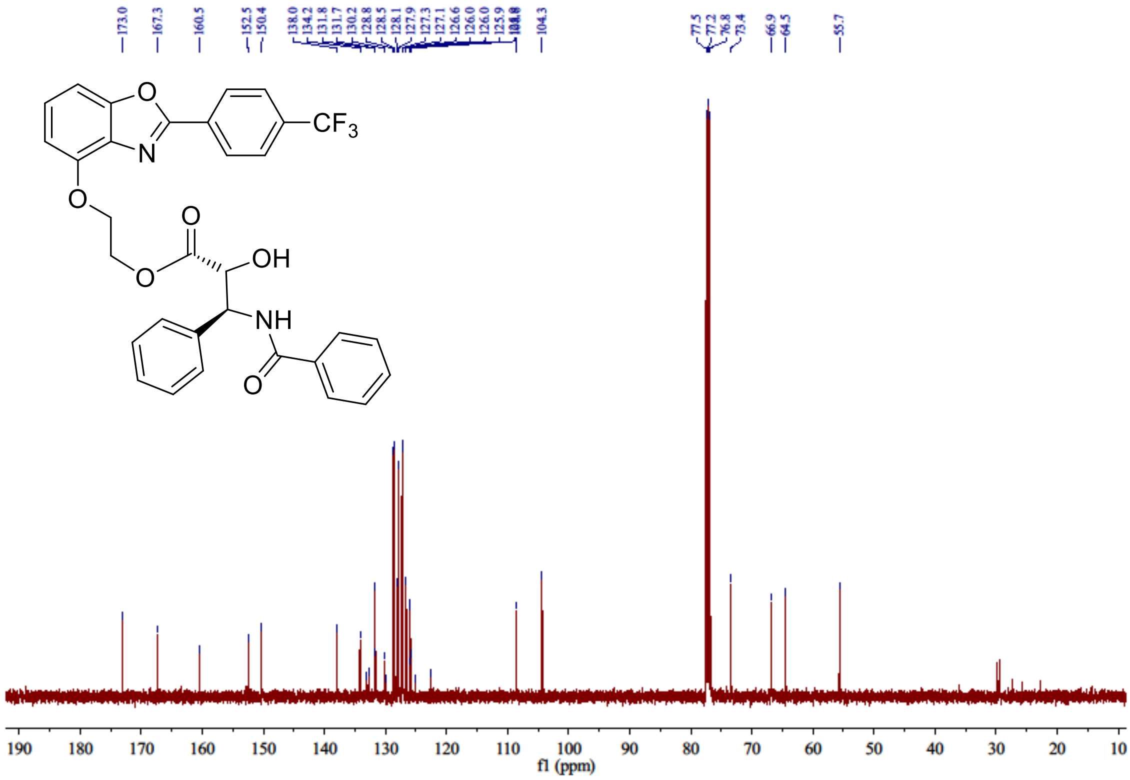

Figure S62 ^{13}C NMR (100 MHz) spectrum of compound **7e** in $CDCl_3$

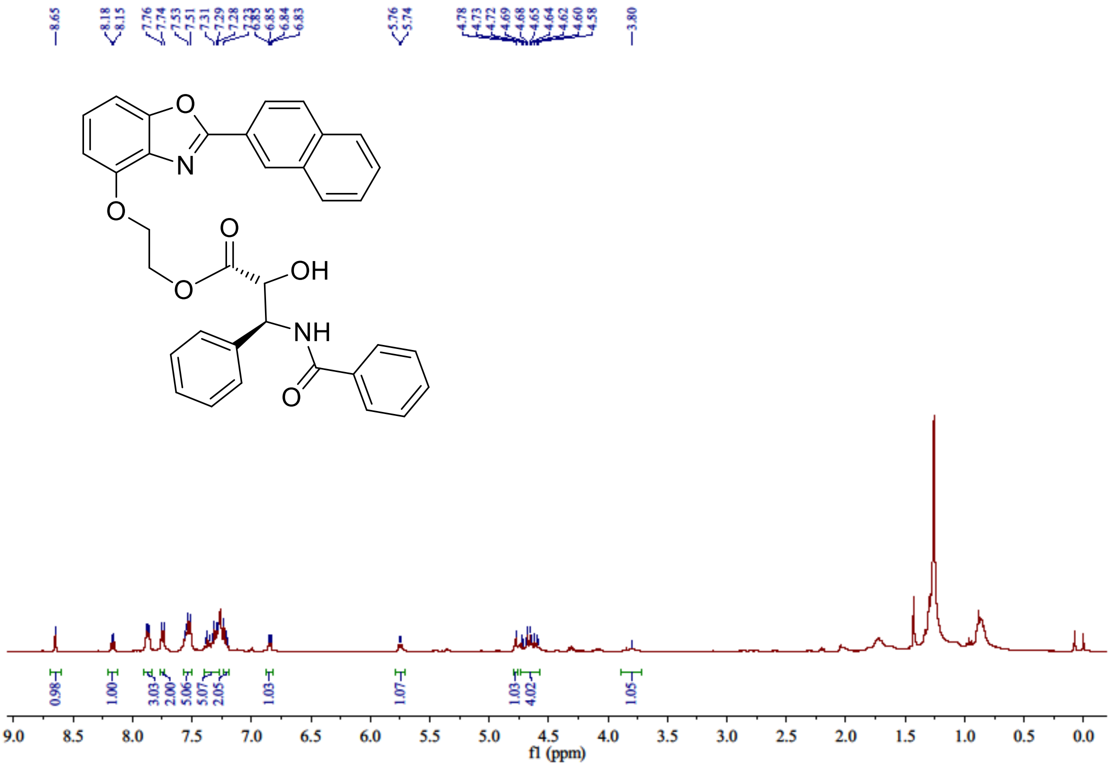

Figure S63 ^{1}H NMR (400 MHz) spectrum of compound **7f** in $CDCl_3$

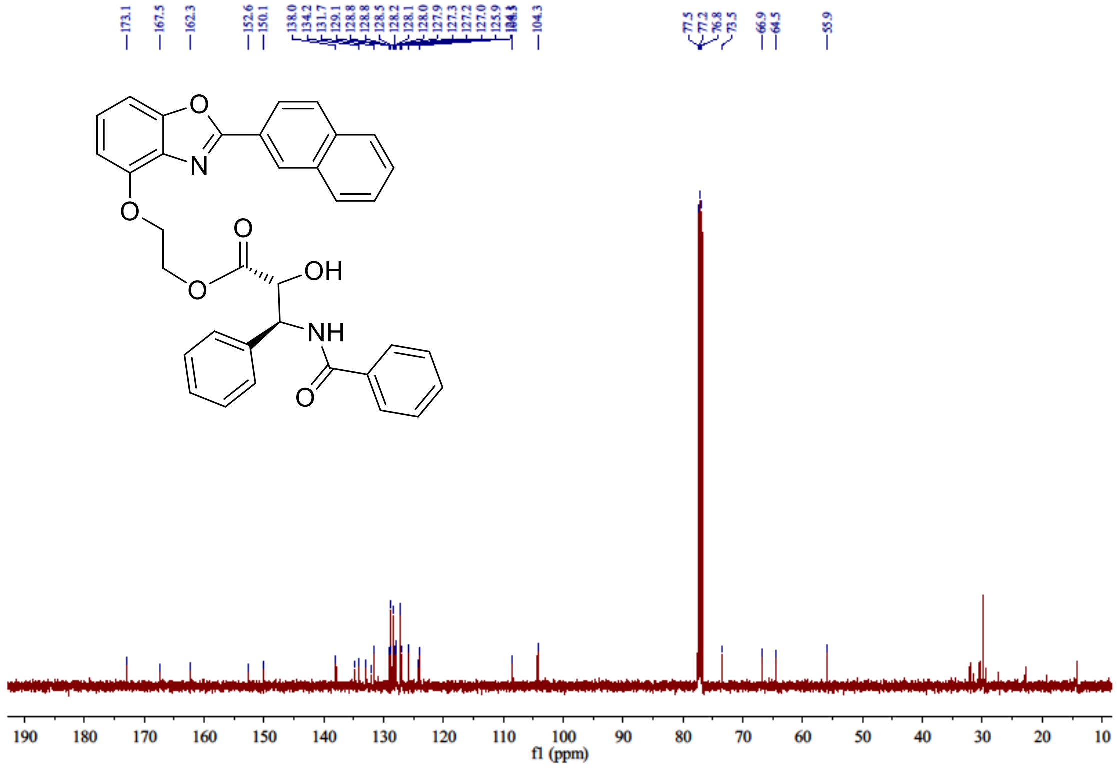

Figure S64 ^{13}C NMR (100 MHz) spectrum of compound **7f** in $CDCl_3$

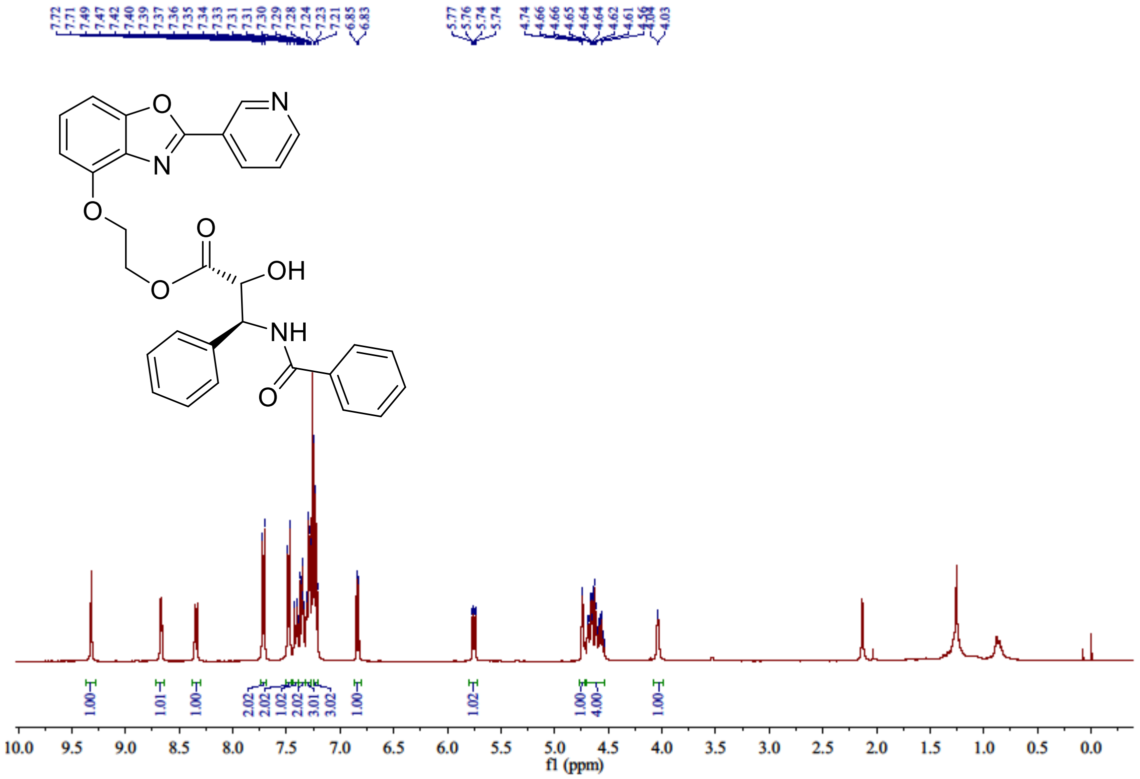

Figure S65 ^{1}H NMR (400 MHz) spectrum of compound **7g** in $CDCl_3$

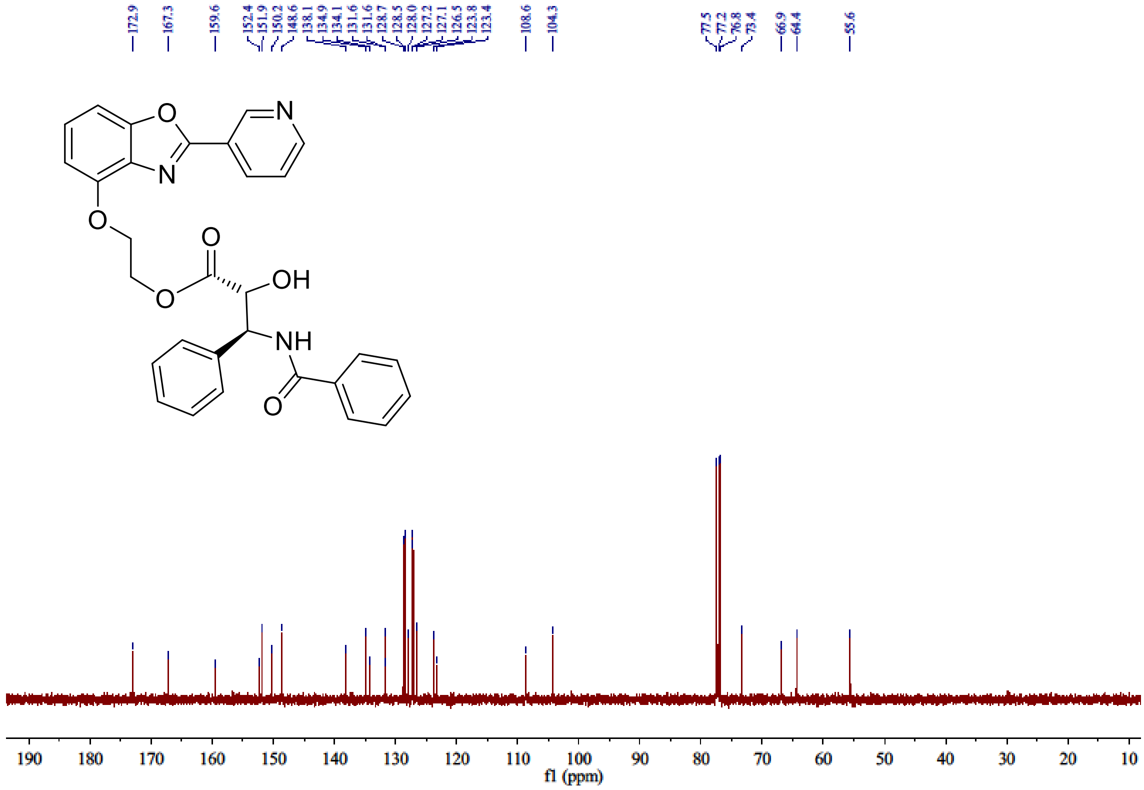

Figure S66 ^{13}C NMR (100 MHz) spectrum of compound **7g** in $CDCl_3$

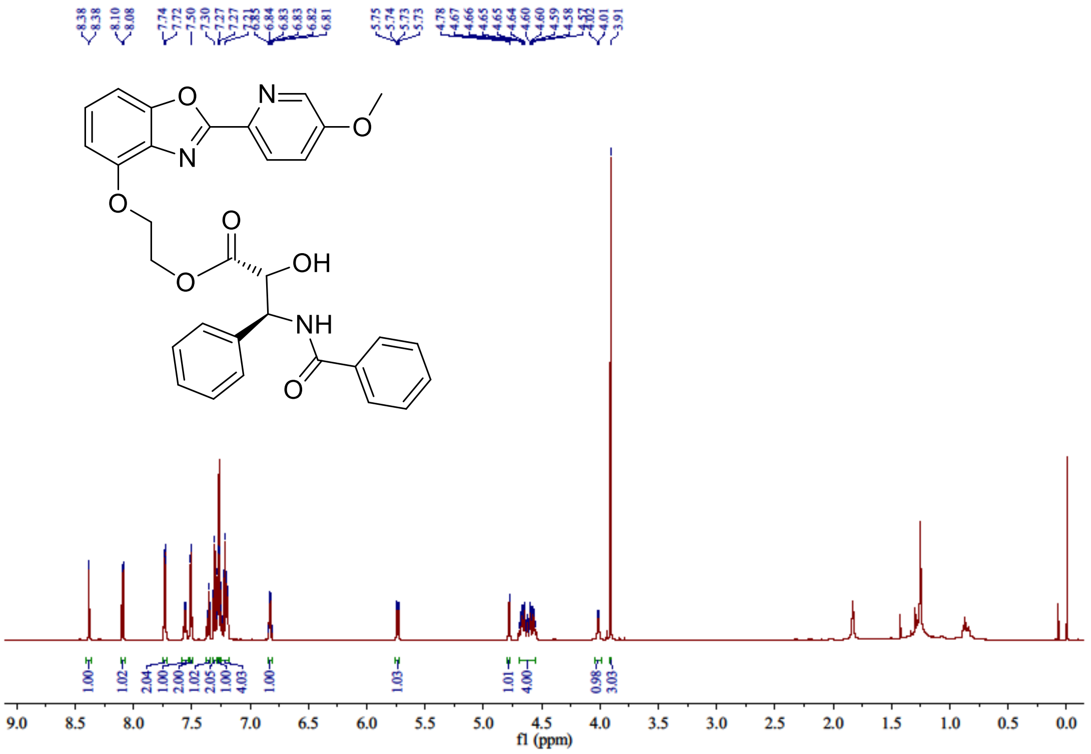

Figure S67 ^{1}H NMR (600 MHz) spectrum of compound **7h** in $CDCl_3$

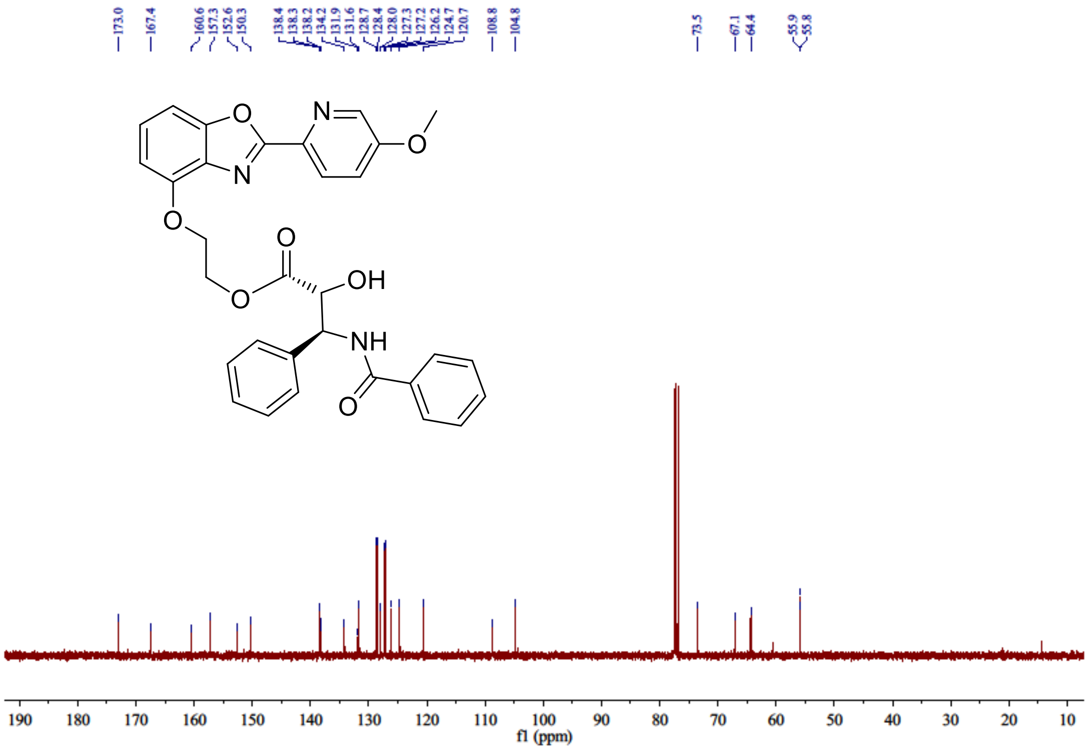

Figure S68 ^{13}C NMR (100 MHz) spectrum of compound **7h** in $CDCl_3$

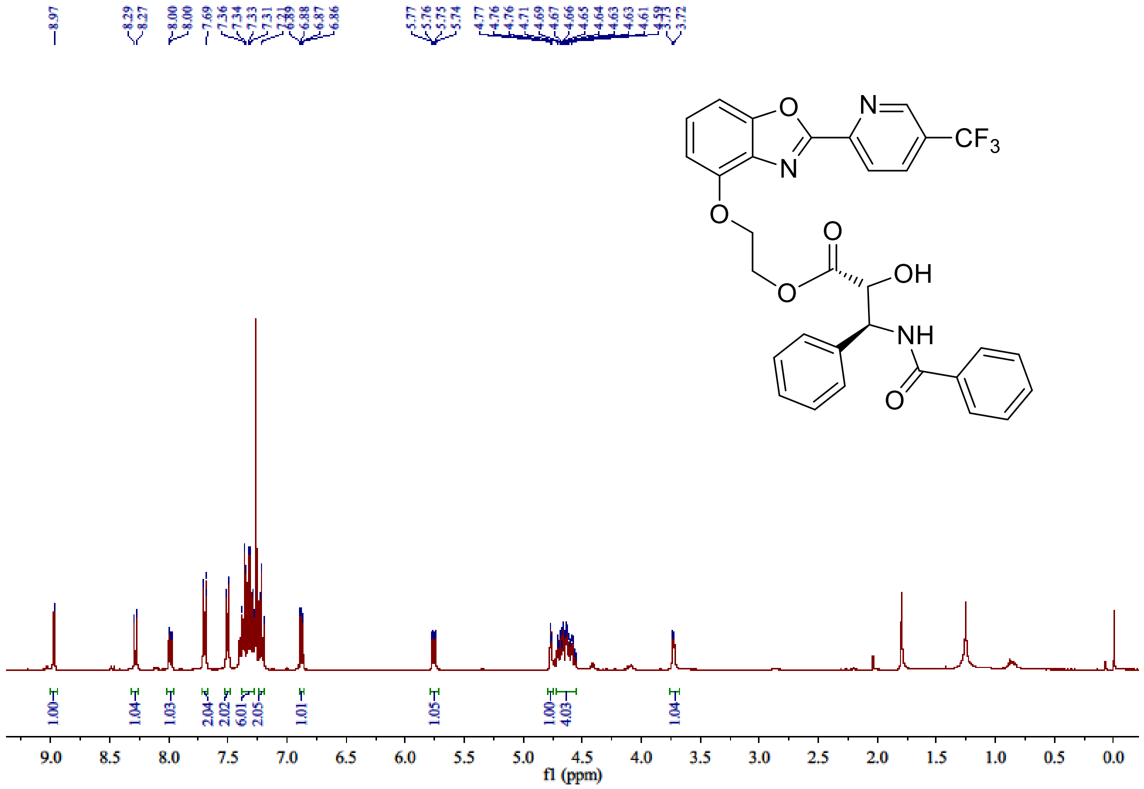

Figure S69 ^{1}H NMR (400 MHz) spectrum of compound **7i** in $CDCl_3$

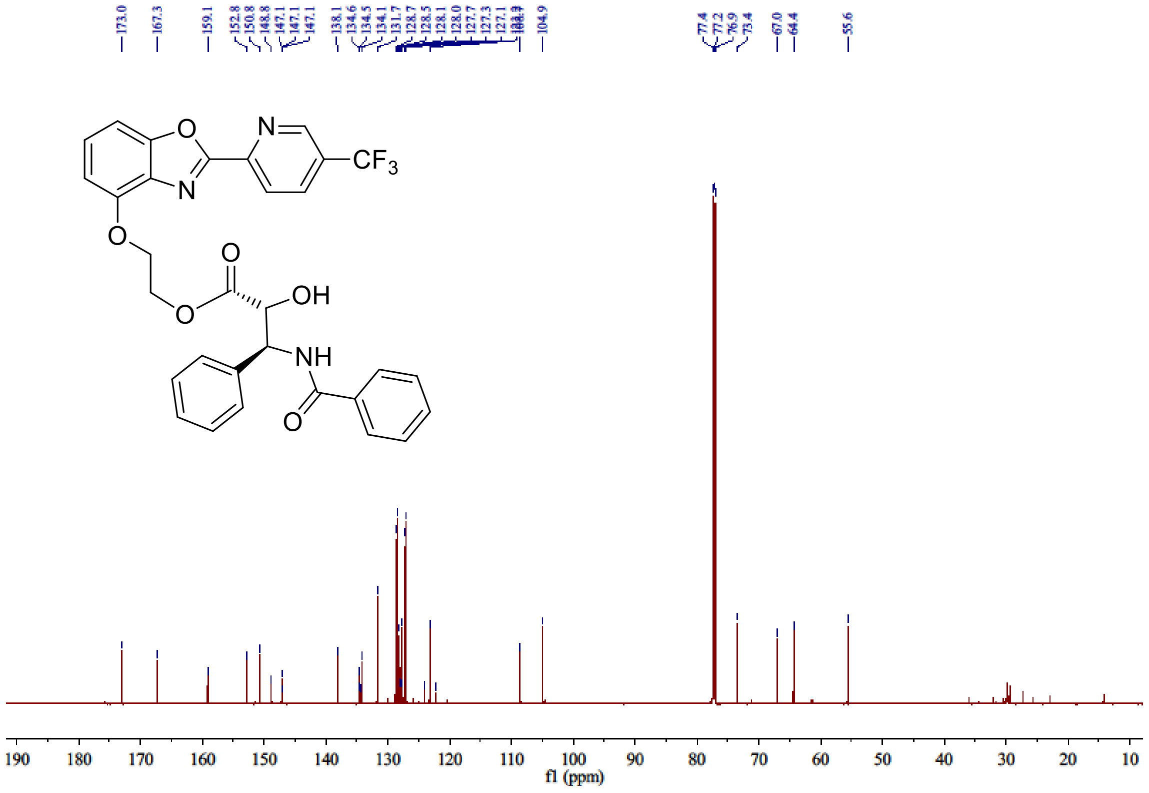

Figure S70 ^{13}C NMR (150 MHz) spectrum of compound **7i** in $CDCl_3$

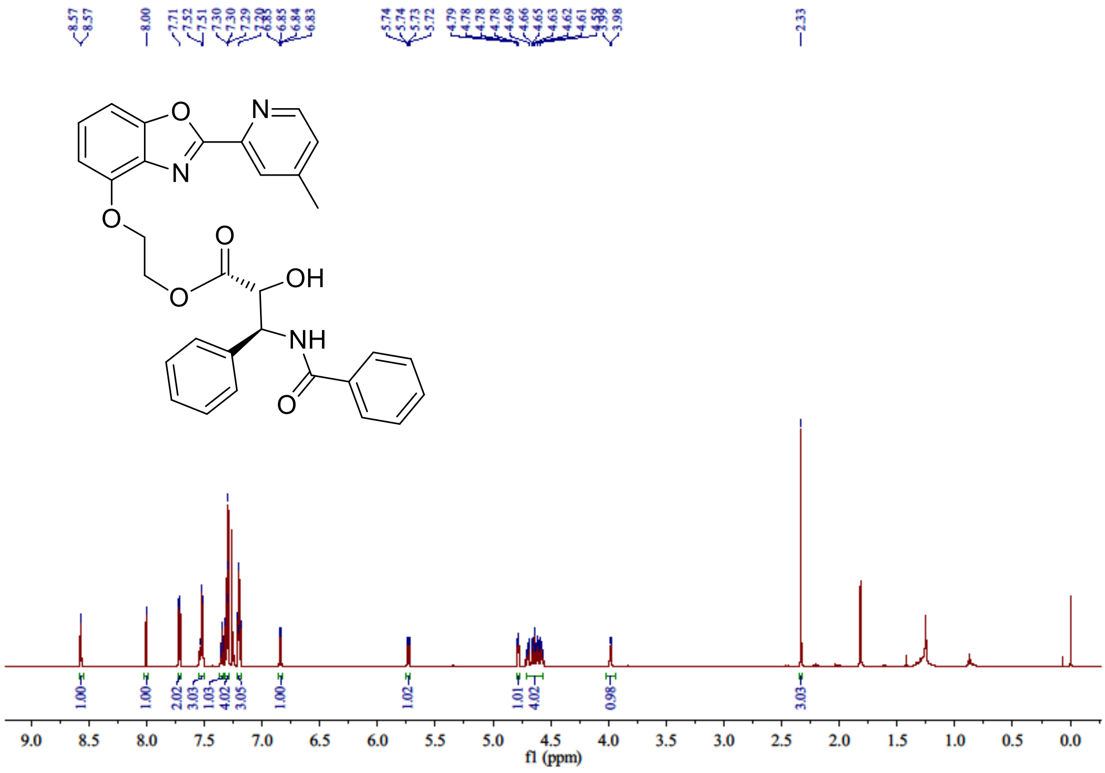

Figure S71 ^{1}H NMR (600 MHz) spectrum of compound **7j** in $CDCl_3$

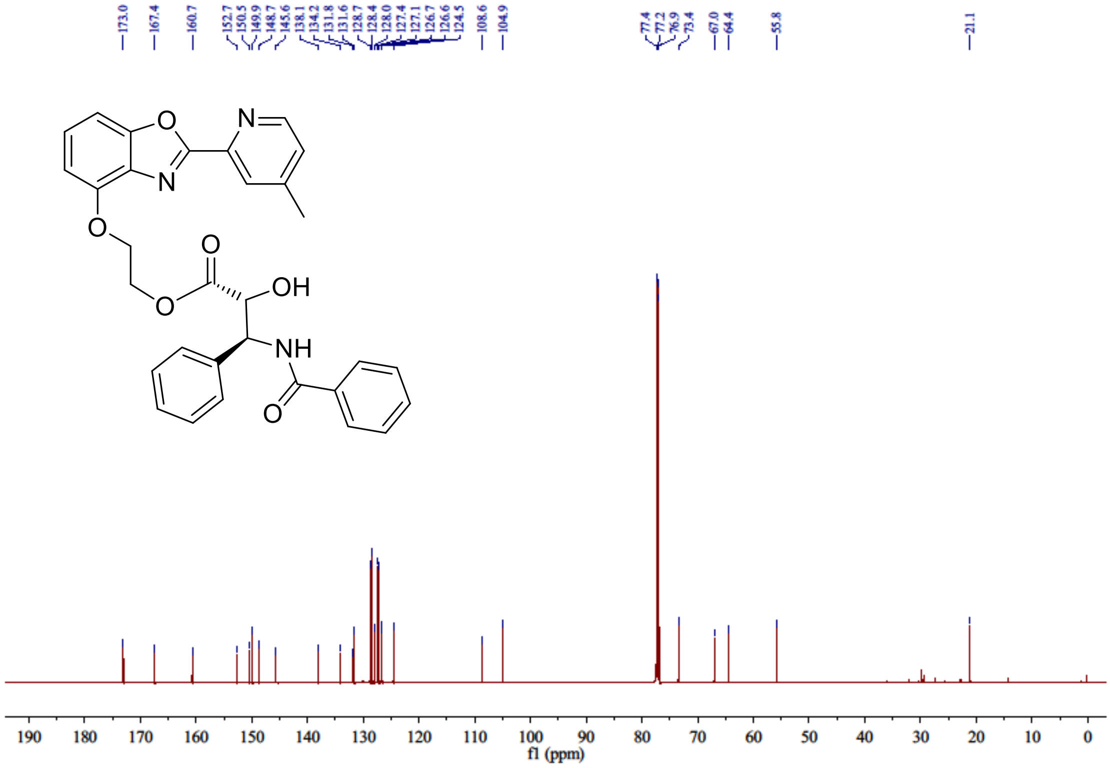

Figure S72 ^{13}C NMR (150 MHz) spectrum of compound **7j** in $CDCl_3$

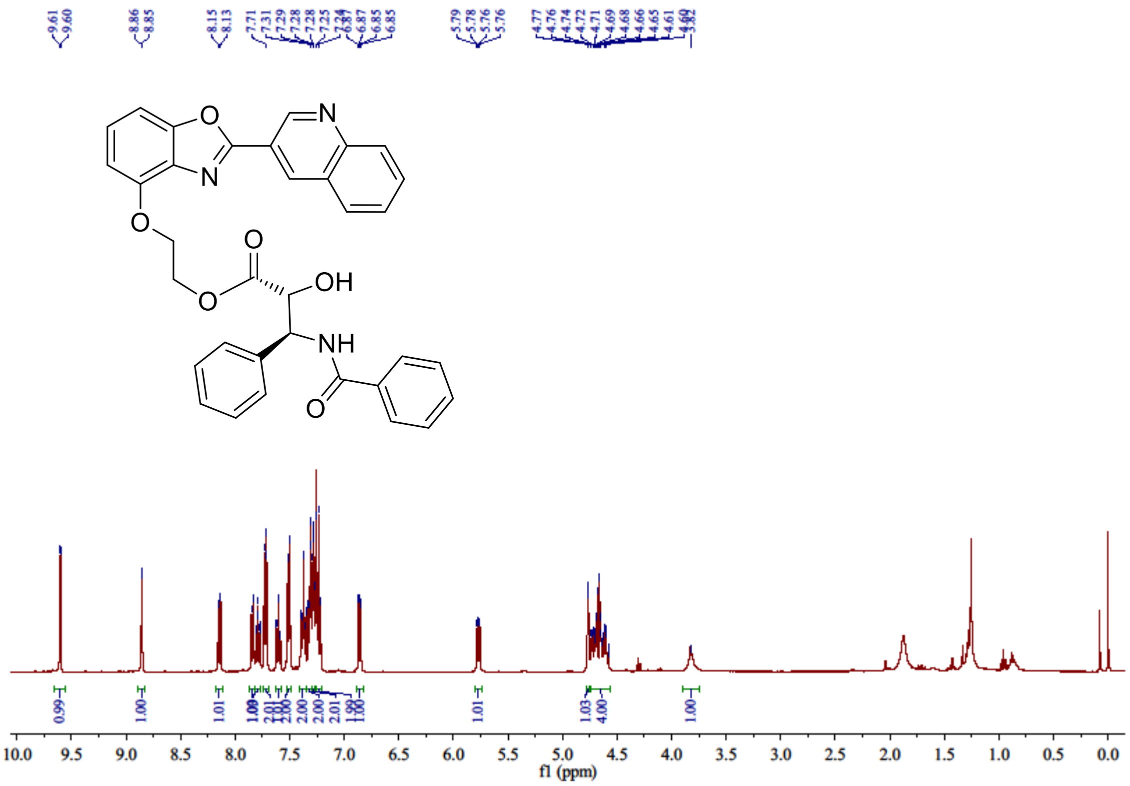

Figure S73 ^{1}H NMR (400 MHz) spectrum of compound **7k** in $CDCl_3$

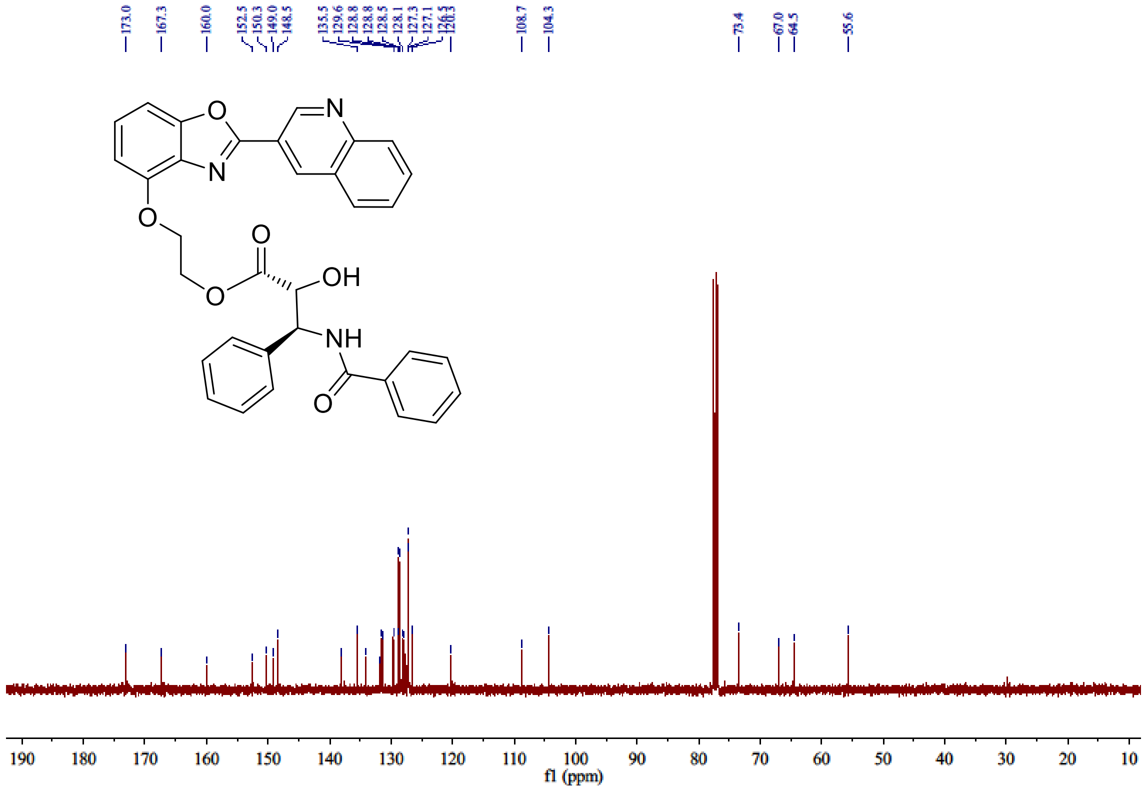

Figure S74 ^{13}C NMR (100 MHz) spectrum of compound **7k** in $CDCl_3$

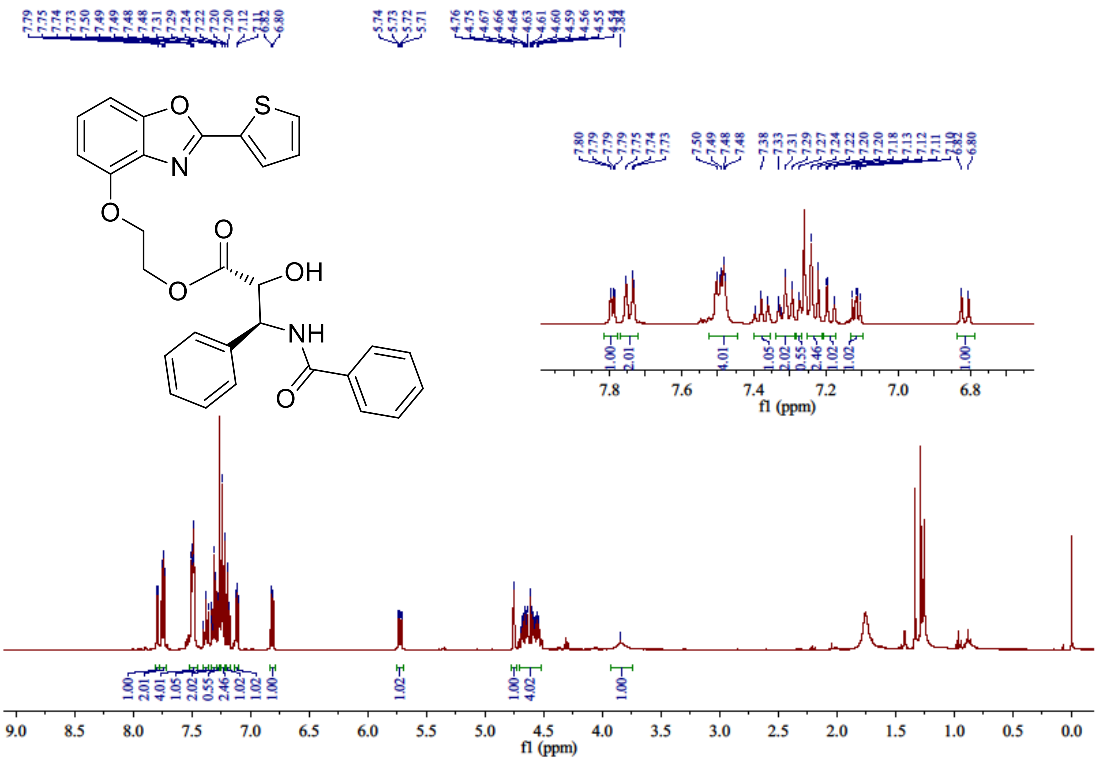

Figure S75 ^{1}H NMR (400 MHz) spectrum of compound **7l** in $CDCl_3$

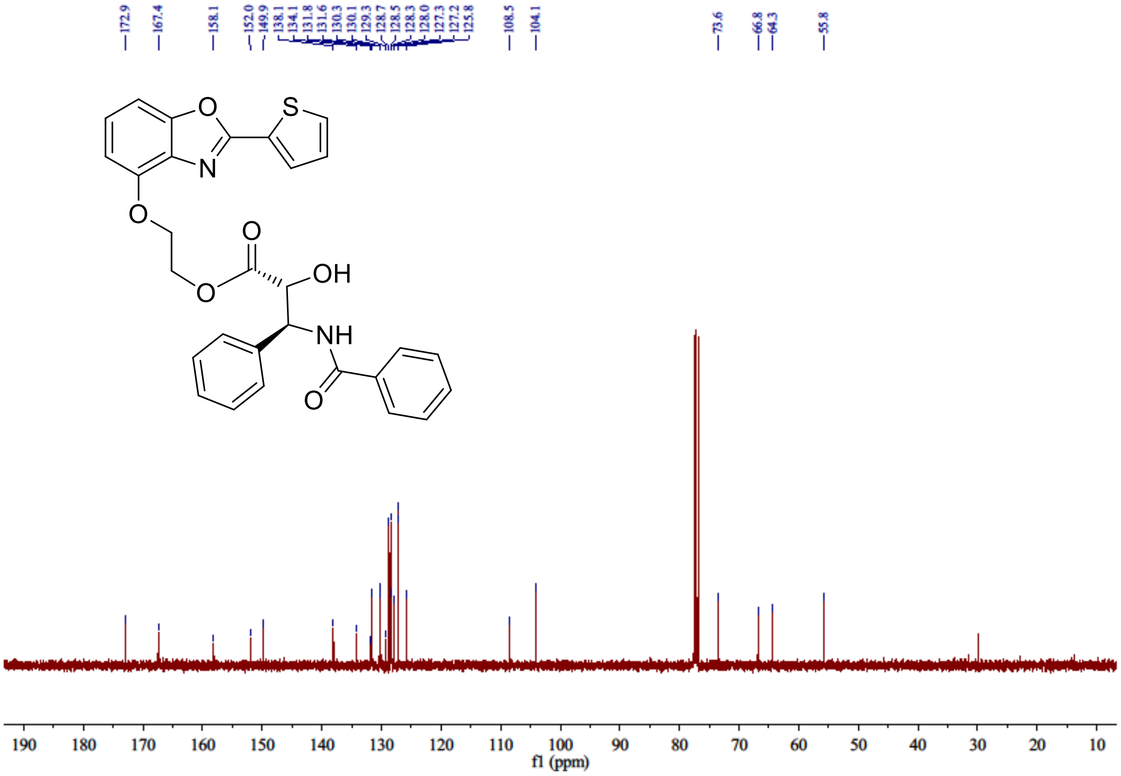

Figure S76 ^{13}C NMR (100 MHz) spectrum of compound **7l** in $CDCl_3$

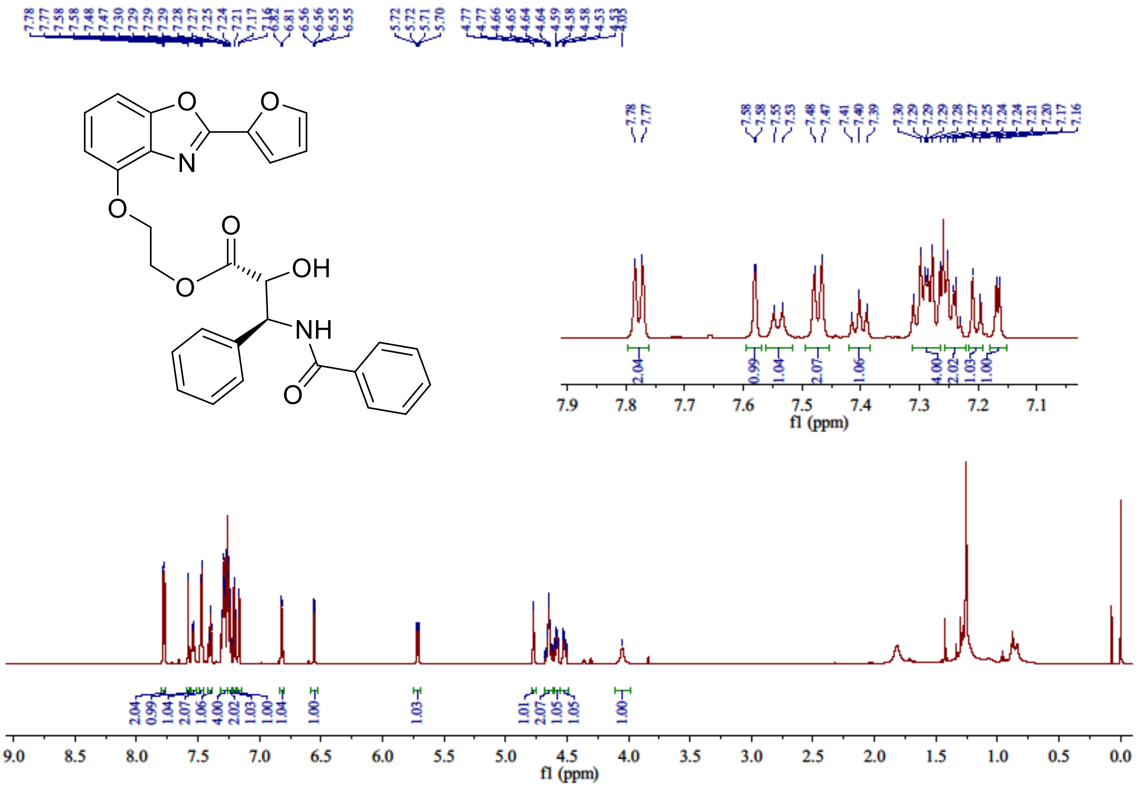

Figure S77 ^{1}H NMR (600 MHz) spectrum of compound **7m** in $CDCl_3$

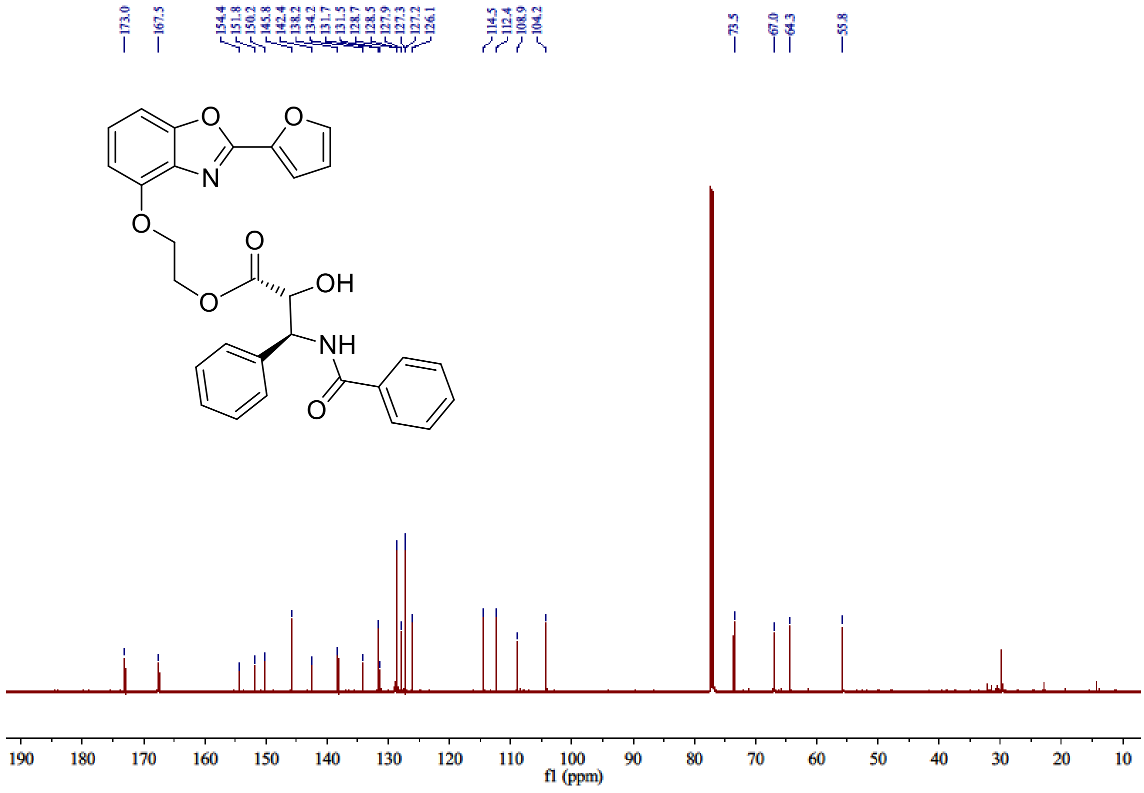

Figure S78 ^{13}C NMR (150 MHz) spectrum of compound **7m** in $CDCl_3$

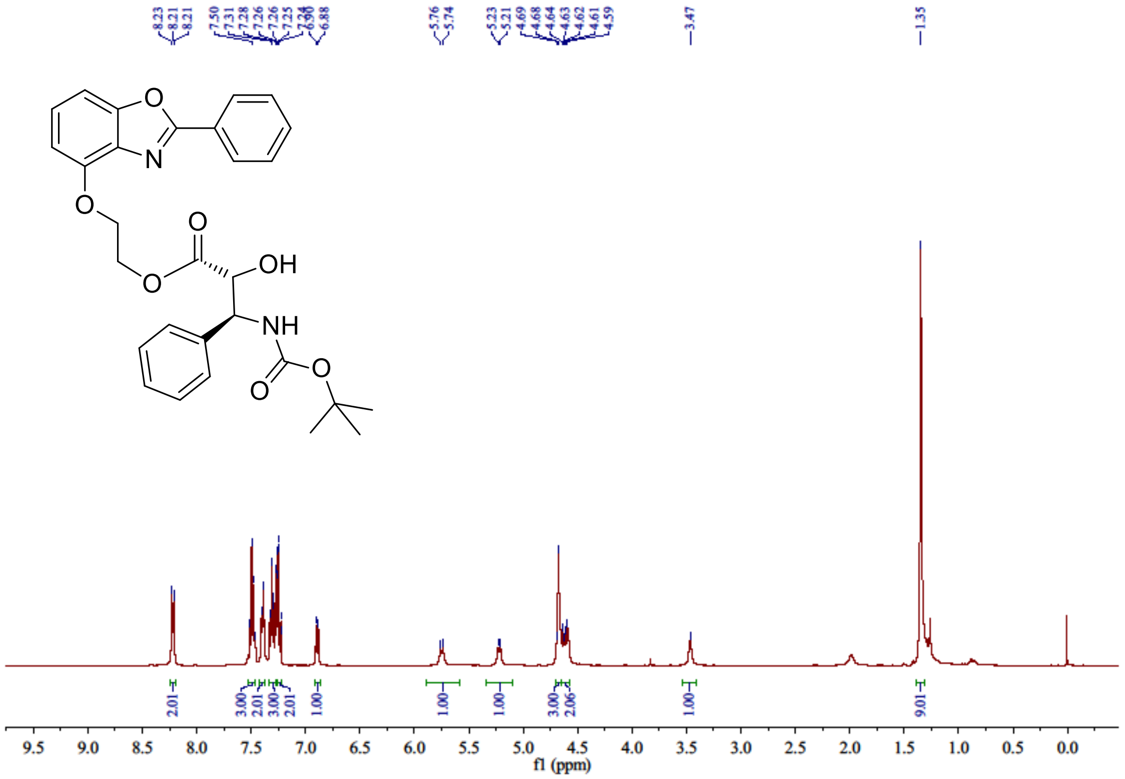

Figure S79 ^{1}H NMR (400 MHz) spectrum of compound **7A** in $CDCl_3$

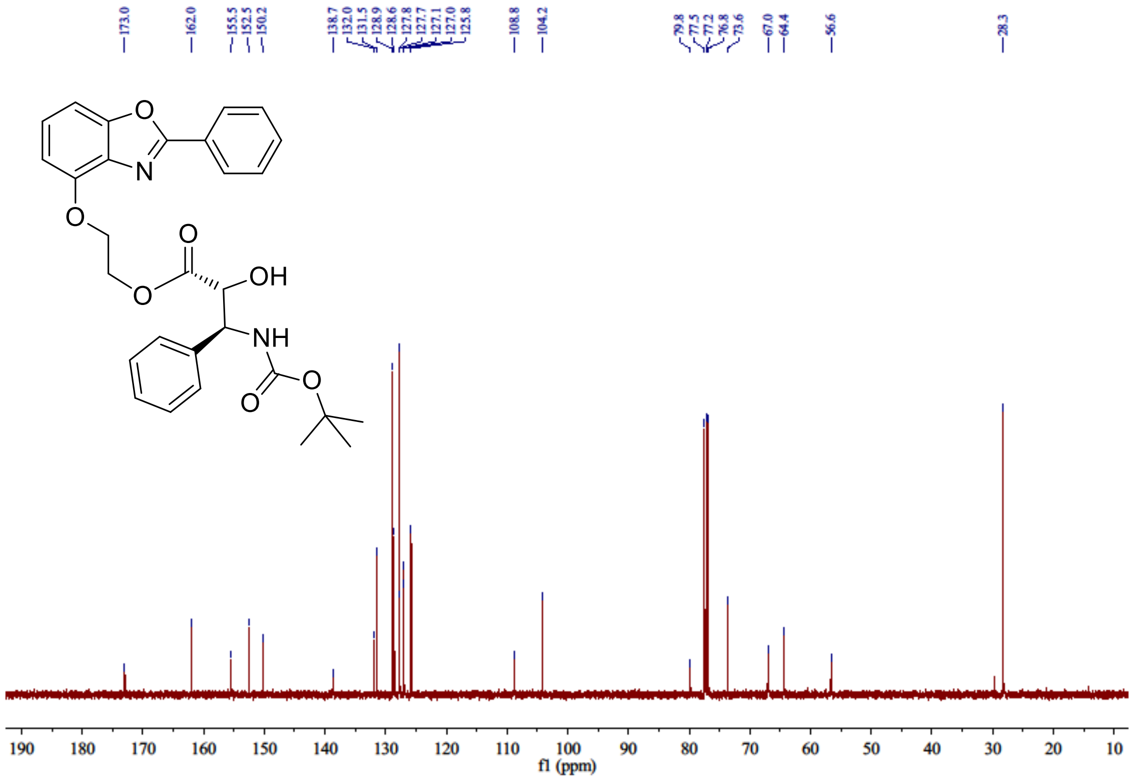

Figure S80 ^{13}C NMR (100 MHz) spectrum of compound **7A** in $CDCl_3$

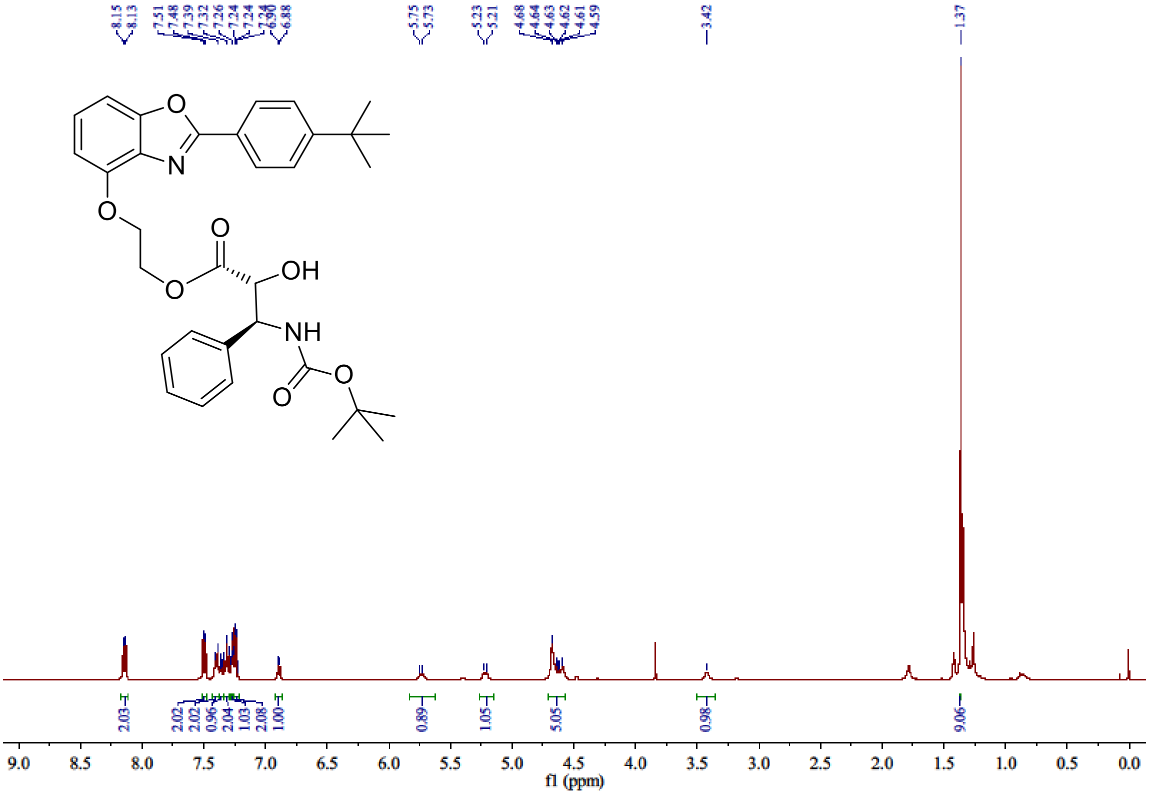

Figure S81 ^{1}H NMR (400 MHz) spectrum of compound **7B** in $CDCl_3$

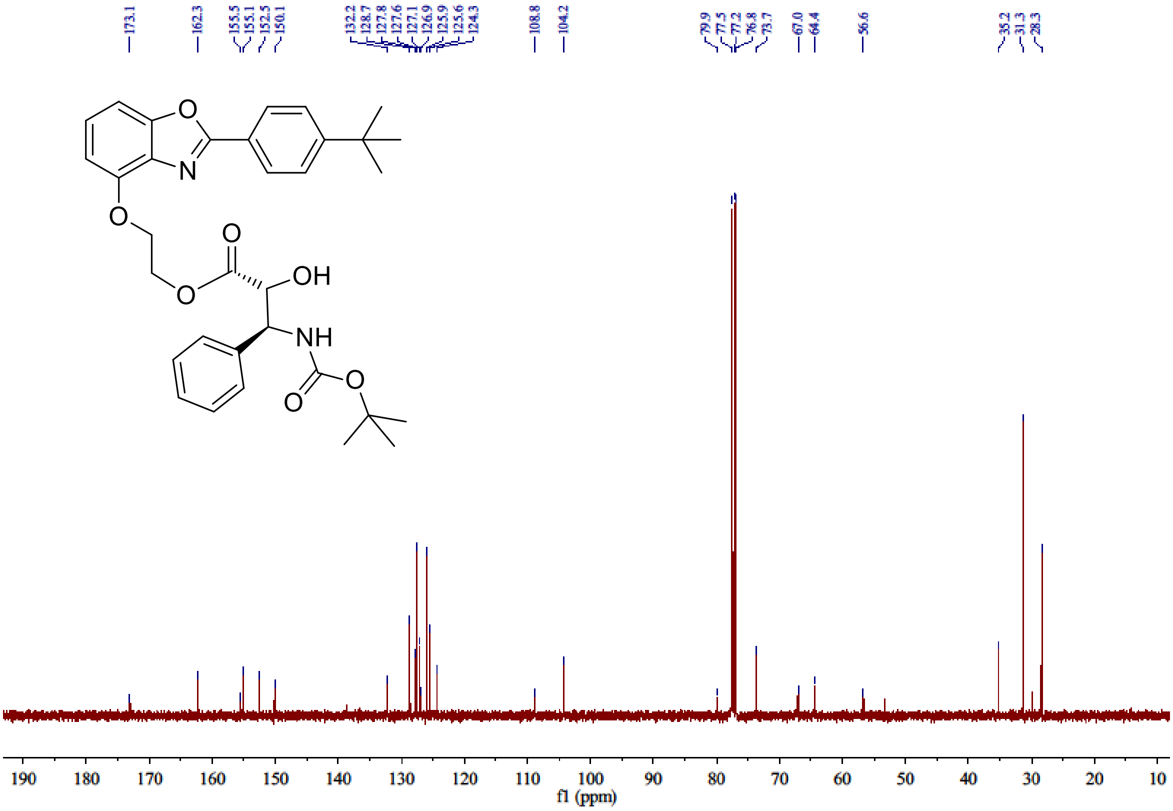

Figure S82 ^{13}C NMR (100 MHz) spectrum of compound **7B** in $CDCl_3$

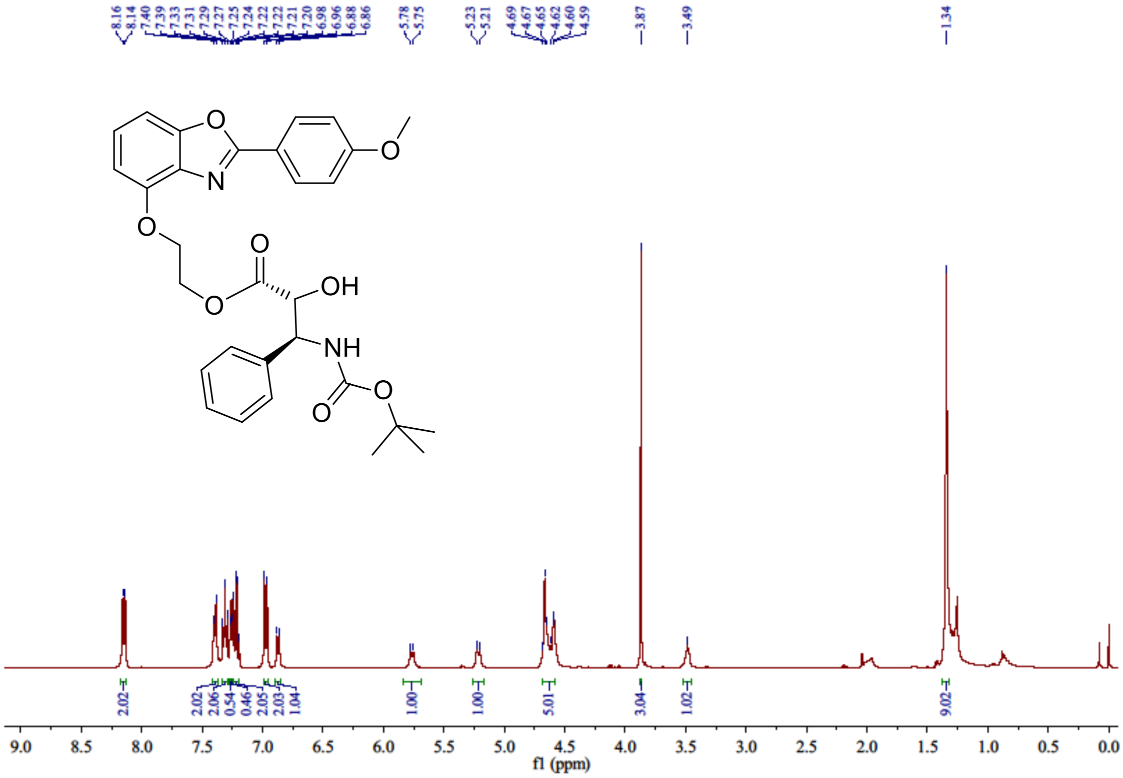

Figure S83 ^{1}H NMR (400 MHz) spectrum of compound **7C** in $CDCl_3$

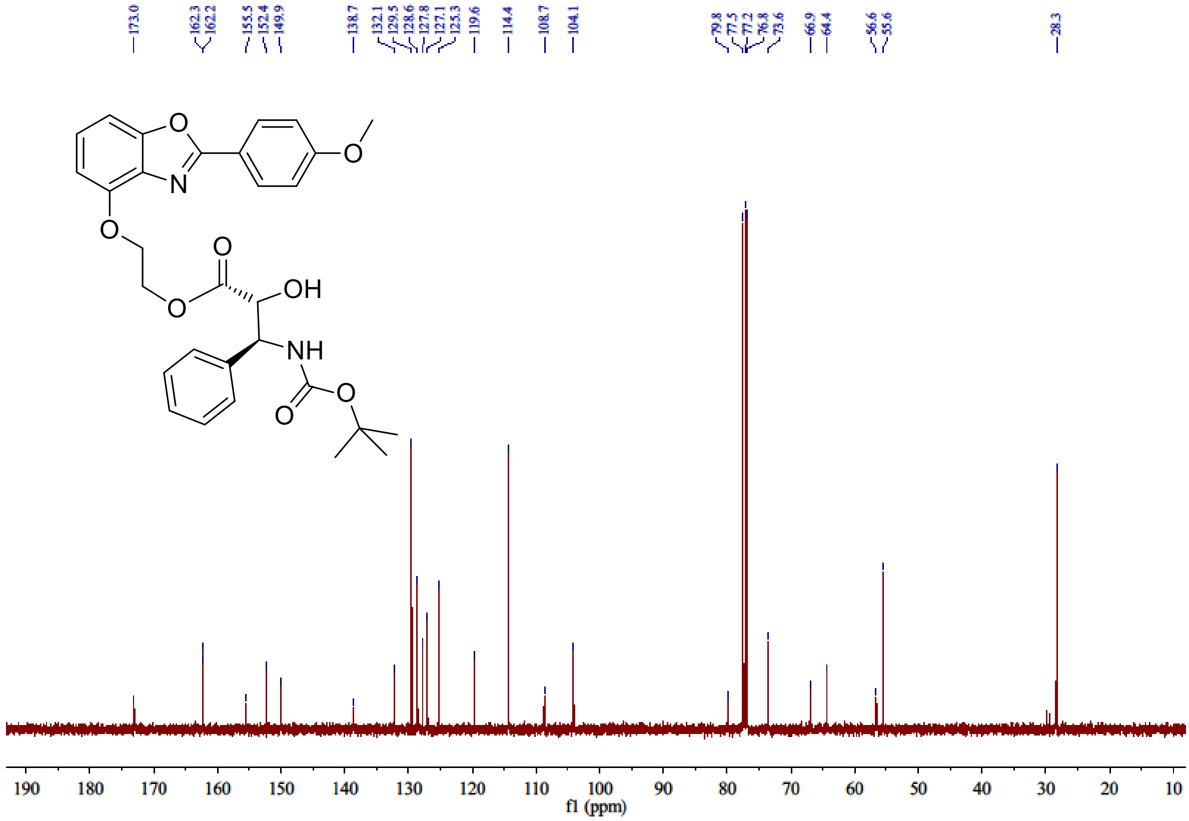

Figure S84 ^{13}C NMR (100 MHz) spectrum of compound **7C** in $CDCl_3$

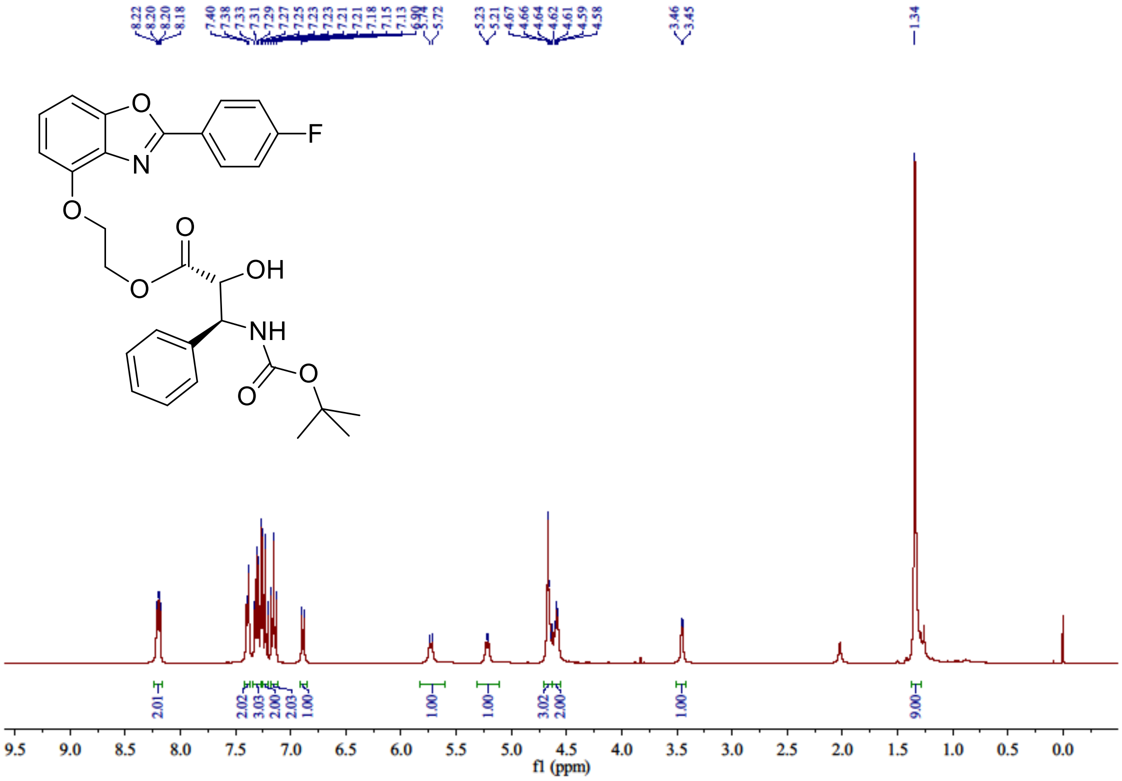

Figure S85 ^{1}H NMR (400 MHz) spectrum of compound **7D** in $CDCl_3$

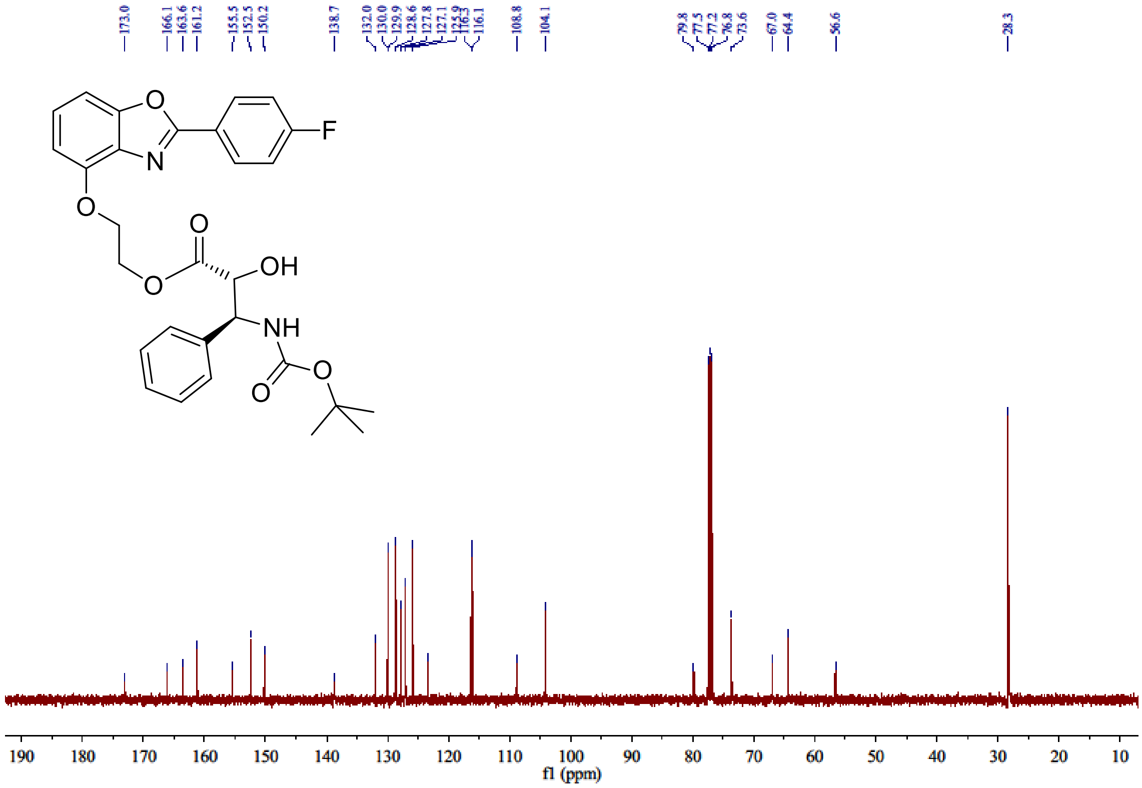

Figure S86 ^{13}C NMR (100 MHz) spectrum of compound **7D** in $CDCl_3$

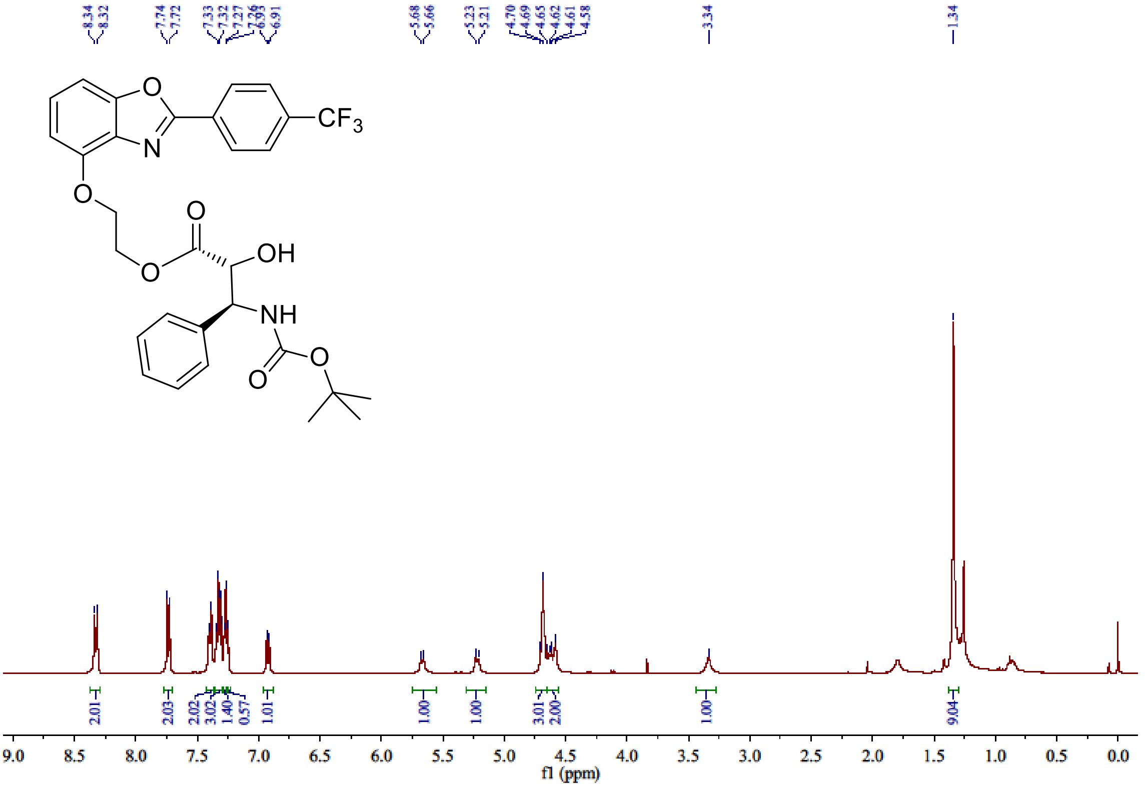

Figure S87 ^{1}H NMR (400 MHz) spectrum of compound **7E** in $CDCl_3$

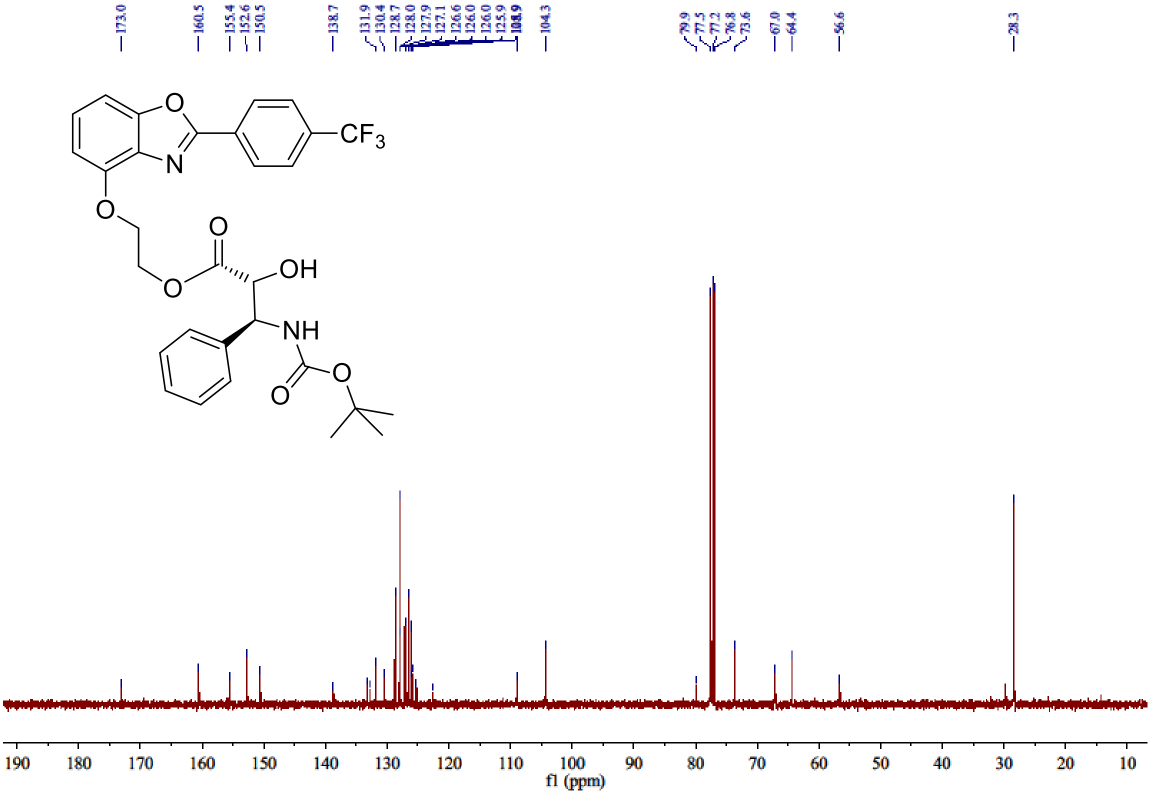

Figure S88 ^{13}C NMR (100 MHz) spectrum of compound **7E** in $CDCl_3$

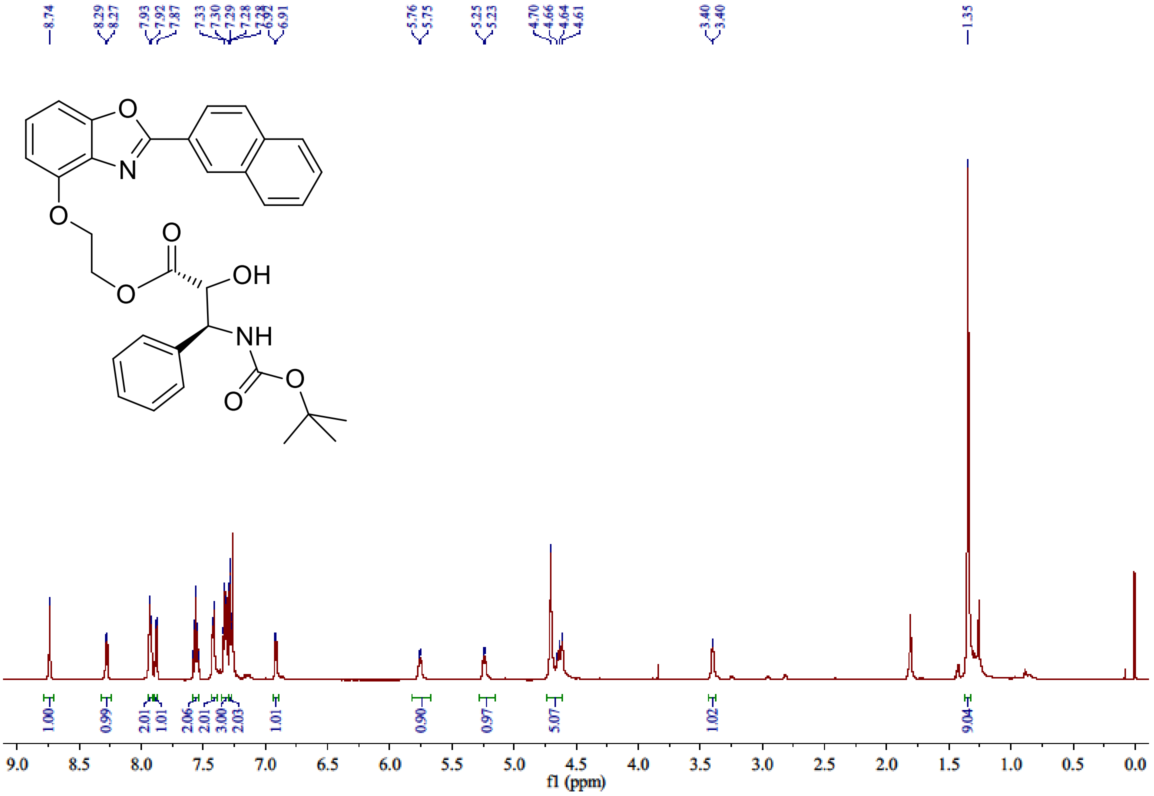

Figure S89 ^{1}H NMR (600 MHz) spectrum of compound **7F** in $CDCl_3$

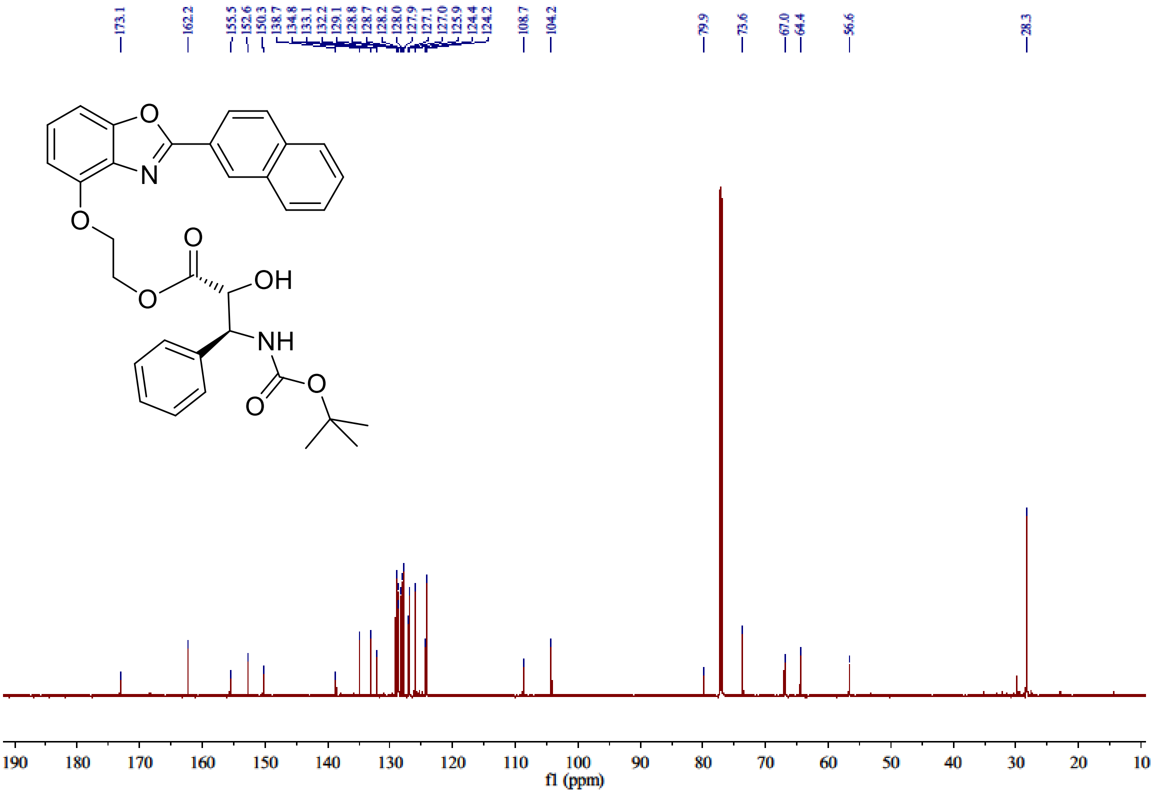

Figure S90 ^{13}C NMR (150 MHz) spectrum of compound **7F** in $CDCl_3$

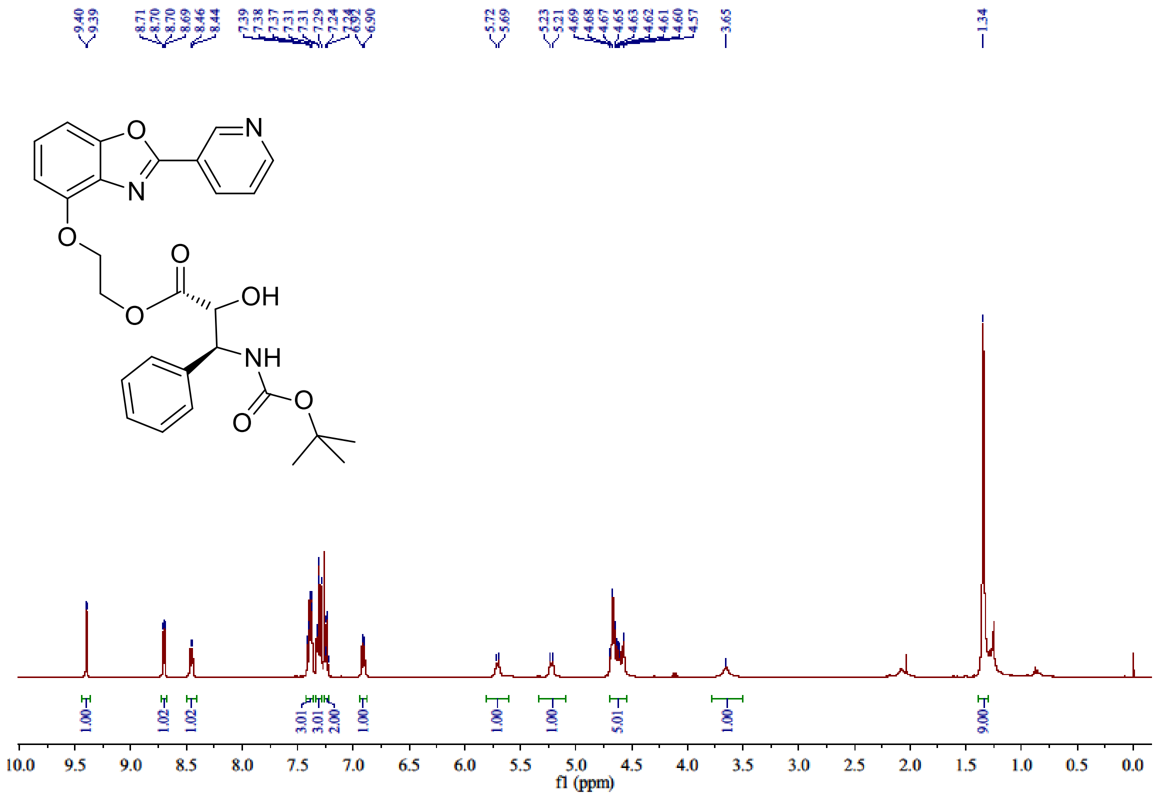

Figure S91 ^{1}H NMR (400 MHz) spectrum of compound **7G** in $CDCl_3$

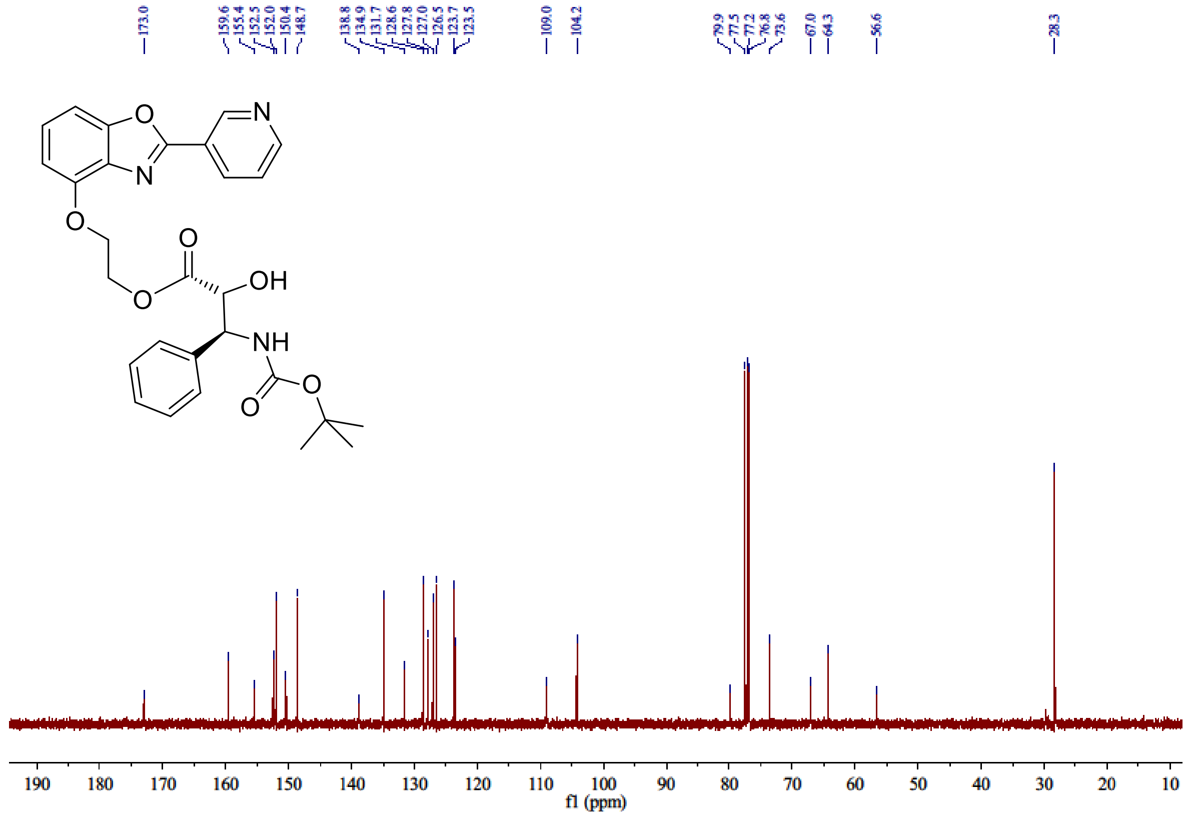

Figure S92 ^{13}C NMR (100 MHz) spectrum of compound **7G** in $CDCl_3$

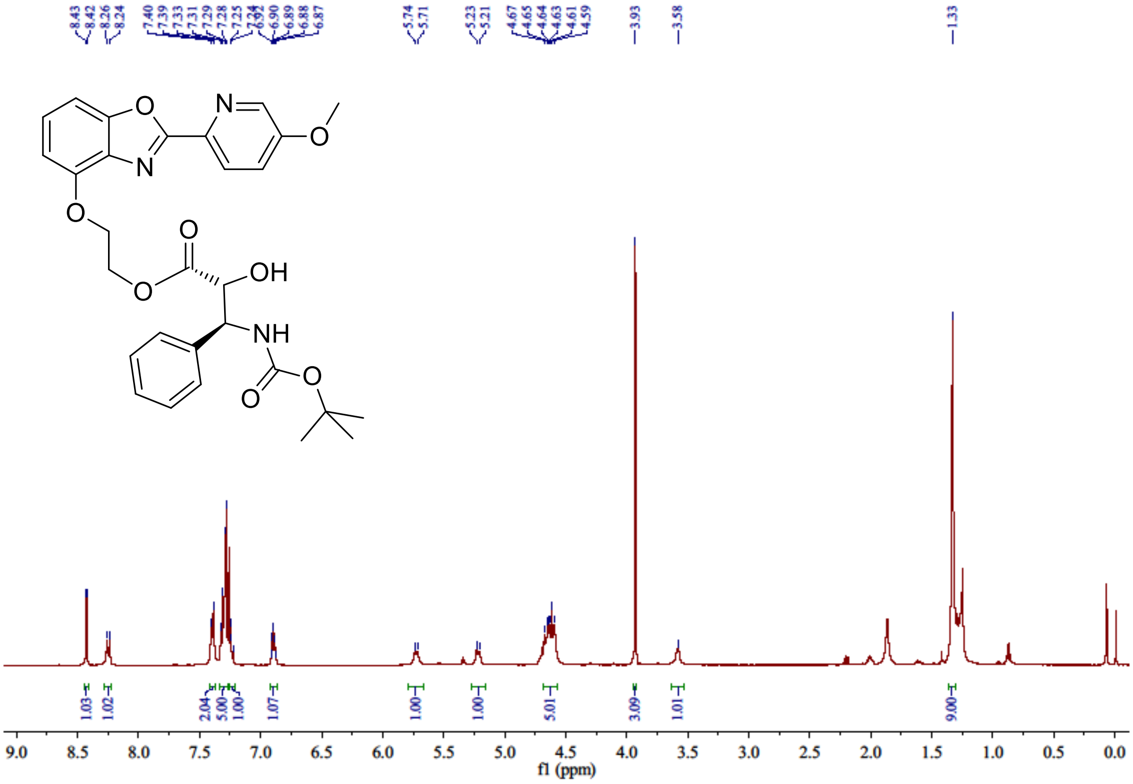

Figure S93 ^{1}H NMR (400 MHz) spectrum of compound **7H** in $CDCl_3$

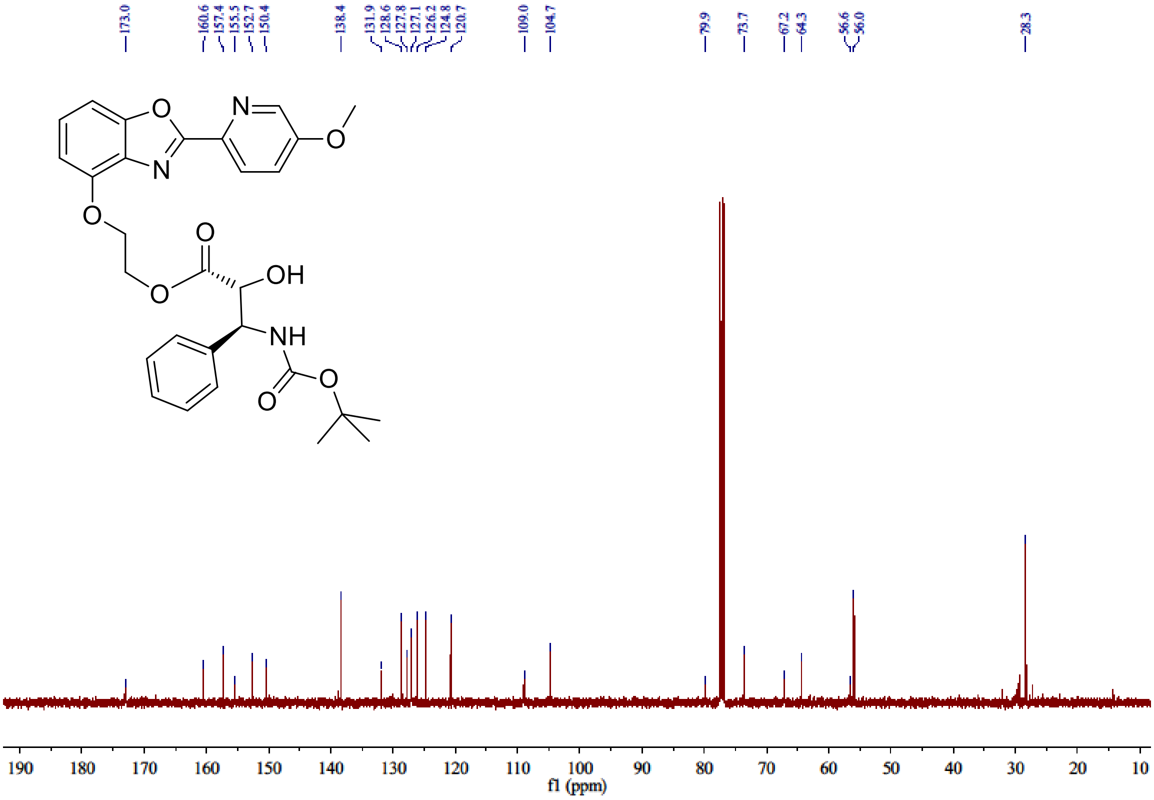

Figure S94 ^{13}C NMR (100 MHz) spectrum of compound **7H** in $CDCl_3$

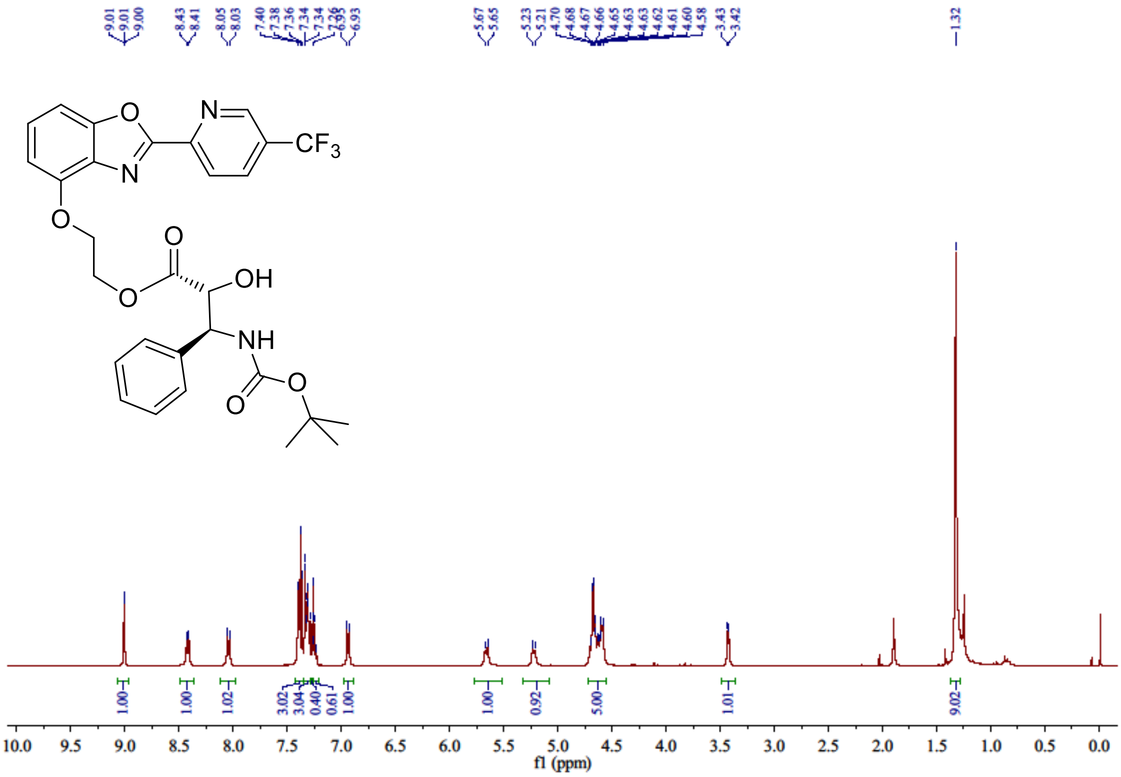

Figure S95 ^{1}H NMR (400 MHz) spectrum of compound **7I** in $CDCl_3$

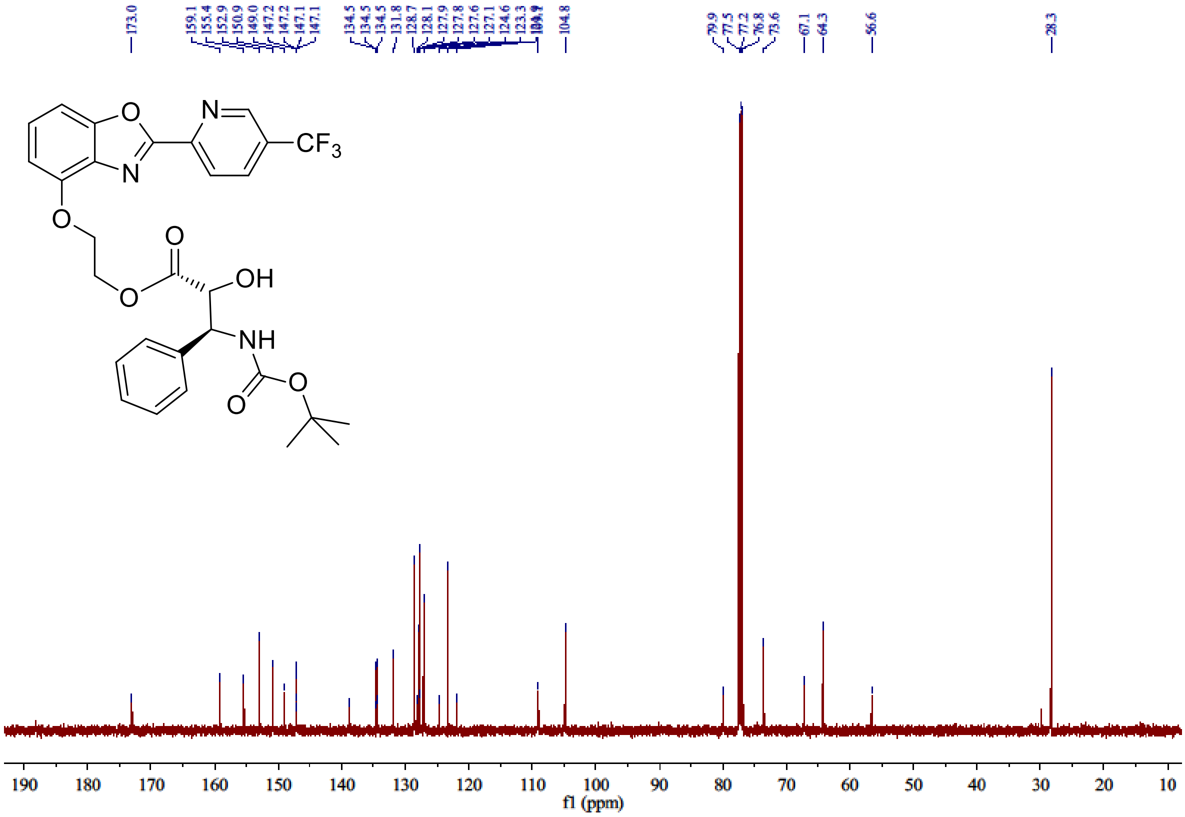

Figure S96 ^{13}C NMR (100 MHz) spectrum of compound **7I** in $CDCl_3$

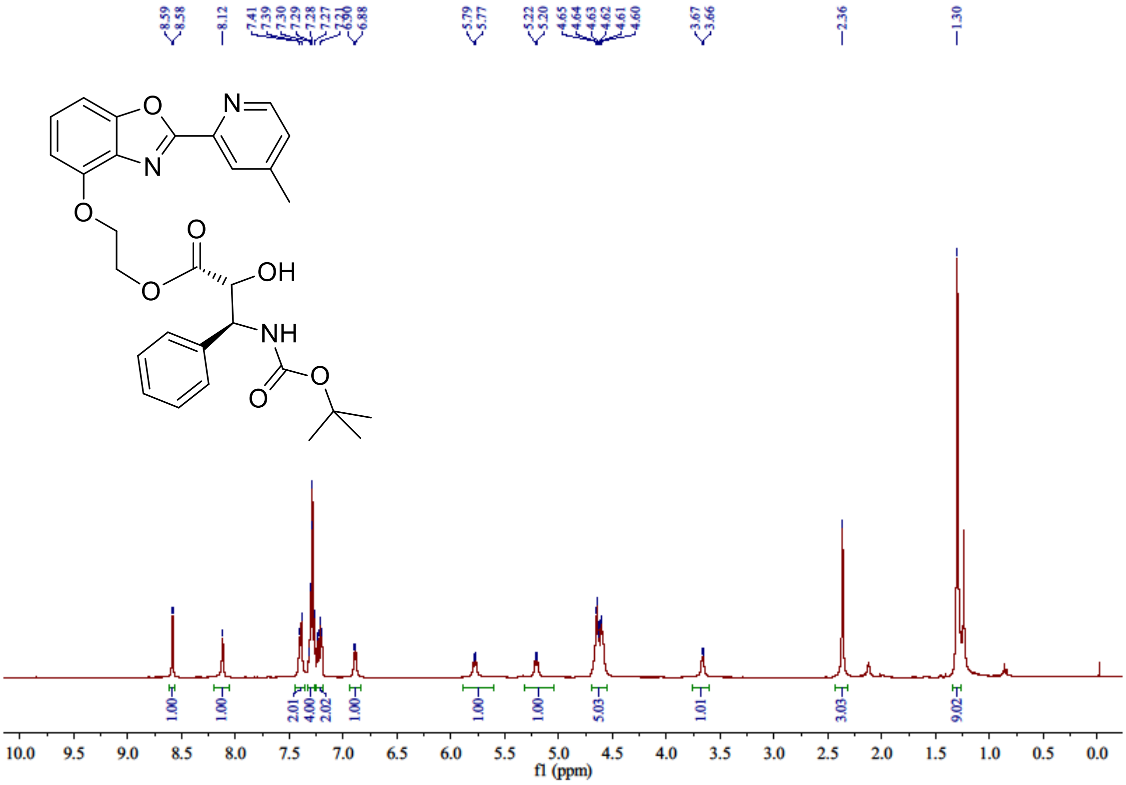

Figure S97 ^{1}H NMR (400 MHz) spectrum of compound **7J** in $CDCl_3$

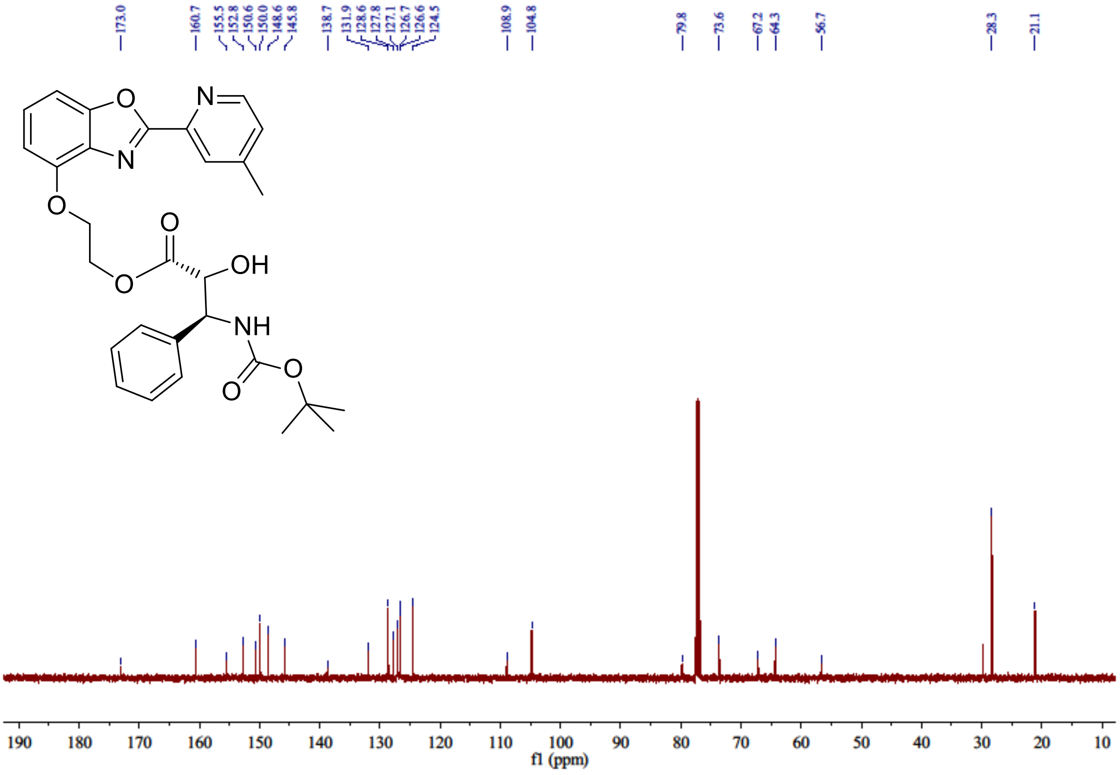

Figure S98 ^{13}C NMR (100 MHz) spectrum of compound **7J** in $CDCl_3$

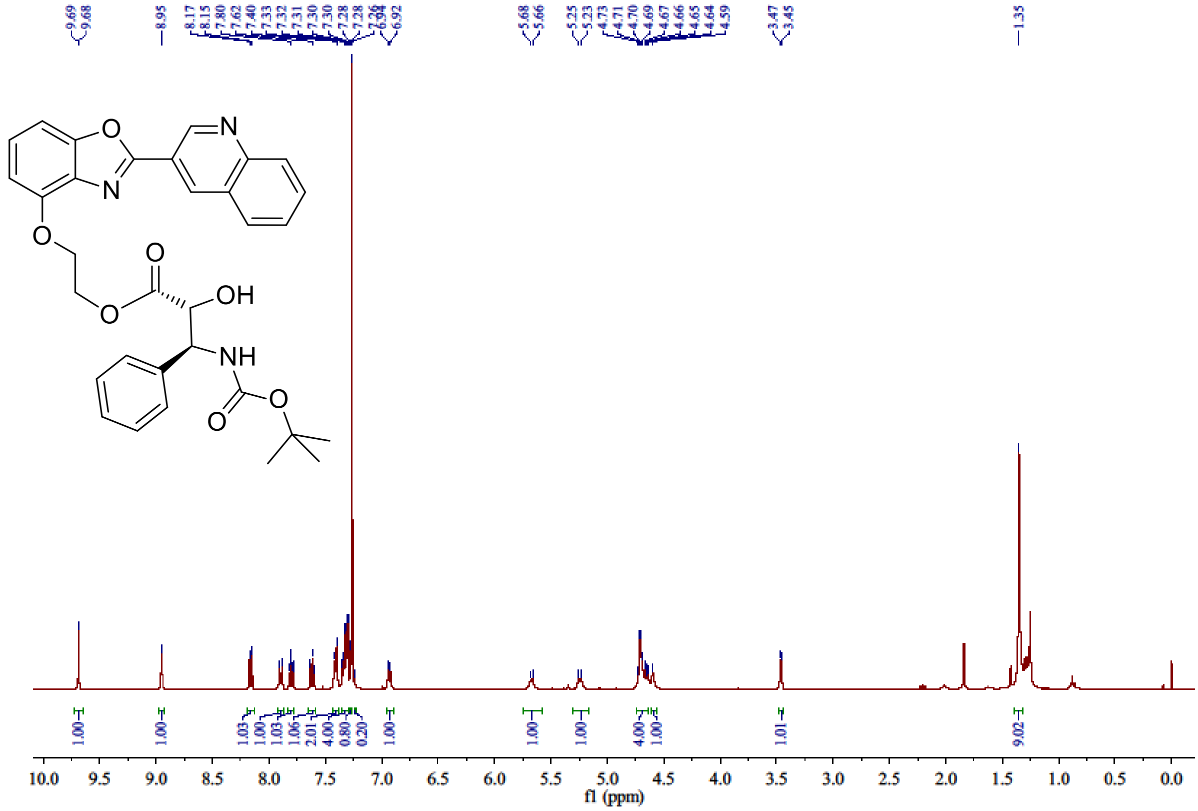

Figure S99 ^{1}H NMR (400 MHz) spectrum of compound **7K** in $CDCl_3$

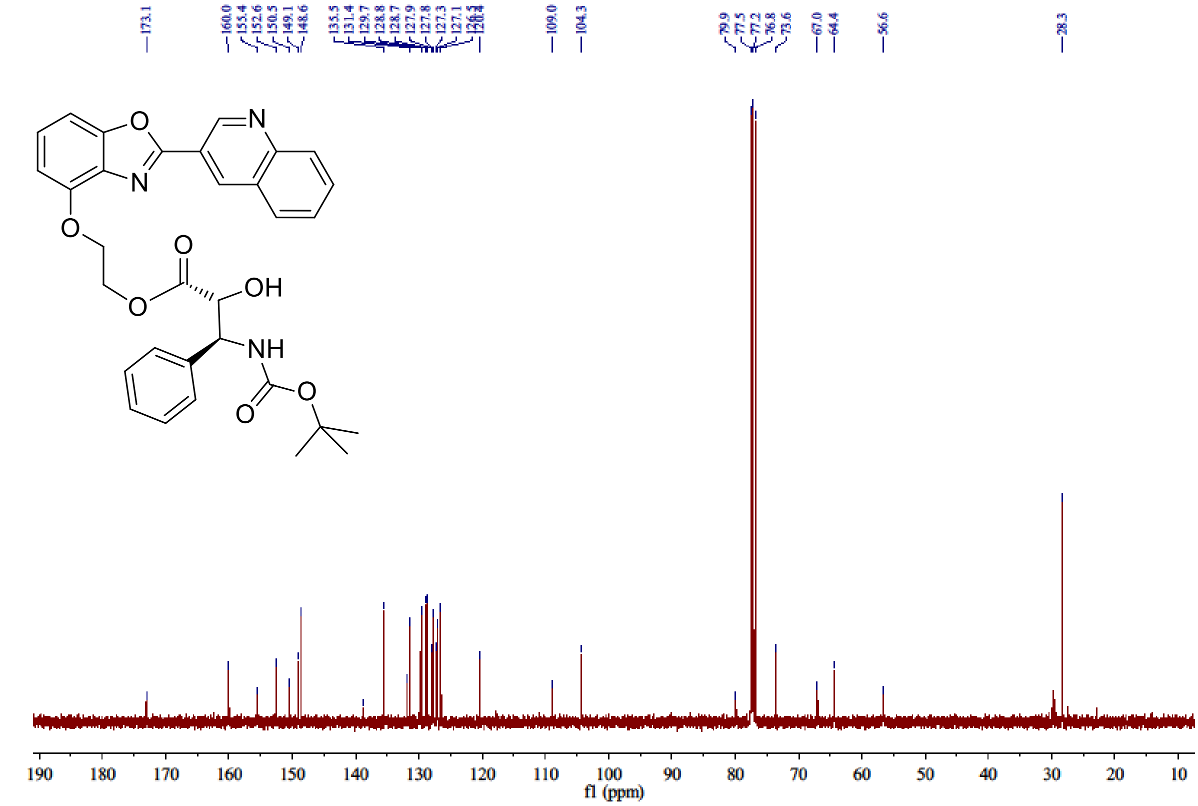

Figure S100 ^{13}C NMR (100 MHz) spectrum of compound **7K** in $CDCl_3$

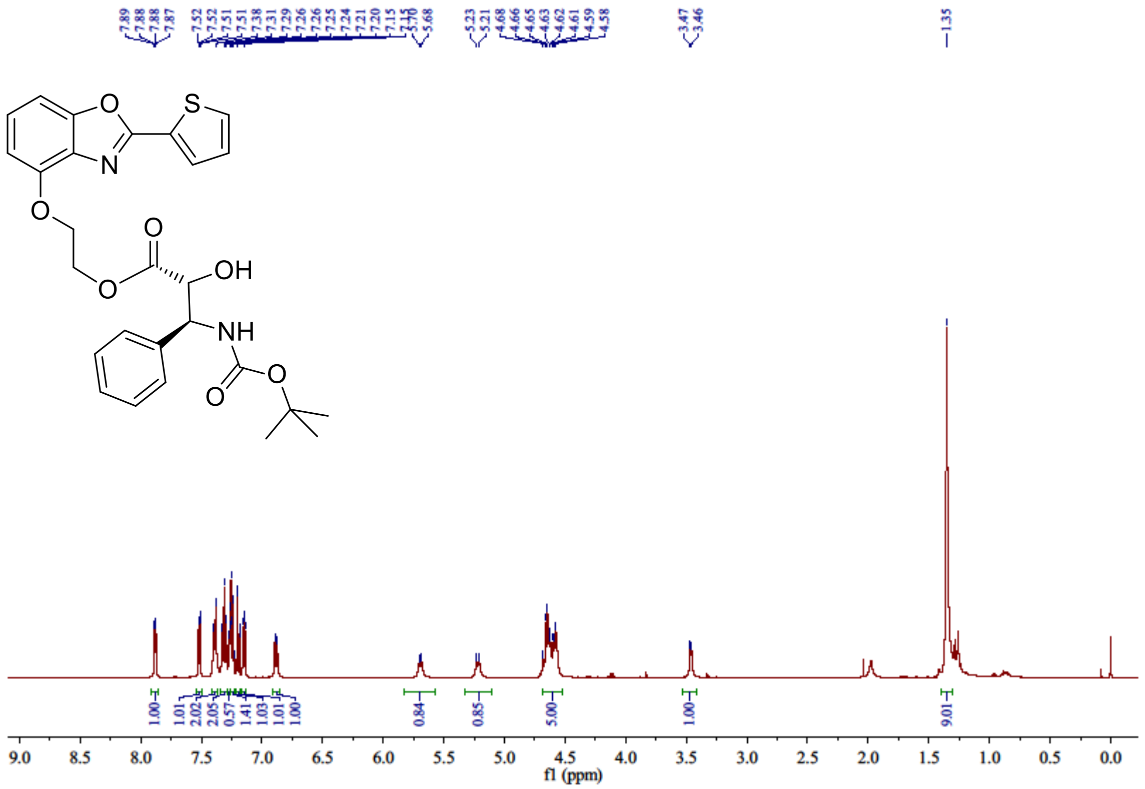

Figure S101 ^{1}H NMR (400 MHz) spectrum of compound **7L** in $CDCl_3$

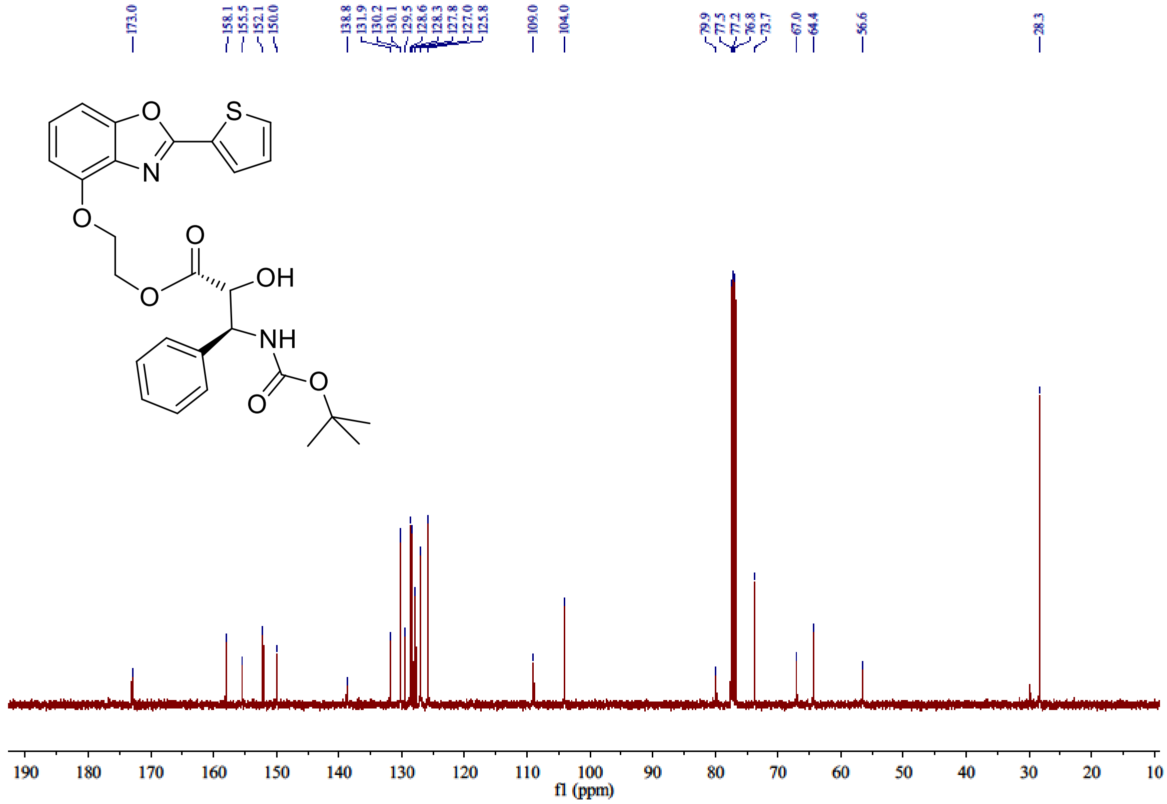

Figure S102 ^{13}C NMR (100 MHz) spectrum of compound **7L** in $CDCl_3$

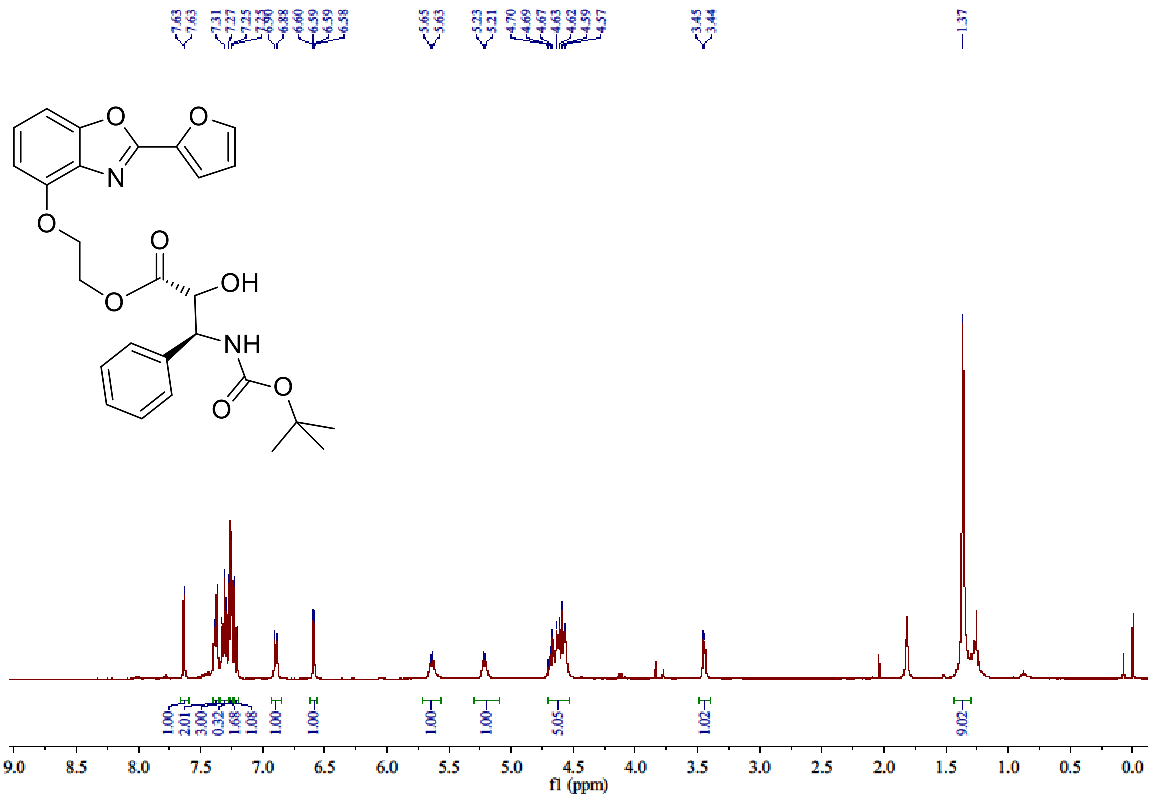

Figure S103 ^{1}H NMR (400 MHz) spectrum of compound **7M** in $CDCl_3$

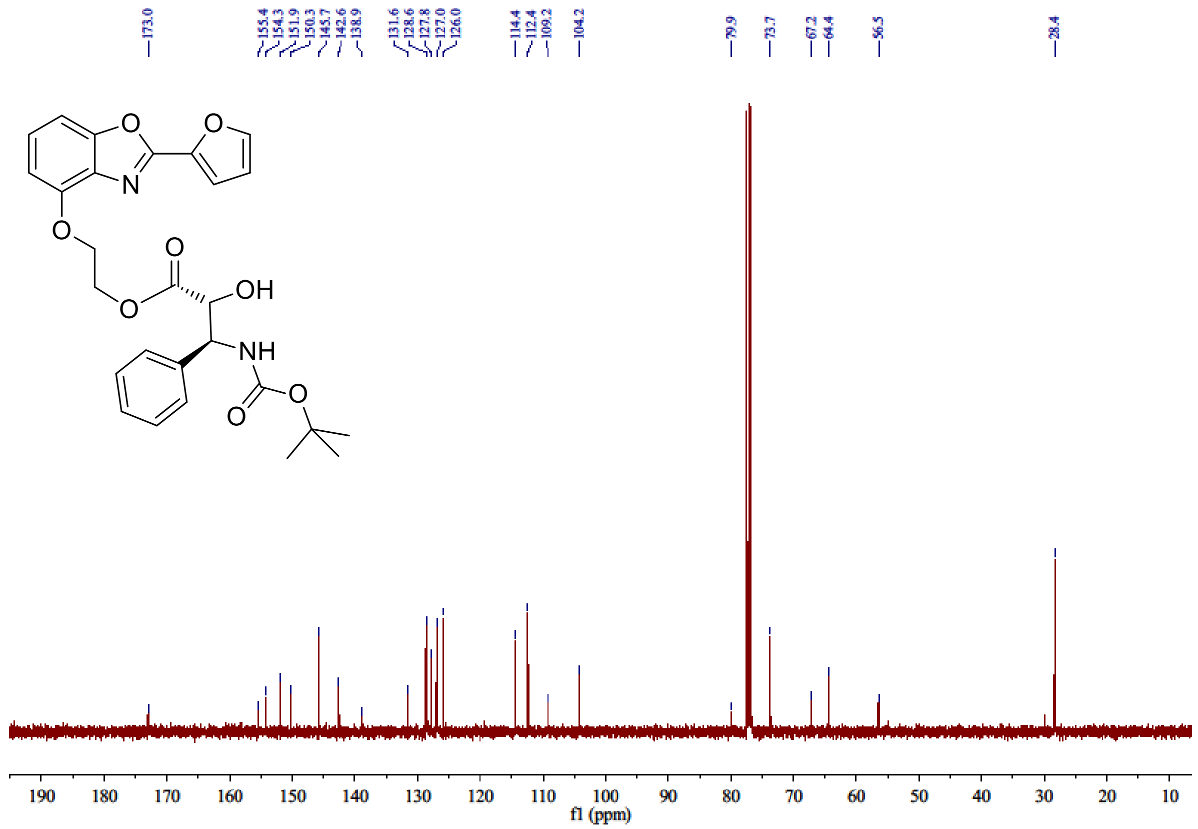

Figure S104 ^{13}C NMR (100 MHz) spectrum of compound **7M** in $CDCl_3$
